# Accounting for genetic differences among unknown parents in microevolutionary studies: how to include genetic groups in quantitative genetic animal models

**DOI:** 10.1111/1365-2656.12597

**Published:** 2016-11-03

**Authors:** Matthew E. Wolak, Jane M. Reid

**Affiliations:** ^1^ Institute of Biological and Environmental SciencesSchool of Biological SciencesUniversity of Aberdeen, Zoology Building, Tillydrone AvenueAberdeen AB24 2TZUK

**Keywords:** ASReml, base population, dispersal, heritability, MCMCglmm, nadiv, numerator relationship matrix, phantom parents, total additive genetic effects, WOMBAT

## Abstract

1. Quantifying and predicting microevolutionary responses to environmental change requires unbiased estimation of quantitative genetic parameters in wild populations. ‘Animal models’, which utilize pedigree data to separate genetic and environmental effects on phenotypes, provide powerful means to estimate key parameters and have revolutionized quantitative genetic analyses of wild populations.
2. However, pedigrees collected in wild populations commonly contain many individuals with unknown parents. When unknown parents are non‐randomly associated with genetic values for focal traits, animal model parameter estimates can be severely biased. Yet, such bias has not previously been highlighted and statistical methods designed to minimize such biases have not been implemented in evolutionary ecology.
3. We first illustrate how the occurrence of non‐random unknown parents in population pedigrees can substantially bias animal model predictions of breeding values and estimates of additive genetic variance, and create spurious temporal trends in predicted breeding values in the absence of local selection. We then introduce ‘genetic group’ methods, which were developed in agricultural science, and explain how these methods can minimize bias in quantitative genetic parameter estimates stemming from genetic heterogeneity among individuals with unknown parents.
4. We summarize the conceptual foundations of genetic group animal models and provide extensive, step‐by‐step tutorials that demonstrate how to fit such models in a variety of software programs. Furthermore, we provide new functions in r that extend current software capabilities and provide a standardized approach across software programs to implement genetic group methods.
5. Beyond simply alleviating bias, genetic group animal models can directly estimate new parameters pertaining to key biological processes. We discuss one such example, where genetic group methods potentially allow the microevolutionary consequences of local selection to be distinguished from effects of immigration and resulting gene flow.
6. We highlight some remaining limitations of genetic group models and discuss opportunities for further development and application in evolutionary ecology. We suggest that genetic group methods should no longer be overlooked by evolutionary ecologists, but should become standard components of the toolkit for animal model analyses of wild population data sets.

## Introduction

Adaptive evolution is a critical way by which populations can respond to environmental change and persist. Quantifying and predicting microevolutionary responses to environmental change in wild populations is consequently a major focus in biology (Nussey *et al*. 2005; Gienapp *et al*. 2008; Hoffmann & Sgrò 2011). Empirical studies must tease apart environmental and genetic contributions to overall phenotypic variation and quantify selection acting on each component (Postma 2006; Gienapp *et al*. 2008; Hadfield *et al*. 2010). This in turn requires unbiased estimation of key quantitative genetic parameters, such as heritabilities and additive genetic (co)variances.

Application of ‘animal models’ (linear mixed models that quantify genetic effects at the level of individuals) has revolutionized quantitative genetic studies of wild populations (Kruuk 2004; Wilson *et al*. 2010; Charmantier, Garant & Kruuk 2014). Animal models facilitate estimation of additive genetic variance by (potentially) separating phenotypic resemblance among individuals arising from direct additive genetic effects, environmental similarities (Kruuk & Hadfield 2007; Stopher *et al*. 2012), indirect genetic effects (Moore, Brodie & Wolf 1997; Kruuk & Hadfield 2007; Wilson *et al*. 2011), and inbreeding and non‐additive genetic effects (Kennedy, Schaeffer & Sorensen 1988; Reid & Keller 2010; Wolak 2012; Wolak & Keller 2014). Such analyses traditionally require sufficient pedigree data to quantify ‘relatedness’ among individuals, allowing additive genetic variance to be estimated from a decomposition of the phenotypic similarity among relatives. Critical requirements of such pedigree data are that individuals are linked to their parents to form individual pedigrees and that individual pedigrees are linked across generations to form a population pedigree. Animal models then estimate key parameters relative to a defined ‘base population’, which in practice comprises the ‘phantom parents’ of all individuals whose true parents are unknown or not identified in the pedigree (key terms are defined in the Glossary, Appendix S1, Supporting Information; Quaas 1988; Westell, Quaas & Van Vleck 1988).

**Figure 1:**
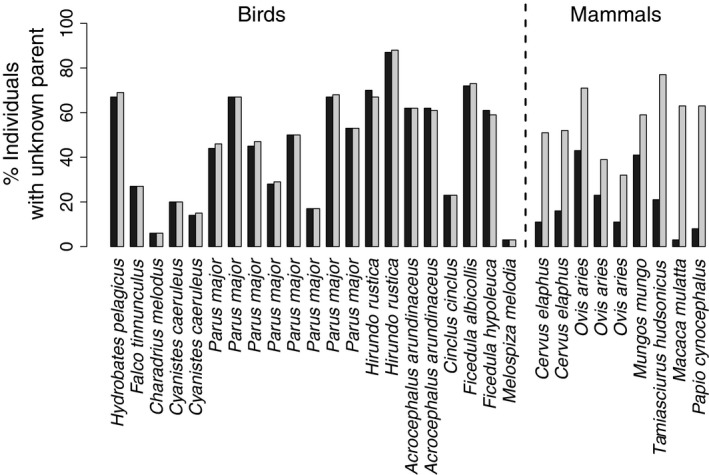
Percentages of pedigreed individuals that have unknown dams (black bars) or sires (grey bars) in wild population pedigrees. Further details are in Appendix S2.

It is clear that pedigree error, where individuals are assigned the wrong parents, can bias quantitative genetic parameter estimates to some degree (Charmantier & Réale 2005; Morrissey *et al*. 2007; Morrissey & Wilson 2010; Reid *et al*. 2014; Firth *et al*. 2015). Inadequate pedigree depth, where individual pedigrees are not linked to multiple generations of ancestor pedigrees, can also cause bias by underestimating relatedness and impeding estimation of inbreeding effects, parental genetic and environmental effects, and microevolutionary change across generations (Cassell, Adamec & Pearson 2003; Kruuk & Hadfield 2007; Pemberton 2008). However, it is less commonly emphasized that missing pedigree information, where one or both of an individual's parents are unknown, might also severely bias quantitative genetic parameter estimates. Such biases surely need to be considered because some degree of missing pedigree information afflicts almost all wild population studies. Indeed, wild population pedigrees underlying recently published analyses were missing means of 37% of maternal identities (range = 3–87%) and 49% of paternal identities (range = 6–88%; Fig. 1, Appendix S2). Some analyses therefore relied on pedigrees that had more unknown parents than known parents. These counts include individuals from the ‘founder population’ that, by definition, have unknown parents (Glossary, Appendix S1). However, founders typically account for small proportions of individuals with unknown parents given overall pedigree sizes and maximum pedigree depths (Table S2.1).

Analyses implemented in agricultural sciences show that missing pedigree information stemming from unknown parents can bias animal model estimates of additive genetic variance (e.g. Dong, Van Vleck & Wiggans 1988; Cantet *et al*. 2000). Such biases can arise when parent identities are missing randomly with respect to genetic value, because the resulting pedigree underestimates relatedness and inbreeding. However, biases might be even more severe when parent identities are missing non‐randomly with respect to phenotypic or genetic values, which is likely to be commonplace in wild population studies (see Unknown parents in wild population studies). Fortunately, agricultural science has also shown how to account for missing pedigree information in the animal model by assigning the unknown parents to distinct ‘genetic groups’ (Quaas 1988). Genetic group methods allow genetic effects to be assigned to multiple groups within the base population with potentially different group means, thereby modelling genetic effects of different groups of individuals with unknown parents. However, genetic group animal models have not been widely used in evolutionary ecology (so far Charmantier *et al*. 2016 and Wolak & Reid 2016a have used the method on empirical data and the concept of genetic groups was used to illustrate challenges of breeding value prediction by Hadfield *et al*. 2010). Evolutionary ecologists might therefore be needlessly ignoring bias in key quantitative genetic parameter estimates when appropriate analytical remedies already exist. Furthermore, alongside statistical correction for non‐random missing pedigree information, genetic group methods enable direct estimation of quantitative genetic parameters pertaining to biological processes that cause individuals’ parents to be unknown (e.g. behaviour, reproductive strategy, dispersal). For example, genetic differences between a focal population and immigrants (which typically have unknown parents) can be estimated (e.g. Wolak & Reid 2016a), thereby quantifying the relative contributions of local selection and gene flow to phenotypic change. By failing to implement genetic group methods, evolutionary ecologists are missing an opportunity to directly quantify key processes that might drive or impede microevolutionary change.

We introduce genetic group methods and explain how they can be incorporated into animal models to analyse wild population data. First, we highlight ways in which unknown parents and corresponding missing pedigree information can arise in wild population studies. Secondly, we summarize key attributes of animal models that can cause problems when pedigrees contain unknown parents and illustrate how missing pedigree information can bias estimates of heritabilities and additive genetic variances. Thirdly, we summarize genetic group methods, explain how such methods can both reduce bias and answer biologically interesting questions and demonstrate how they can be implemented in widely used software programs. Finally, we discuss limitations of current genetic group methods and highlight requirements and opportunities for future investigation into the impacts of missing pedigree information and the implementation of genetic group methodologies in evolutionary ecology.

## Unknown parents in wild population studies

The extent to which animal model parameter estimates are biased by missing pedigree information depends on how many non‐founder individuals have unknown parents and on the degree to which ‘breeding values’ of non‐founder ‘phantom parents’ differ from those of the true base population. Non‐founder individuals can have unknown parents for numerous practical and biological reasons. For example, field studies typically monitor finite subsets of population members or breeding sites and consequently do not observe parents of individuals born or hatched outwith the focal subset that subsequently breed within it. Further, the ability to observe or genotype an individual for parentage assignment might depend on the individual's own behavioural or life‐history phenotype. For example, individuals that differ in boldness or aggressiveness might differ in conspicuousness, approachability, or trapability (Biro & Dingemanse 2009), making some individuals harder to tag or identify and excluding them as known or candidate parents. Likewise, parents that breed successfully might be more likely to be observed (e.g. Kidd *et al*. 2015). Consequently, observation probability might covary with factors influencing reproductive success, such as age or breeding site choice (e.g. Forslund & Pärt 1995; Öst & Steele 2010). Also, reproductive behaviour and habitat segregation commonly facilitate observation of one parental sex (Ruckstuhl & Neuhaus 2005); hence, maternities are often more readily assigned than paternities (e.g. Sardell *et al*. 2010; Walling *et al*. 2010).

Even if all or most population members can be observed and genotyped, available genotypic data might be insufficient to confidently assign parentage among closely related candidates. Heritable variation in fitness might then cause non‐random failure to assign parents to their offspring. For example, paternity might not be confidently assigned to males that have numerous brothers, cousins, sons and nephews. Lineages with high reproductive success might then contribute disproportionately more individuals with unknown sires to population pedigrees.

However, even substantially increased fieldwork or genotyping might leave parents of some individuals unknown. Specifically, in many systems, immigrants with unknown parents appear following relatively long‐distance dispersal. Immigrants may differ phenotypically from resident natives due to their current or previous environmental experiences (e.g. Pärt 1995; Marr, Keller & Arcese 2002; Kidd *et al*. 2015). More pertinently, any form of local adaptation, genetic divergence among populations due to drift, or non‐random dispersal might create differences in mean genetic values between immigrants and natives within the recipient population. Such effects have been widely documented and underpin the key roles of dispersal in shaping local adaptation (e.g. Postma & van Noordwijk 2005; Garant, Forde & Hendry 2007; Visser 2008) and driving rapid evolution at range margins (e.g. Phillips *et al*. 2008; Travis, Smith & Ranwala 2010). Thus, immigration not only structures the distribution of unknown parents in a pedigree (Gienapp, van Noordwijk & Visser 2013), but may cause individuals with different genetic values to be non‐randomly associated with missing pedigree information. Further, dispersal and hence immigration is commonly sex‐biased, causing different patterns of unknown parents across observed females and males. Overall, we should expect unknown parents, and corresponding missing pedigree information, to be phenotypically and genetically non‐random in wild population studies. Importantly, this non‐randomness will both result from and drive key biological processes.

## Animal models and unknown parents

Before explaining genetic group methods, we first summarize how unknown parents, and consequent missing pedigree information, can affect animal model estimates. We assume basic knowledge of animal models and direct readers elsewhere for introductory guides (Kruuk 2004; Wilson *et al*. 2010) and for further technical details (Lynch & Walsh 1998, ch. 26–27; Mrode 2005).

In brief, an individual's phenotypic value for any polygenic trait is a sum of independent genetic and environmental factors (the ‘infinitesimal model’; Lynch & Walsh 1998, p. 47). Most simply, an individual's phenotypic value deviates from the overall population mean depending on its breeding value (*a*) and residual deviation (*e*) (i.e. *y *= μ + *a *+ *e*, model 1 in Wilson *et al*. 2010). The animal model expresses the phenotype of every individual (**y**) as:

(eqn 1) y=Xβ+Za+e

Factor levels for ‘fixed effects’ are grouped with the overall population mean (or intercept) in vector **β**, where the design matrix **X** maps levels in **β** to corresponding phenotypes in vector **y**. The heart of the animal model is that breeding values (**a**) for each individual included in **y** are estimated as ‘random effects’, where the design matrix **Z** maps each individual's value in **a** to their phenotype in **y**.

Model predictions of **a** potentially allow ecologists to quantify changes in population mean breeding value over time, and hence test for microevolutionary change, and to determine whether selection acts on genetic or environmental components of phenotypic variation (Postma 2006; Gienapp *et al*. 2008; Hadfield *et al*. 2010). Furthermore, animal models directly estimate the variance in true breeding values in the base population (Lynch & Walsh 1998, pp. 78–79; Mrode 2005, ch. 2–3). Such estimates of additive genetic variance can be used to calculate narrow‐sense heritability and predict a trait's potential to evolve in response to selection (Lynch & Walsh 1998, ch. 3; Bijma 2011).

To provide these estimates, animal models rely on the additive genetic relatedness matrix **A** (Glossary, Appendix S1), which quantifies the covariance in additive genetic effects among individuals. In diploid organisms, this covariance is proportional to twice the probability that two individuals inherited homologous alleles ‘identical‐by‐descent’ from common ancestors. The **A** matrix can be constructed from a pedigree following certain rules and assumptions (Wright 1922; Henderson 1976). The information contained in **A** and its structure underpin animal model parameter estimates. Therefore, missing or inaccurate information in **A**, or a structure that does not represent the true additive genetic covariances among individuals in a population, can bias parameter estimates.

In general, relatedness is always defined relative to some reference population (Lynch & Walsh 1998, p. 132; Wang 2014). The animal model **A** matrix reference population is the base population, composed of phantom parents for all individuals in the pedigree that have unknown parents. Phantom parents are assumed to be outbred and unrelated. The covariance among breeding values is $\sigma_{A}^{2}$ **A**, where $\sigma_{A}^{2}$ is the expected additive genetic variance in the base population, which equals the variance in true breeding values of base population individuals (Kruuk 2004; Mrode 2005, ch. 2–3). Because **A** accounts for the probability of inheriting alleles identical‐by‐descent among all pedigreed individuals, under the infinitesimal model **A** also accounts for temporal changes in mean breeding value and $\sigma_{A}^{2}$ (and hence in the distribution of **a**). This property makes animal models very appealing for wild population studies, because **A** can prevent bias in estimates of $\sigma_{A}^{2}$ due to selection, drift, assortative mating and inbreeding (Kennedy, Schaeffer & Sorensen 1988; Mrode 2005, ch. 3; Kruuk 2004; but see Hadfield 2008).

### Consequences of unknown parents

The desirable properties of animal models only hold if **A** is constructed without error from an appropriate reference population. In general, unknown parents can bias animal model parameter estimates because missing pedigree information causes estimates of pairwise relatedness between phenotyped individuals to be biased downward. In the most extreme case where all parents are unknown, the only nonzero elements of **A** would be ones along the leading diagonal. All individuals would be considered unrelated to all others and phenotypic resemblance between individuals that are in fact related could not be correctly attributed to additive genetic effects. More generally, unknown parents can cause additive genetic variance to be underestimated to some degree (Dong, Van Vleck & Wiggans 1988; Cantet *et al*. 2000; but see Morrissey *et al*. 2007). Unknown parents also decrease the ‘accuracy’, or ‘reliability’, of predicted breeding values. All else being equal, accuracy is highest when individuals have numerous close relatives with observed phenotypes (Mrode 2005, pp. 50–52; Postma 2006). Since individuals with unknown parents cannot be associated with phenotypes of parents, or grandparents, or potentially full‐ and half‐siblings, predicted breeding values can be biased such that they more closely resemble an individual's own observed phenotype (plus phenotypes of any descendants; Appendix S3) rather than the true breeding value, thereby reflecting environmental effects on phenotype (Postma 2006). Furthermore, by decreasing the connectedness of relatives across spatially or temporally varying environments, missing pedigree information might also cause genetic effects to be misassigned as environmental effects (Postma 2006). Missing pedigree information therefore erodes the key information that enables animal models to separate genetic and environmental effects.

However, further severe biases can arise when the individuals that are unknown parents are non‐random with respect to additive genetic values. Such biases arise because animal models predict breeding values and estimate additive genetic variances in a base population in which breeding values are assumed to be normally distributed with a mean of zero (Kruuk 2004; Mrode 2005, ch. 3). Meanwhile, the default base population comprises the phantom parents that produced the observed founder population plus the phantom parents of all subsequent individuals in the pedigree with unknown parents. Animal models using pedigrees with numerous unknown parents therefore estimate key parameters based primarily on phantom parents of non‐founders instead of the true base population (Postma 2006). Predictions and estimates from an animal model can therefore be biased if genetic properties differ between the phantom parents of founders vs. non‐founders.

**Figure 2:**
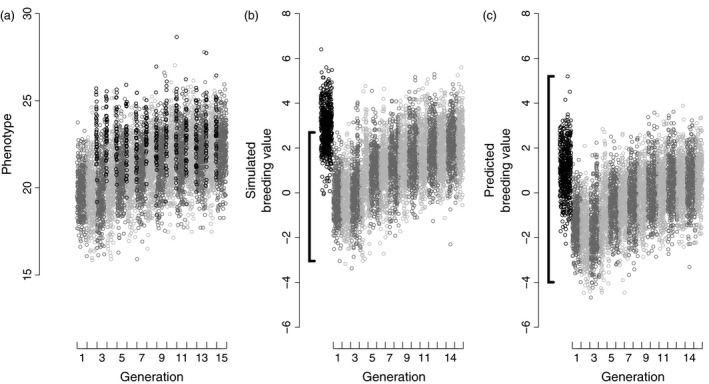
Simulated (a) phenotypes and (b) breeding values across 15 generations, and (c) predicted breeding values from a basic animal model using a pedigree where immigrants have unknown parents. Alternating dark and light grey points distinguish consecutive generations of founders and their descendants. In (a), immigrant phenotypes are plotted in the generation they arrive (black points). In (b) and (c), immigrant simulated and predicted breeding values are plotted to the left of generation one to illustrate that their phantom parents are assigned to the animal model base population. Black brackets demarcate the range and hence variance in breeding values in the (b) founder population and (c) the offspring of the default animal model base population.

To illustrate the problem, we simulated a hypothetical trait for 6000 individuals across 15 generations in a focal population that receives 40 immigrants per generation (details in Appendix S4). Breeding values and environmental deviations, and thus phenotypes, of focal population founders were simulated from normal distributions with means of zero and variances of one. Mates were randomly assigned and offspring breeding values were calculated as the mean of their parents’ breeding values plus a Mendelian sampling deviation. The population was not subject to selection and is large enough to ensure that genetic drift is negligible. No change in mean breeding value or phenotype across generations is therefore expected. However, immigrants from a separate population were simulated with a mean breeding value three units greater (as might arise given local adaptation, drift, or non‐random dispersal), but the variance was also one (i.e. both focal population founders and immigrants have an expected additive genetic variance of one). Therefore, immigrants have greater phenotypic values. Since natives and immigrants were paired with randomly assigned mates, the mean phenotypic value of all individuals within the focal population increased across generations solely due to gene flow stemming from immigration (Fig. 2a).

Since all immigrants have unknown parents, their phantom parents are by default assigned to an animal model base population, along with the phantom parents of the true focal population founders. This combined distribution of breeding values in the default base population is consequently heterogeneous, because the phantom parents of founders and immigrants have different mean values (Fig. 2b,c). Two things happen in an animal model when two or more genetically different groups of individuals are combined into a single base population in this way.

First, a basic animal model predicts breeding values assuming a mean of zero in the default base population (Mrode 2005, p. 40). Consequently, predictions of breeding values regress to the mean breeding value of the default base population and are biased (Van Vleck 1990). In the simulated example, predicted breeding values are biased downwards across all individuals (Fig. 2c). Although predicted breeding values are highly correlated with the true breeding values’ rank order, such bias decreases the accuracy of breeding value prediction, thereby decreasing the ability to predict future phenotypic values.

Secondly, a basic animal model (eqn 1) returns biased estimates of additive genetic variance compared with the expected value in the true base population. The expected additive genetic variance in the true base population can be visualized as the range of true breeding values in the founder population (Fig. 2b). However, an animal model fitted to the simulated data overestimated the additive genetic variance in the founder population by a factor of approximately two. This can be visualized as the range of breeding values across the combined (and hence genetically structured) base population, including immigrants (Fig. 2c). Heritability estimates and predicted microevolutionary responses to selection would consequently be severely biased upwards.

In general, the extent of such bias is hard to predict *a priori* as it depends on the mean and variance of true breeding values in the true base population relative to the means and variances of true breeding values for the different groups in the default base population. However, our simple simulation illustrates that breeding values defined within the context of a single population (Lynch & Walsh 1998, p. 79) are no longer sufficient to represent ‘total additive genetic effects’ when individuals’ genomes comprise mixtures of alleles originating from groups that differ in the mean of their allelic effects. Consequent biases in key quantitative genetic parameter estimates from animal models, resulting from the occurrence of non‐random unknown parents in wild population studies, cannot be ignored. One solution is to use genetic group methods to account for genetic differences between the phantom parents of different types of individuals with unknown parents.

## Genetic groups

**Figure 3:**
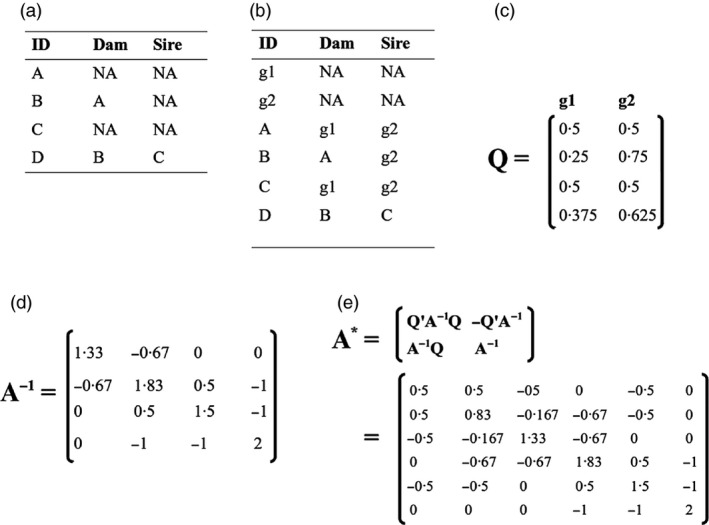
Simple example pedigrees and matrices illustrating (a) a pedigree containing individuals with unknown parents (NA), (b) phantom parents assigned to two genetic groups (g1 and g2), (c) the proportional contributions of each genetic group to each individual's genome, as is used to explicitly model genetic groups as fixed covariate regressions, (d) the inverse relatedness matrix (**A** ^**−1**^) for the pedigree in (a), and (e) the augmented inverse relatedness matrix (A*), used to model genetic group effects implicitly within the random effects.

When unknown parents occur non‐randomly with respect to their additive genetic values for any focal trait, breeding values can differ between non‐founder phantom parents and the true base population. Founders and non‐founders with unknown parents should then be assigned phantom parents from distinct genetic groups (e.g. Fig. 3a,b; Quaas & Pollak 1981; Schaeffer 1991). Animal models that allow mean additive genetic values to differ among the defined groups can then be fitted, thereby reducing bias and directly estimating parameters describing key evolutionary processes such as local adaptation or dispersal. There is no obvious technical reason why genetic group animal models cannot be implemented by evolutionary ecologists as appropriate methods are well established in agricultural sciences (Appendix S5). Here, we explain the basic principles of such models and in Appendix S6 we provide extensive tutorials illustrating how genetic group animal models can be fitted to data.

### Total additive genetic effects

Breeding values (**a**) quantify the average deviation from the population mean genotype attributed to the additive genetic effects of an individual's genotype (Lynch & Walsh 1998, pp. 72–73). In a basic animal model, the default assumption that the base population has a mean breeding value of zero implies the existence of a single genetic group (no genetic structure; Van Vleck 1990). Modelling more than one genetic group allows breeding value predictions to account for genetic structure in the base population.

In an animal model with genetic groups, the ‘total additive genetic effect’ (Appendix S1) of an individual's genotype is separated into a genetic group effect and a breeding value. The genetic group effect measures the expected mean deviation from a reference attributed to a group's total additive genetic effects and the breeding value measures the average deviation from the genetic group mean caused by an individual's genotype (Schaeffer 1991). For example, for phantom parent *i* in genetic group *j*, the total additive genetic effect of its genotype (*u*_*i*_) equals the expected average genetic effect in group *j* (*g*_*j*_) plus a deviation from the group mean caused by *i*'s genotype (its breeding value, *a*_*i*_). Because all breeding values are deviations from group means, all base population breeding values have an expectation of zero. Consequently, breeding values represent standardized measures of additive genetic effects that allow direct comparison across genetic groups of individuals’ additive genetic effects distinct from the mean additive genetic differences among groups.

Each individual inherits the mean of its parents’ genetic group effects plus the mean of its parents’ breeding values, where both are consistent with the probability of inheriting alleles identical‐by‐descent (Appendix S5). The expression for a quantitative trait phenotype y_*i*_ can be rewritten to include genetic group effects:

(eqn 2a) $$y_{i}=\mu +u_{i}+e_{i},$$

which expands to:

(eqn 2b) $$y_{i}=\mu +\sum_{j=1}^{r}q_{ij}g_{j}+a_{i}+e_{i}$$

Here, the total additive genetic effect of individual *i*'s genotype $u_{i}=\sum_{j=1}^{r}q_{ij}g_{j}+a_{i}$ replaces the breeding value *a*_*i*_ in a basic quantitative trait model without genetic groups. The *j*th group effect (*g*_*j*_), out of *r* groups in the base population, contributes to the total additive genetic effects of *i* in proportion to the expected fraction of *i*'s genome derived from group *j* (*q*_*ij*_). Each *g*_*j*_ constitutes an element in the vector **g** containing all genetic group effects. The fraction *q*_*ij*_ can be calculated from *q*_*dj*_ and *q*_*sj*_ of *i*'s parents *d* and *s* (Fig. 3b,c; Appendix S6.2). Therefore, *q*_*ij*_ across all individuals and groups can be calculated from a pedigree, where each *q*_*ij*_ constitutes an element in the matrix **Q** containing all individuals in the pedigree (rows) and all genetic groups in the base population (*r* columns). Each row of **Q**, which lists the contributions of each genetic group to an individual, sums to one.

The collection of eqn 2a for a population can be expressed in vectors and matrices as:

(eqn 3) y=Xβ+Zu+e

The total additive genetic effects (**u**) are normally distributed with an expected covariance of $\sigma_{A}^{2}$ **A** (assuming groups have equal $\sigma_{A}^{2}$). However, the expected mean of **u** is no longer zero, but **Qg**. Therefore, animal models that account for the contribution of genetic group effects to the total additive genetic effects of individuals do so by modelling the mean of **u**.

Mean additive genetic values for each genetic group cannot be uniquely estimated by an animal model, but differences among group means (analogous to anova contrasts) are estimable (Quaas 1988; Hadfield *et al*. 2010; further discussion in Appendix S6.2). Below, we consider the estimation of differences among genetic group means where model estimated genetic group effects are deviations from a reference. In practice, this is often accomplished by specifying an animal model that sets the true founders as the reference group (i.e. assuming the reference group mean effect is zero). This is analogous to the familiar animal model without genetic groups, where the base population is considered a single genetic group (Van Vleck 1990) with an expected breeding value of zero. Genetic group effects are conceptually fixed effects (Glossary, Appendix S1) because they measure the expected mean deviation from the reference in a group's total additive genetic effects (but see Appendices S5 and S6.3.2). In practice, genetic group effects can be fitted and hence estimated within an animal model either ‘explicitly’ as separate fixed effects or `implicitly` as part of the individual total additive genetic effects (i.e. within the random effects structure, Appendix S5). Fitting genetic group effects in either of these two ways will produce equivalent statistical models that yield identical estimates of genetic group effects (Quaas 1988). We fully explain both approaches below.

### Explicit genetic group effects

Genetic group effects represent the differences between the expected mean additive genetic values for each group of phantom parents. Therefore, one obvious approach is to treat genetic group effects as explicit fixed effects within an animal model, thereby estimating differences among group means separately from the deviations from the mean caused by additive genetic effects of individual genotypes (i.e. breeding values). However, because individuals inherit genetic group effects, estimating genetic group effects as separate fixed effects is more complicated than simply fitting categorical fixed effects of discrete group membership. An animal model that fits explicit genetic group effects is:

(eqn 4) y=Xβ+Qg+Za+e

Here, the random effects **a** are breeding values of individuals with expected mean zero and covariance $\sigma_{A}^{2}$ **A** (assuming homogeneous $\sigma_{A}^{2}$ across groups, see Limitations). The standard **A** ^**−1**^ matrix (i.e. matrix inverse of **A**) is utilized as in a basic animal model without genetic groups (e.g. Fig. 3d; Appendix S6.4). To estimate the genetic group effects, the columns of **Q** (e.g. Fig. 3c) are each fitted as separate fixed covariate regressions to obtain estimates of **g** (Quaas 1988). **Q** is obtained directly from the additive genetic relatedness matrix (**A**) as the first *r* columns of the **T** matrix in Henderson's (1976) decomposition **A** = **TDT′**, where **A** includes *r* extra rows and columns for the *r* genetic groups (Robinson 1986; Appendix S6.2).

Solutions to the model in eqn 4 yield *r* regression coefficient estimates in **g** that quantify differences between mean breeding values of each group and the reference, as well as predictions of the individual breeding values in **a**. The total additive genetic effect for any individual (*u*_*i*_) is the sum of the genetic group effects, weighted by the contribution of each genetic group to that individual, plus the individual's breeding value (*a*_*i*_):

(eqn 5) $$u_{i}=\sum_{j=1}^{r}q_{ij}g_{j}+a_{i}$$

### Implicit genetic group effects

As an alternative animal model to one explicitly estimating genetic group effects separately from individual breeding values, the model in eqn 3 can be fitted to directly predict each individual's total additive genetic effects (**u**) (Quaas & Pollak 1981; Appendix S5). This is possible because genetic group effects (**g**) are inherited the same way as breeding values (**a**), as quantified by the **A** matrix. Therefore, the sum of their effects can be modelled by augmenting **A** ^**−1**^ to implicitly incorporate the group effects into predictions of **u** (Fig. 3d,e; Appendix S6.3). The augmented matrix A* is constructed directly from a pedigree following the rules used to construct **A** ^**−1**^ (Quaas 1988; Westell, Quaas & Van Vleck 1988). The vector of random effects **u** contains each individual's total additive genetic effects and are assumed to be normally distributed with mean equal to **Qg** and variance $\sigma_{A}^{2}$ **A** (assuming homogeneous $\sigma_{A}^{2}$ across groups, see Limitations).

Solutions to the model in eqn 3 return predictions of **u**, the predicted total additive genetic effects for each individual (*u*_*i*_), and estimates of the *r* group effects **g**. In contrast to the approach where genetic group effects are explicitly estimated as separate fixed regression coefficients, obtaining the predicted breeding value for an individual (*a*_*i*_) requires subtracting the sum of the genetic group effects, weighted by the contribution of each genetic group to that individual, from the predicted total genetic effects of the individual (*u*_*i*_) returned by the model:

(eqn 6) $$a_{i}=u_{i}-\sum_{j=1}^{r}q_{ij}g_{j}$$

### Fitting genetic groups: eliminating bias and estimating new parameters

**Figure 4:**
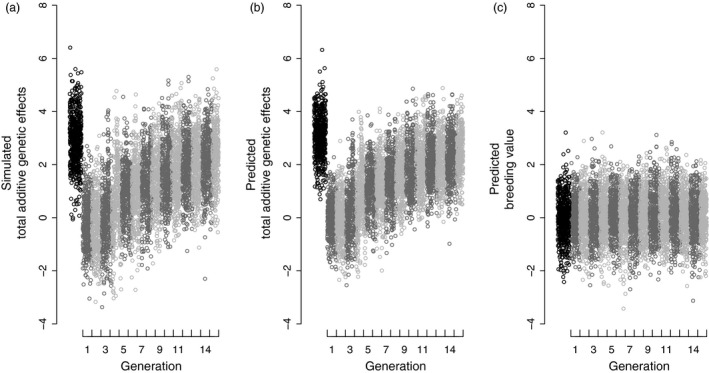
(a) Simulated total additive genetic effects and predicted (b) total additive genetic effects and (c) breeding values from an animal model fitting genetic group effects. Alternating dark and light grey points distinguish consecutive generations of founders and their descendants. Simulated data correspond to the phenotypes in Fig. 2a. Immigrant values (black points) are plotted to the left of generation one to illustrate that their phantom parents are assigned to the animal model base population.

Armed with a conceptual understanding of genetic group animal model methods, the benefit of fitting such models can be illustrated by returning to the simple simulation depicted in Fig. 2. Here, the default base population includes phantom parents of both founders and immigrants and is consequently genetically structured (Fig. 2b). Although only 15% of simulated individuals have unknown parents, which is lower than in most wild population studies (Fig. 1, Appendix S2), a basic animal model returns substantially biased predictions of breeding values and a biased estimate of additive genetic variance. The biased breeding value prediction reflects a regression to the mean breeding value in the combined base population, while the additive genetic variance is overestimated because the total additive genetic effects of immigrants fall outside the range of true breeding values in the founders. To resolve these problems, instead of ignoring genetic structure in the combined base population, we can define two genetic groups and fit a genetic group animal model to directly estimate differences between expected group mean additive genetic effects in the base population (**g**). The genetic group effects can be fitted either explicitly by including fixed covariate regressions on columns of **Q**, or implicitly by modelling total additive genetic effects using A*. Both models provide equivalent unbiased predictions of breeding values and individual total additive genetic effects as well as unbiased estimates of the additive genetic variance for the simulated data set (Fig. 4). Furthermore, such models recover the simulated difference of three units between the mean total additive genetic effects of the founders and immigrants (Fig. 4a,b), therefore directly estimating the difference between the two populations in additive genetic value for the hypothetical trait.

Fitting genetic group models can also illuminate interesting biology underlying apparent temporal trends in additive genetic values, by distinguishing trends in breeding values from trends in total additive genetic effects. For example, given an observed temporal trend in mean phenotype (Fig. 2a), we might wish to test for an underlying trend in mean breeding value (Hadfield *et al*. 2010). An animal model without genetic groups would predict breeding values that change over time (Fig. 2c). However, there is no selection (or drift) in our simulation that could produce such trends. Indeed, an animal model with genetic groups predicts breeding values that do not show any temporal trend (Fig. 4c). The predicted total additive genetic effects (Fig. 4b) do increase over time, causing the increasing phenotype (Fig. 2a). However, phenotypic change reflects the increasing contribution of alleles originating from the genetically larger immigrant population. The change in the population‐wide proportion of alleles derived from immigrants is quantified by **Q**. Figs 2a and 4b,c therefore demonstrate local evolution resulting from gene flow, not from local selection. It is not yet clear how such immigrant effects might alter interpretations of past analyses of genetic trends in wild populations (e.g. Hadfield *et al*. 2010; Teplitsky *et al*. 2010). However, genetic group animal models clearly offer exciting opportunities to quantify microevolution occurring by both local selection and gene flow.

## How to fit genetic group animal models

While the complexities of fitting genetic group animal models might seem intimidating, in fact there is no technical reason why such models cannot be fitted to appropriate wild population data using either residual maximum likelihood or Bayesian methods. Further, genetic group methods can be applied to multivariate and non‐Gaussian response variables. In Appendix S6.4, we provide extensive tutorials that demonstrate how to fit genetic groups either explicitly as separate fixed regressions or implicitly within the random effects structures that predict total additive genetic effects in animal models implemented in MCMCglmm (Hadfield 2010) and asreml in the r program (Butler *et al*. 2009; R Core Team 2015) and the standalone programs WOMBAT (Meyer 2007) and ASReml (Gilmour *et al*. 2014). Because there were previously either no or limited capabilities to implement such models (Table S6.1), we have written generic functions to calculate the **Q** and A* matrices (available in the r package nadiv, version ≥2.14.2; Wolak 2012; http://github.com/matthewwolak/nadiv), thereby extending and standardizing current software capabilities. We comprehensively demonstrate how to use these functions with MCMCglmm, asreml, WOMBAT and ASReml (Appendix S6.4) and model outputs have been deposited in the Dryad Digital Repository: http://dx.doi.org/10.5061/dryad.jf7cr (Wolak & Reid 2016b). The simulated data plotted in Figs 2 and 4 are provided and underlie the tutorials. The r code to generate such data is also provided and can simulate populations with genetic groups and different phenotypic, genetic and environmental trends, thereby extending available simulation tools with which to investigate the evolutionary ecology of quantitative traits (Appendix S4).

However, as with any complex quantitative genetic analyses of wild population data, key decisions need to be carefully made before animal models with genetic groups can be fitted. Further, limitations of and constraints on the genetic group methods remain, meaning that estimated effects will need to be interpreted with due caution. In the following sections, we summarize some key decisions and suggest potential resolutions to current limitations.

### Which approach: explicit or implicit?

Fitting genetic groups explicitly as separate fixed regressions or implicitly through random effects of individual total additive genetic value yields equivalent models. These two approaches, therefore, yield identical estimates of genetic group effects (Quaas 1988). Although the predicted values associated with the random variable identifying the additive genetic effects will differ between the two approaches (i.e. **a** vs. **u**), values are easily transformed from one to the other through simple mathematical formulae (eqns 5 and 6). However, there are subtle differences that may render either the explicit or implicit approach more suitable for any particular analysis or data set.

Modelling genetic group effects implicitly within the total additive genetic effects (**u**) means that the uncertainty in genetic group effects is included in the prediction of **u**, which can increase the prediction error variance of **u**. Unless uncertainty is incorporated into calculations transforming values from **u** and **a**, the accuracy of calculated breeding values (via eqn 6) is lower than predicted breeding values from the explicit genetic group approach (Kennedy 1981). Conversely, uncertainty in estimated genetic group effects fitted explicitly as separate fixed regressions is not directly included in the prediction of the individual breeding values (but see Appendix S6.4.2.2). However, frequentist statistical hypothesis tests for differences among genetic group effects are perhaps most straightforward using the explicit fixed regression method and Wald tests (e.g. Wilson *et al*. 2010). Indeed, the explicit fixed regression approach might generally prove easiest to implement, particularly in multivariate models where different groups are defined for different traits (e.g. Misztal *et al*. 2013). However, the ease of implementation only occurs if the genetic group covariates are not confounded with other modelled fixed effects. Conversely, fitting genetic group effects implicitly within the random effects may actually reduce computational requirements, particularly when many groups are defined. This occurs when the sections of A* pertaining to genetic groups contain more zero elements than the columns of **Q** used in the alternative fixed regressions, thus capitalizing on efficient sparse matrix algorithms. Further, implicit genetic group models can be extended to account for among group structure in maternal genetic effects, since direct and maternal additive genetic effects can be assigned to different genetic groups (Van Vleck 1990; Cantet *et al*. 2000). However, the implicit approach cannot currently be recommended in all software programs (Table S6.1), because of issues arising within the linear algebra operations, although these issues can sometimes be mitigated (Appendices S6.3.1 and S6.3.2).

The best approach may also depend on the chosen method of statistical inference. With Bayesian inference, appropriate prior distributions need to be specified. Appropriate priors for genetic group effects fitted explicitly as separate fixed regressions can be specified, and prior sensitivity assessed, in a relatively straightforward manner (Appendix S6.4.2.2). However, it is less clear how priors specified for additive genetic variances affect posterior inference on genetic group effects fitted implicitly (Appendix S6.4.2.3), particularly when variance component estimation is the aim of the analysis. Consequently, fitting genetic groups explicitly as separate fixed regressions may be the most straightforward approach when using Bayesian inference (but see Gara, Rekik & Bouallègue 2006). Overall, the most practical approach to fitting genetic group effects will depend on a combination of question, data set, model structure and statistical paradigm.

### How many groups?

Obvious key decisions concern how many genetic groups to define and which phantom parents to include in each. Since clear general rules for defining genetic groups do not exist, a sensible approach is to define biologically motivated groups and test sensitivity by fitting models with different groupings. The maximum number of genetic groups that can be fitted will be constrained, since model complexity will rapidly increase and fitting numerous groups might cause model terms to be confounded. For example, genetic groups defined to comprise individuals with unknown parents in single years may become confounded with year effects modelled to capture environmental variation. Modelling too many groups can also generate non‐unique solutions for group effects (Schaeffer 1991) and cause model convergence failure (Appendices S6.3.1 and S6.3.2). Sex‐specific selection, age at sexual maturity and/or dispersal may necessitate defining separate genetic groups for phantom dams and sires to correctly model sex‐specific breeding value distributions (Westell, Quaas & Van Vleck 1988) resulting from sex‐specific genetic structure in the base population (Wolak, Roff & Fairbairn 2015). However, purely sex‐specific genetic group effects might be confounded with standard fixed effects of sex. In general, the degree to which genetic group effects and other terms are confounded will depend on the connectedness of the pedigree across levels of other model terms (Kennedy & Trus 1993).

Adding unnecessary genetic groups increases the error variance of predicted total additive genetic values, but does not itself bias predictions (Famula 1981). However, it is not yet clear how modelling numerous genetic groups might affect variance component estimation, especially in the context of wild population pedigree structures. Moreover, in multivariate analyses different genetic group structures might ideally need to be modelled for different traits (Misztal *et al*. 2013). Considerable care is therefore warranted, while simulation studies and transparently reporting the sensitivity of analyses to grouping strategies are necessary to generate useful rules of thumb.

Concerns over how best to define genetic groups may be alleviated by fuzzy classification (Fikse 2009), where phantom parents are assigned to multiple genetic groups with accompanying probabilities of group membership. Fuzzy classification can reduce the number of groups that need to be modelled, thereby improving accuracy and reducing confounding (Fikse 2009). This approach might be particularly useful for defining genetic groups when temporal trends in breeding values are hypothesized, although fuzzy classification does not by itself quantify temporal trends. Appendix S7 discusses strategies for fuzzy classification and demonstrates how to incorporate such classifications into the **Q** and A* matrices constructed using the nadiv package.

Alternatively, Schaeffer (1991) proposed creating a unique phantom parent identity for each unknown parent and for each phantom ancestor (e.g. each phantom individual's own parents) spanning every generation back to the founder population. A modified A* is then constructed which includes variation among individuals in the number of generations from the base population. This method could provide an alternative to defining genetic groups when individuals with unknown parents occur at different times in longitudinal studies. However, the age of individuals with unknown parents and the generation time need to be known. Although the algorithm to construct such a modified A* is available (Schaeffer 1991), no implementations or methodological assessments have been published.

Defining genetic groups to balance the number of groups vs. the number of phantom parents assigned to each group is not likely to greatly affect an animal model's ability to estimate genetic group effects with acceptable precision. This is because estimating deviations among mean genetic group additive genetic effects requires less data than estimating the variance of random effects. Further, all descendants of individuals with phantom parents contribute to the estimated genetic group effects, not just the base individuals assigned to each group. Although a given genetic group's contribution to an individual's total additive genetic effects decreases with mating outside the group by 1/2^*n*^, over *n* generations, mating between individuals with ancestors from the same group will increase that group's contribution to the population. Consequently, genetic groups defined in a base population may contribute substantial proportions of total additive genetic effects even after many generations (e.g. Wolak & Reid 2016a). Therefore, the number of base population individuals assigned to any one genetic group does not have to be large as long as those individuals contribute descendants to the pedigree.

### Limitations

Despite their potential utility, current genetic group methods have limitations. One key limitation is that the magnitude of additive genetic variance is assumed to be homogeneous across groups. This assumption allows the covariance among relatives to be modelled with a single additive genetic variance for the entire population (as in our simulations, Appendix S4). However, evolutionary dynamics can cause the variance in true total additive genetic values within genetic groups to differ among groups, violating the assumption of homogeneous variances (Alfonso & Estany 1999). How this assumption will impact breeding value prediction and variance component estimation using wild population data sets is currently unknown. However, it means that current models cannot explicitly quantify spatial and temporal variation in additive genetic variance within and among populations, which is itself of major biological interest. García‐Cortés & Toro (2006) proposed a method to estimate heterogeneous additive genetic variances across genetic groups by incorporating into the covariances among relatives the change in additive genetic variance due to segregational variance arising when alleles originating from different groups are mixed. However, estimating separate additive genetic variances for just two genetic groups approximately triples the number of equations to be solved (García‐Cortés & Toro 2006). Such models may therefore impose unrealistic demands on wild population data sets.

Traditional genetic group models (Appendix S5) assume that phantom parents are unrelated within and across groups and that no drift or inbreeding occurs within the base population (Legarra *et al*. 2015). However, some phantom parents will commonly be related, particularly when the cause of unknown parentage is incomplete sampling (Misztal *et al*. 2013). Legarra *et al*. (2015) proposed a general framework for constructing relatedness matrices that allows base population individuals to be inbred and related and allows for heterogeneous additive genetic variances across groups. This method provides a particularly promising avenue as it can incorporate both pedigree and genomic information and future work should examine its suitability for estimating additive genetic variances in wild populations.

## Conclusion

Wild population pedigrees almost always contain incomplete individual pedigrees, and the unknown parents are likely to be non‐random with respect to additive genetic values for traits of interest. We highlight that, in populations where the mean additive genetic values of founder individuals differ from those of other individuals with unknown parents, animal model parameter estimates can be substantially biased. Fortunately, available genetic group methods can serve to minimize such bias (given appropriate data sets and implementation). We propose that such methods should be applied in animal model analyses of wild population data and provide r functions, examples and tutorials to facilitate implementation (Appendices S4, S6, and S7). Since the consequences of missing pedigree information are likely to differ from the consequences of missing phenotypic information (Hadfield 2008; Nakagawa & Freckleton 2008), it remains an open question as to what degree missing pedigree information biases quantitative genetic parameter estimates generated by basic animal models fitted to wild population data (but see Morrissey *et al*. 2007). To answer this question, researchers with diverse data sets will need to fit appropriate genetic group models such that we can collectively quantify such biases, identify when the greatest problems occur and devise protocols for resolving emerging issues.

Genetic group methods are not a panacea; they cannot be expected to completely rescue analyses based on poor population pedigrees or fix all resulting problems. Missing pedigree information may still bias animal model parameter estimates even when genetic groups are modelled. Unknown parents decrease pedigree connectedness, potentially causing phenotypic variation to be attributed to environmental rather than genetic effects. Likewise, missing parent information can affect estimates of inbreeding and hence inbreeding depression (Pemberton 2008). Such biases might be minimized by using ‘pedigree‐free’ animal models which use realized relatedness estimated directly from high‐density genetic marker data instead of expected relatedness derived from a pedigree (Garant & Kruuk 2005; Pemberton 2008; Speed & Balding 2015). However, unknown parents still fundamentally alter relationships (in addition to relatedness) and might consequently bias estimated environmental and social effects. For example, if sire identities are consistently hard to observe, then full‐siblings are defined as maternal half‐siblings, and paternal half‐siblings are defined as unrelated. Any paternal environmental effects might then confound estimates of genetic effects. Bias might be reduced by reconstructing relationships from marker data (Wang 2004), or observing intact broods, clutches or litters (Coltman 2005; Husby *et al*. 2010; Kim *et al*. 2013), demonstrating that some degree of individual pedigree data may still be required.

It is equally important to realize the opportunities for conceptual advances that genetic group animal models afford, rather than solely viewing them as technical means to minimize bias. Genetic group methods can reveal the relative additive genetic values of natives and immigrants for any quantitative trait of interest, and thereby distinguish microevolutionary changes in population mean phenotypes arising through direct responses to local selection from changes caused by immigration and resulting gene flow. We can thereby quantify the degree to which immigrants introduce genetic effects that are congruent or counter to the direction of local selection, which is an essential step towards predicting adaptive responses to environmental change and explaining microevolutionary stasis (Merilä, Sheldon & Kruuk 2001; Garant, Forde & Hendry 2007; Visser 2008).

## Data accessibility

Data associated with this paper are deposited in the Dryad Digital Repository http://dx.doi.org/10.5061/dryad.jf7cr (Wolak & Reid 2016b).

## Supporting information


**Appendix S1.** Glossary.
**Appendix S2.** Collection of pedigrees from wild populations.
**Appendix S3.** Breeding value prediction.
**Appendix S4.** Simulating data with genetic group effects.
**Appendix S5.** Development of genetic groups in agricultural science.
**Appendix S6.** Construction and implementation of **Q** and A*.
**Appendix S7.** Fuzzy classification of genetic groups.
