# Supplementary material for: Accounting for genetic differences among unknown parents in microevolutionary studies: how to include genetic groups in quantitative genetic animal models
Source: J Anim Ecol. 2016 Nov 3;86(1):7–20. doi: 10.1111/1365-2656.12597 (PMC5217070; doi:10.1111/1365-2656.12597)
Supplement: Supplementary file 1 — Appendix S1. Glossary. Appendix S2. Collection of pedigrees from wild populations. Appendix S3. Breeding value prediction. Appendix S4. Simulating data with genetic group effects. Appendix S5. Development of genetic groups in agricultural science. Appendix S6. Construction and implementation of Q and A*. Appendix S7. Fuzzy classification of genetic groups. [file JANE-86-7-s001.pdf]

Supporting Information to

Accounting for genetic differences among unknown parents in microevolutionary studies: How to include genetic groups in quantitative genetic animal models

Matthew E. Wolak & Jane M. Reid

Contents

Appendix S1: Glossary **3**

References: Appendix S1 . . . 5

Appendix S2: Collection of pedigrees from wild populations **6**

Table S2.1 References . . . 10

References: Appendix S2 . . . 12

Appendix S3: Breeding value prediction **13**

References: Appendix S3 . . . 14

Appendix S4: Simulating data with genetic group effects **15**

4.1 Simulating a fixed difference between groups . . . 16

4.2 Simulating a temporal trend in breeding values . . . 17

Appendix S5: Development of genetic groups in agricultural science **19**

References: Appendix S5 . . . 21

Appendix S6: Construction and implementation of Q and A* **23**

6.1 A small example pedigree . . . 25

6.2 Constructing Q . . . 26

6.3 Constructing A* . . . 28

6.3.1 Genetic groups on top or bottom of A*? . . . 29

6.3.2 Removing singularities and other problems within A* . . . 31

6.4 How to fit genetic groups in MCMCglmm, ASReml-R, ASReml-standalone, & WOMBAT . . . 33

6.4.1 Tutorial dataset `ggTutorial` . . . 33

6.4.2 `MCMCglmm` . . . 35

6.4.2.1 Preparing a pedigree with genetic groups . . . 36

6.4.2.2 Fixed explicit genetic group effects with Q (from `nadiv`) . . . 37

6.4.2.3 Random implicit genetic group effects with A* (from `nadiv`) 40
6.4.3 `ASReml` in `R` 43
6.4.3.1 Preparing a pedigree with genetic groups 43
6.4.3.2 Fixed explicit genetic group effects with Q (from `nadiv`) 45
6.4.3.3 Fixed implicit genetic group effects with A* (from `nadiv` or `asreml`) 47
6.4.3.4 Random implicit genetic group effects with A* (from `nadiv`) 50
6.4.4 ASReml Standalone 53
6.4.4.1 Preparing a pedigree with genetic groups 54
6.4.4.2 Fixed explicit genetic group effects with Q (from `nadiv`) 55
6.4.4.3 Fixed implicit genetic group effects with A* (from `nadiv`) 58
6.4.4.4 Fixed implicit genetic group effects with A* (from ASReml) 63
6.4.4.5 Random implicit genetic group effects with A* (from ASReml) 66
6.4.5 WOMBAT 70
6.4.5.1 Preparing a pedigree with genetic groups 71
6.4.5.2 Fixed explicit genetic group effects with Q (from `nadiv`) 72
6.4.5.3 Random implicit genetic group effects with A* (from `nadiv`) 75
6.4.5.4 WOMBAT's genetic groups (Random group effects with Q from `nadiv`) 79
References: Appendix S6 82

Appendix S7: Fuzzy classification of genetic groups **85**
7.1 Constructing the fuzzy classification matrix F 86
7.2 Constructing $\mathbf{Q_f}$ 87
7.3 Constructing $\mathbf{A^*_f}$ 89
References: Appendix S7 90

Software version information **90**

Appendix S1: Glossary

We define a list of key words and concepts that are used in the main text.

accuracy (reliability): The correlation between true and predicted breeding values, where the reliability is the accuracy squared (Mrode 2005, p.50). True breeding values are the actual additive genetic effects of individuals. Predicted breeding values are the breeding values assigned by a statistical model.

additive genetic relatedness matrix (A): Also called the numerator relationship matrix. A symmetrical matrix (same values above and below the diagonal) that indicates the proportion of homologous alleles shared **identical-by-descent** among all individuals in a pedigree. The matrix has diagonal elements equal to $1 + f_i$, where f_i is individual i's coefficient of inbreeding, which measures the probability that homologous alleles in two gametes drawn at random from individual i will be identical-by-descent relative to the pedigree base population (Lynch & Walsh 1998, p.135; Wang 2014). The off-diagonal elements are the numerators in Wright's coefficient of relationship between two individuals (1922), which measures twice the coefficient of coancestry, or kinship, and is defined as the probability that two homologous alleles drawn at random from two individuals will be identical-by-descent relative to the pedigree base population (Lynch & Walsh 1998, p.135; Wang 2014).

base population: The **phantom parents** of individuals in the pedigree with unknown parents. Basic animal models assume that the base population has a mean breeding value of zero and all subsequent breeding values are expressed relative to the breeding values of the base population. Elsewhere, base populations are alternatively defined for convenience as those individuals in the pedigree with unknown parents (Kruuk 2004; Mrode 2005, p.62).

breeding value: The sum of additive allelic effects for an individual's genotype defined with respect to a particular population and expressed as a deviation from the reference population's mean genotypic value (Lynch & Walsh 1998, p.79).

explicit genetic groups: A method of analysis which estimates the differences among genetic groups in their mean total additive genetic effects by fitting a separate **fixed effect** regression for each genetic group. This approach results in a statistical model that is equivalent to the model under the **implicit genetic group** approach.

fixed effects: Factors for which an estimate of the unknown effect size of each level is of interest and for which all levels in the population are assumed to have been observed (Searle 1997; Bolker et al. 2009).

founder population: The first cohort of observed individuals in a study, for which all parents are by

definition unknown.

genetic group: A subset of the pedigree **base population** that can be genetically distinct from the rest of the base population. The genetic group effect is the mean deviation of total additive genetic effects in a particular genetic group. Conceptually, the genetic group effects to be estimated can be thought of as **fixed effects**.

identical-by-descent (IBD): Alleles that are direct descendants of the same allele in a common ancestor (Lynch & Walsh 1998, p.132).

implicit genetic groups: A method of analysis which directly models the **total additive genetic effects** of individuals (as opposed to modelling their **genetic group effects** separately from their **breeding values**), thereby incorporating the genetic group effects into the individual **random effects**. Estimates of the fixed genetic group effects are also provided by the model. This approach results in a statistical model that is equivalent to the model under the **explicit genetic group** approach.

infinitesimal model: A genetic model of quantitative traits that assumes phenotypes are determined by an infinite number of loci, each with an infinitesimally small effect on trait expression (Fisher 1918; Lynch & Walsh 1998, p.47; Mrode 2005, p.2).

phantom parent: An unknown (i.e., unidentified or unobserved) parent of an individual. Analyses that make genetic inferences from pedigrees typically assume that phantom parents have one offspring and have the same genetic properties as the pedigree **base population** (Westell, Quaas & Van Vleck 1988).

random effects: Factors for which the levels are a sample from the entire population of values constituting the random variable (Searle 1997). For a given dataset with a sample of the observed dependent variable, random effects are the realized (but unobservable) values of the random variable. Predicting the realized values of the random effects and estimating the parameters describing the distribution of levels are the main aims in fitting mixed effect genetic models (Searle 1997; Bolker et al. 2009).

relatedness: A measure of the extent to which individuals share alleles **identical-by-descent** that is defined relative to the **base population**. Relatedness has many formal definitions (see Lynch & Walsh 1998, p.132), but is used here to refer to elements of the **additive genetic relatedness matrix** (which equal twice the coefficient of coancestry).

total additive genetic effects: The sum of the additive effects of an individual's genes on any focal phenotype. The expected value is the mean of the additive genetic effects that an individual receives from both parents.

References: Appendix S1

Bolker, B.M., Brooks, M.E., Clark, C.J., Geange, S.W., Poulsen, J.R., Stevens, M.H.H. & White, J.S. (2009) Generalized linear mixed models: a practical guide for ecology and evolution. Trends in Ecology and Evolution, 24, 127–135.

Fisher, R.A. (1918) The correlation between relatives on the supposition of Mendelian inheritance. Transactions of the Royal Society of Edinburgh, 52, 399–433.

Kruuk, L.E.B. (2004) Estimating genetic parameters in natural populations using the "animal model." Philosophical Transactions of the Royal Society B, 359, 873–890.

Lynch, M. & Walsh, B. (1998) Genetics and Analysis of Quantitative Traits. Sinauer Associates, Incorporated, Sunderland, MA.

Mrode, R.A. (2005) Linear Models for the Prediction of Animal Breeding Values, 2nd ed. CABI Publishing, Cambridge, MA.

Searle, S.R. (1997) The matrix handling of BLUE and BLUP in the mixed linear model. Linear Algebra and its Applications, 264, 291–311.

Wang, J. (2014) Marker-based estimates of relatedness and inbreeding coefficients: an assessment of current methods. Journal of Evolutionary Biology, 27, 518–530.

Westell, R.A., Quaas, R.L. & Van Vleck, L.D. (1988) Genetic groups in an animal model. Journal of Dairy Science, 71, 1310–1318.

Wright, S. (1922) Coefficients of inbreeding and relationship. American Naturalist, 56, 330–338.

Appendix S2: Collection of pedigrees from wild populations

To illustrate that substantial numbers of parents are commonly unknown in wild population pedigrees, we extracted standard pedigree statistics from published papers citing the **R** package **pedantics** (Morrissey & Wilson 2010). The focal statistics comprised the total number of individual animals in the pedigree (i.e., pedigree size), the percentages of individuals that had unknown dams and sires [calculated as: 100 * (pedigree size - the number of identified maternities or paternities) / pedigree size], and maximum pedigree depth (i.e., the maximum number of lineal ancestors of any individual). Only pedigrees from wild populations were retained; pedigrees from captive populations were excluded (e.g., zebra finch, *Taeniopygia guttata*, located in Seewiesen, Germany). Use in quantitative genetic analyses was not a requirement for inclusion of a pedigree in our summary. When data for both a full pedigree and a pruned pedigree were available in the same paper, we report the largest pedigree for which the required statistics were provided. Pedigree statistics from multiple papers using the same study population are included since reported pedigree structures often differed due to further data collection, paternity assignment, or different degrees of pruning to informative individuals for different analyses.

Table S2.1. Standard pedigree statistics from published papers reporting values from `pedantics` that indicate the total number of individual animals in the pedigree, the percentages of individuals that had unknown dams and sires, and the maximum pedigree depth. Bird species are ordered following the British List (BOU 2013). The order from left to right of Fig. 1 in the main text corresponds to the order of rows here.

Organism	Common name	Population	Pedigree size	%Unknown dams	%Unknown sires	Maximum pedigree depth	Ref.
**Birds**							
Hydrobates pelagicus	European storm petrel	Benidorm Island, Spain	230	67	69	2	[12]
Falco tinnunculus	Common kestrel	Campo Azalvaro, Spain	2716	27	27	7	[11]
Charadrius melodus	Piping plover	Great Lakes, USA	2420	6	6	11	[20]
Cyanistes caeruleus	Blue tit	Gotland, Sweden	1090	20	20	4	[5]
Cyanistes caeruleus	Blue tit	Wytham Woods, UK	23023	14	15		[14]
Parus major	Great tit	Boshoek, Belgium	2178	44	46	12	[13]
Parus major	Great tit	Hoge Veluwe, The Netherlands	3460	67	67	16	[9]
Parus major	Great tit	Oosterhout, The Netherlands	1062	45	47	18	[9]
Parus major	Great tit	Vlieland,	3686	28	29	40	[9]

		The Netherlands					
Parus major	Great tit	Lauwersmeer, The Netherlands	2565	50	50	15	[19]
Parus major	Great tit	Bagley Wood, UK	8144	17	17		[7]
Parus major	Great tit	Bagley Wood, UK	1635	67	68		[6]
Parus major	Great tit	Wytham Woods, UK	156305	53	53		[14]
Hirundo rustica	Barn swallow	Denmark	487	70	67	3	[23]
Hirundo rustica	Barn swallow	Spain	1407	87	88	4	[23]
Acrocephalus arundinaceus	Great reed warbler	Lake Kvismaren Sweden	557	62	62	8	[21]
Acrocephalus arundinaceus	Great reed warbler	Lake Kvismaren, Sweden	550	62	61	7	[22]
Cinclus cinclus	White-throated dipper	Zurich, Switzerland	380	23	23	14	[1]
Ficedula albicollis	Collared flycatcher	Gotland, Sweden	7246	72	73	11	[10]
Ficedula hypoleuca	Pied flycatcher	Tomsk Region, Western Siberia	413	61	59	6	[4]
Melospiza melodia	Song sparrow	Mandarte Island, Canada	5721	3	3	26	[26]
**Mammals**							
Cervus elaphus	Red deer	Isle of Rum,	1336	11	51		[25]

		Scotland					
Cervus elaphus	Red deer	Isle of Rum, Scotland	452	16	52		[16]
Ovis aries	Soay sheep	St. Kilda, Scotland	7299	43	71	8	[17]
Ovis aries	Soay sheep	St. Kilda, Scotland	1752	23	39	9	[8]
Ovis aries	Soay sheep	St. Kilda, Scotland	6740	11	32	10	[2]
Mungos mungo	Banded mongoose	Queen Elizabeth National Park, Uganda	1534	41	59		[18]
Tamiasciurus hudsonicus	North American red squirrel	SW Yukon, Canada	7799	21	77		[15]
Macaca mulatta	Rhesus macaque	Cayo Santiago Island, Puerto Rico	428	3	63	8	[3]
Papio cynocephalus	Savannah baboon	Amboseli, Kenya	1409	8	63		[24]

Table S2.1 References

[1] Becker, P.J.J., Reichert, S., Zahn, S., Hegelbach, J., Massemin, S., Keller, L.F., Postma, E. & Criscuolo, F. (2015) Mother–offspring and nest-mate resemblance but no heritability in early-life telomere length in white-throated dippers. Proceedings of the Royal Society B, 282, 20142924.

[2] Bérénos, C., Ellis, P.A., Pilkington, J.G. & Pemberton, J.M. (2014) Estimating quantitative genetic parameters in wild populations: a comparison of pedigree and genomic approaches. Molecular Ecology, 23, 3434–3451.

[3] Blomquist, G.E. & Brent, L.J.N. (2014) Applying quantitative genetic methods to primate social behavior. International Journal of Primatology, 35, 108–128.

[4] Bushuev, A. V, Husby, A., Sternberg, H. & Grinkov, V.G. (2012) Quantitative genetics of basal metabolic rate and body mass in free-living pied flycatchers. Journal of Zoology, 288, 245–251.

[5] Drobniak, S.M., Dubiec, A., Gustafsson, L. & Cichoń, M. (2015) Maternal age-related depletion of offspring genetic variance in immune response to phytohaemagglutinin in the blue tit (Cyanistes caeruleus). Evolutionary Biology, 42, 88–98.

[6] Evans, S.R., Schielzeth, H., Forstmeier, W., Sheldon, B.C. & Husby, A. (2014) Nonautosomal genetic variation in carotenoid coloration. The American naturalist, 184, 374–383.

[7] Evans, S.R. & Sheldon, B.C. (2012) Quantitative genetics of a carotenoid-based color: heritability and persistent natal environmental effects in the great tit. The American Naturalist, 179, 79–94.

[8] Hayward, A.D., Garnier, R., Watt, K. a, Pilkington, J.G., Grenfell, B.T., Matthews, J.B., Pemberton, J.M., Nussey, D.H. & Graham, A.L. (2014) Heritable, heterogeneous, and costly resistance of sheep against nematodes and potential feedbacks to epidemiological dynamics. The American Naturalist, 184, S58–76.

[9] Husby, A., Hille, S.M. & Visser, M.E. (2011) Testing mechanisms of Bergmann's rule: phenotypic decline but no genetic change in body size in three passerine bird populations. American Naturalist, 178, 202–213.

[10] Husby, A., Schielzeth, H., Forstmeier, W., Gustafsson, L. & Qvarnstrom, A. (2013) Sex chromosome linked genetic variance and the evolution of sexual dimorphism of quantitative traits. Evolution, 67, 609–619.

[11] Kim, S.-Y., Fargallo, J.A., Vergara, P. & Martinez-Padilla, J. (2013) Multivariate heredity of melanin-based coloration, body mass and immunity. Heredity, 111, 139–146.

[12] Kim, S.-Y., Sanz-Aguilar, A., Mínguez, E. & Oro, D. (2012) Small-scale spatial variation in evolvability for life-history traits in the storm petrel. Biological Journal of the Linnean Society, 106, 439–446.

[13] Korsten, P., van Overveld, T., Adriaensen, F. & Matthysen, E. (2013) Genetic integration of local dispersal and exploratory behaviour in a wild bird. Nature Communications, 4, 2362.

[14] Liedvogel, M., Cornwallis, C.K. & Sheldon, B.C. (2012) Integrating candidate gene and quantitative genetic approaches to understand variation in timing of breeding in wild tit populations. Journal of Evolutionary Biology, 25, 813–823.

[15] Mcfarlane, S.E., Gorrell, J.C., Coltman, D.W., Humphries, M.M., Boutin, S. & McAdam, A.G. (2014) Very low levels of direct additive genetic variance in fitness and fitness components in a red squirrel population. Ecology and Evolution, 4, 1729–1738.

[16] Morrissey, M.B., Walling, C.A., Wilson, A.J., Pemberton, J.M., Clutton-Brock, T.H. & Kruuk, L.E.B. (2012) Genetic analysis of life-history constraint and evolution in a wild ungulate population. American Naturalist, 179, E97–E114.

[17] Morrissey, M.B. & Wilson, A.J. (2010) Pedantics: an R Package for pedigree-based genetic simulation and pedigree manipulation, characterization and viewing. Molecular Ecology Resources, 10, 711–719.

[18] Nichols, H.J., Cant, M.A., Hoffman, J.I. & Sanderson, J.L. (2014) Evidence for frequent incest in a cooperatively breeding mammal. Biology Letters, 10, 20140898.

[19] Nicolaus, M., Brommer, J.E., Ubels, R., Tinbergen, J.M. & Dingemanse, N.J. (2013) Exploring patterns of variation in clutch size-density reaction norms in a wild passerine bird. Journal of Evolutionary Biology, 26, 2031–2043.

[20] Saunders, S.P. & Cuthbert, F.J. (2014) Genetic and environmental influences on fitness-related traits in an endangered shorebird population. Biological Conservation, 177, 26–34.

[21] Tarka, M., Akesson, M., Hasselquist, D. & Hansson, B. (2014) Intralocus sexual conflict over wing length in a wild migratory bird. The American Naturalist, 183, 62–73.

[22] Tarka, M., Hansson, B. & Hasselquist, D. (2015) Selection and evolutionary potential of spring arrival phenology in males and females of a migratory songbird. Journal of Evolutionary Biology, 28,

1024–1038.

[23] Teplitsky, C., Mouawad, N.G., Balbontin, J., De Lope, F. & Møller, A.P. (2011) Quantitative genetics of migration syndromes: a study of two barn swallow populations. Journal of Evolutionary Biology, 24, 2025–2039.

[24] Tung, J., Barreiro, L.B., Burns, M.B., Grenier, J.-C., Lynch, J., Grieneisen, L.E., Altmann, J., Alberts, S.C., Blekhman, R. & Archie, E.A. (2015) Social networks predict gut microbiome composition in wild baboons. eLife, 4, e05224.

[25] Wilson, A.J., Morrissey, M.B., Adams, M.J., Walling, C.A., Guinness, F.E., Pemberton, J.M., Clutton-Brock, T.H. & Kruuk, L.E.B. (2011) Indirect genetics effects and evolutionary constraint: an analysis of social dominance in red deer, Cervus elaphus. Journal of Evolutionary Biology, 24, 772–783.

[26] Wolak, M.E. & Reid, J.M. Unpublished data.

References: Appendix S2

British Ornithologists' Union. (2013) The British List: A checklist of birds of Britain (8th edition). Ibis, 155, 635–676.

Morrissey, M.B. & Wilson, A.J. (2010) Pedantics: an R Package for pedigree-based genetic simulation and pedigree manipulation, characterization and viewing. Molecular Ecology Resources, 10, 711–719.

Appendix S3: Breeding value prediction

Breeding value reliability, or accuracy, will be affected by both missing phenotypic and pedigree information. Missing pedigree information also implies that the phenotypic data for an individual's relatives are also incomplete. In particular, predicted breeding values may be inaccurate when an individual with unknown parents also has an unknown phenotype itself. To illustrate the difference between true and predicted breeding values in such a situation, table S3.1 portrays a simple pedigree and three scenarios in which different forms of missing information might affect breeding value predictions.

Table S3.1. Breeding value prediction for phantom parents when (1) phenotypes are missing (NA) for all individuals (y_{obs1}), (2) only the phenotype for a focal individual (F1) was observed (y_{obs2}), or (3) the phenotype for focal individual (F1) is missing, but a phenotype was observed in F1's offspring F2 (y_{obs3}). Simulated breeding values (a_{true}) are listed for comparison to the predicted breeding values in scenarios (1) $\hat{a}_1$, (2) $\hat{a}_2$, or (3) $\hat{a}_3$. The table displays the bottom rows of a simulated data set and pedigree where PD and PS are phantom dam and sire and PM is the mate of F1 that was not observed, and F2 is the offspring of F1 and PM. The table shows all relatives of F1. The full phenotypic data set has a heritability of approximately $h^2 = 0.49$ and a mean trait value of approximately $\mu = 40.15$.

ID	Dam	Sire	y	a_{true}	y_{obs1}	$\hat{a}_1$	y_{obs2}	$\hat{a}_2$	y_{obs3}	$\hat{a}_3$
⋮	⋮	⋮	⋮	⋮	⋮	⋮	⋮	⋮	⋮	⋮
PD	NA	NA	41.05	3.12	NA	0.00	NA	-0.53	NA	-0.28
PS	NA	NA	42.79	-1.31	NA	0.00	NA	-0.53	NA	-0.28
F1	PD	PS	38.02	-2.87	NA	0.00	38.02	-1.05	NA	-0.57
PM	NA	NA	41.91	3.06	NA	0.00	NA	0.00	NA	-0.57
F2	F1	PM	37.86	-3.61	NA	0.00	NA	-0.53	37.86	-1.13

Scenario 1 in table S3.1 shows that when phenotypes are completely unavailable for an individual or any of its relatives, these individuals are assigned a predicted breeding value of zero ($\hat{a}_1$). When only a focal individual has an observed phenotype (F1 in scenario 2 and F2 in scenario 3), then this individual is assigned a predicted breeding value ($\hat{a}_2$) equal to the focal individuals phenotypic deviation from the population mean times the heritability (see eqn 2 in Postma 2006). The predicted breeding values for the focal individual's first order relatives are one-half (because relatedness=0.5, scenario 2 and 3) or for second order relatives one-quarter (because relatedness=0.25, scenario 3) of the focal individual's predicted breeding value. The differences between true (a_{true}) and predicted ($\hat{a}_1$, $\hat{a}_2$, $\hat{a}_3$) breeding values in Table S3.1 illustrate that, when phenotypic information is missing the predicted breeding values can be very inaccurate and more closely resemble phenotypic values. More formal evaluations of predicted breeding value accuracy are not pursued here since our aim is to highlight the simple point that missing phenotypic data from relatives (e.g., because

the relatives themselves are not connected to the focal individual through the pedigree) will increasingly cause predicted breeding values to be inaccurate.

References: Appendix S3

Postma, E. (2006) Implications of the difference between true and predicted breeding values for the study of natural selection and micro-evolution. Journal of Evolutionary Biology, 19, 309–320.

Appendix S4: Simulating data with genetic group effects

It is often useful to be able to simulate datasets on which animal models can be tested for bias, imprecision, or low power. The `rbv()` function in the `MCMCglmm` package can assign breeding values to a pedigree, given a specified variance-covariance matrix and genetic group means. However, `rbv` is limited to the supplied pedigree and cannot simulate trends over time in breeding values. We therefore provide the `R` function `simGG()`, included in the `nadiv` package ($\geq$v2.14.3), to simulate datasets with various combinations of population size, number of generations, number of "immigrants", differences among groups in breeding values, trends in breeding values, differences among groups in environmental deviations, or trends in environmental deviations. We used this function to simulate the data for the tutorials and presented in Figs 2 and 4 of the main text.

The basic code creates `K` individuals per generation (non-overlapping generations) for `g` generations, where `pairs` number of mating pairs are created by sampling with replacement from the adults of a given generation. Each pair contributes `noff` offspring to the next generation. During the simulation, `nimm` immigrants (individuals with unknown parent identities) are added to the population at generations specified by the sequence `nimmG`.

Individuals in the base population of the "focal" population are assigned total additive genetic values with a mean of `muf` and variance `VAf`. Offspring total additive genetic values `u` are the mean of their parents' `u` values plus a Mendelian sampling deviation drawn from a normal distribution with mean of 0 and variance equal to $0.5V_A(1 - f_{sd})$ where V_A is `VAf` and f_{sd} is the mean of the parents' coefficient of inbreeding f. Each "immigrant" (individual with unknown parents in generations >1) is assigned a total additive genetic effect that is drawn from a normal distribution with mean of `mui` and variance equal to `VAi`. Residual deviations are sampled for "focal" and "immigrant" populations separately, using normal distributions with means of `murf` and `muri`, respectively, and variances of `VRf` and `VRi`, respectively. Phenotypes are the sum of total additive genetic effects and residual deviations plus an overall mean `mup`.

Trends in total additive genetic effects and/or residual deviations can be specified for both the "focal" and "immigrant" populations. Trends in total additive genetic effects occurring in the "immigrants", in the residual deviations occurring in the "focal" population, and in the residual deviations occurring in the "immigrants" are all produced by altering the mean each generation for the separate distribution from which each of these effects are drawn. The change in mean over a generation is specified in units of standard deviations of the respective distributions (e.g., square roots of `VAi`, `VRf`, and `VRi`) and is set with `d_bvi`, `d_rf`, or `d_ri`, respectively. Trends in total additive genetic effects for the "focal" population are produced by

selecting individuals to be parents of the next generation according to their predicted total additive genetic effects. Individuals are assigned probabilities of being selected as a parent of the next generation depending on how closely a prediction of their total additive genetic effect matches an optimum value. The parameter `d_bvf` specifies how much the optimal total additive genetic effect changes per generation. `d_bvf` is multipled by the square root of `VAf` (i.e., the additive genetic standard deviation of the "focal" population) and the number of generations since selection began, and it is this quantity that is added to the base generation's mean total additive genetic effect (`muf`) to determine the optimal total additive genetic effect in a given generation. Individuals with predicted total additive genetic effects closest to this optimum have a higher probability of being randomly sampled to be parents of the next generation. This represents selection directly on predicted total additive genetic effects.

The function yields a `data.frame` with a row for every individual and columns: `id`, `dam`, and `sire` indicating individual identity, dam, and sire; `parAvgU` which is the individual's dam and sire average total additive genetic effect; `mendel` which is the Mendelian sampling deviate for an individual; `u` is the total additive genetic effect of each individual; `r` is the residual deviation; `p` is the individual's phenotype; `is` indicates `0` if the individual was born in the "focal" population or `1` if the individual is an "immigrant"; and `gen` specifies the generation in which each individual was born.

Details and examples of the function can be found in the help file associated with the `simGG()` function. These can be accessed by running the command `?simGG` in an `R` session.

4.1 Simulating a fixed difference between groups

The data in Figs 2 and 4 of the main text or the object `ggTutorial` of these tutorials, was simulated so that all additive genetic and residual variances are the same for both the founder population and immigrant populations (i.e., `VAf`=`VAi`=`VRf`=`VRi`=1), and there is no simulated temporal trend in either breeding values or residual deviations. Thus, we used the function with the following values:

```
set.seed(102)          #<-- value used to simulate 'ggTutorial' of these tutorials
ggTutorial <- simGG(K = 400, pairs = 200, noff = 4, g = 15,
    nimm = 40,
    muf = 0, mui = 3)
```

More information on the `ggTutorial` object can be found in the help file for this dataset, which can be accessed by running the command `?ggTutorial` in an `R` session.

4.2 Simulating a temporal trend in breeding values

Here is an example of simulating data where a positive trend in breeding values occurs in the immigrant population (e.g., response to directional natural selection in the immigrant population).

```
sim2df <- simGG(K = 400, pairs = 200, noff = 4, g = 15,
    nimm = 40,
    muf = 0, mui = 0,
    d_bvf = 0, d_bvi = 1)
```

```
# Now we can plot the phenotypic and genetic data
## Note the immigrants total additive genetic value increases each generation
par(mfrow = c(1, 2), cex = 0.7, cex.lab = 1, cex.axis = 0.7)
gens <- c(which(diff(sim2df$gen) == 1), nrow(sim2df))

plot(sim2df$p, axes = FALSE,
    main = "Phenotypic", xlab = "Generation", ylab = "Phenotype")
  axis(1, c(0, gens), labels = FALSE, tcl = 0.5)
  axis(1, gens-(0.5*gens[1]), labels = unique(sim2df$gen), tcl = 0)
  axis(2)

plot(sim2df$u, axes = FALSE,
    ylim = c(-5, 15),
    main = "Genetic", xlab = "Generation", ylab = "Total add. genetic effects")
  axis(1, c(0, gens), labels = FALSE, tcl = 0.5)
  axis(1, gens-(0.5*gens[1]), labels = unique(sim2df$gen), tcl = 0)
  axis(2, seq(-5, 15, 5))
```

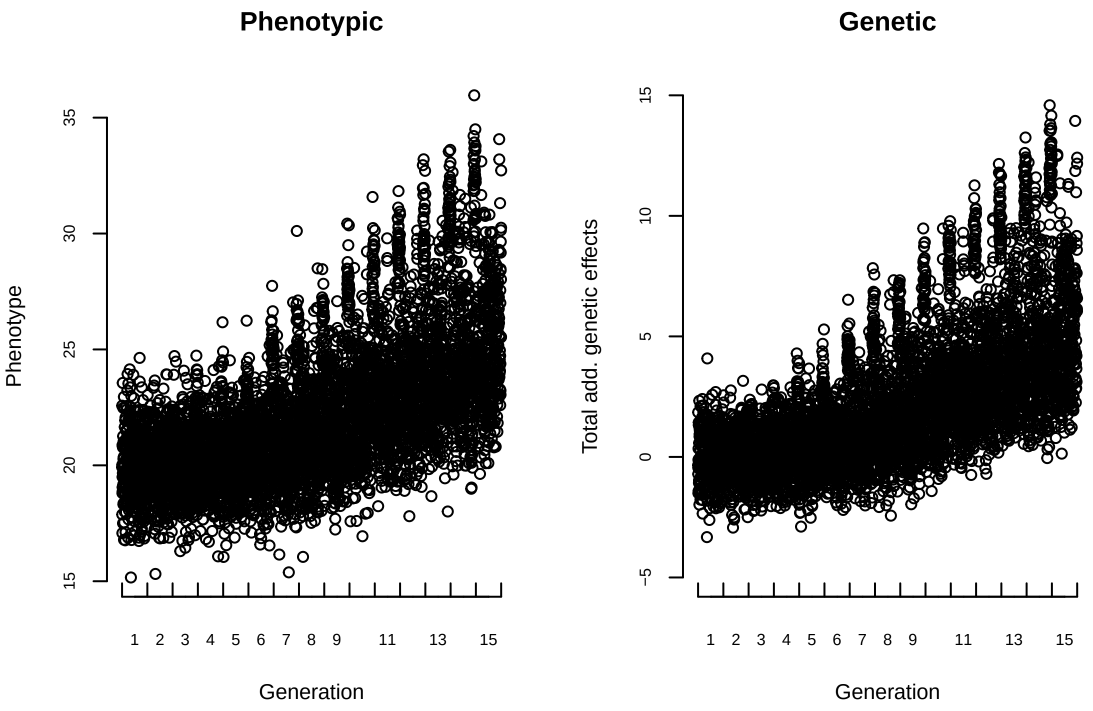

Analysing the above dataset with 6 genetic groups (divide generations in which immigrants occur into 6 groups) provides unbiased estimates of the simulated genetic group effects and additive genetic variance.

Appendix S5: Development of genetic groups in agricultural science

Genetic group methods have a long history in agricultural science and were first developed to control for selection in sire models (e.g., Henderson 1973). In a sire model, only the sire breeding values are modelled (Lynch & Walsh 1998, p.758; Mrode 2005, p.52). Genetic group effects are modelled in a sire model by nesting the random effects for each sire within fixed effects of sire herd and/or year (i.e., the genetic group), because sires may have different histories of artificial selection in their ancestry or belong to different herds. However, these models assume that all sires are unrelated and the genetic group effect applies to only the sire. Methodological advancements made it possible to first incorporate relatedness among sires and subsequently to incorporate the relatedness among all individuals through $\mathbf{A}$ (i.e., $\mathbf{A^{-1}}$ in an animal model). $\mathbf{A}$ accounts for selection if the model includes records for all individuals on which selection decisions are based and individuals in the base population have the same expected breeding value (Pollak and Quaas 1983). However, it is not always possible to obtain records for selection decisions when parents originate from different populations, often with different expected mean breeding values (Famula, Pollak & Van Vleck 1983). Including a model factor that reflects differences in breeding values due to selection, however, accommodates both of these issues. Thus, group effects were re-defined for use in animal models (Pollak & Quaas 1983; Famula, Pollak & Van Vleck 1983).

Robinson (1986) re-defined group effects in the context of relatedness among all individuals ($\mathbf{A}$) to reflect differences in breeding values among unknown parents. This allowed the animal model to further account for any selection that created differences in breeding values among unknown parents (Westell, Quaas & Van Vleck 1988). Under this model, each individual's group effect is the mean of its parents' group effects which ensures that similarities among individuals in group effects are consistent with relatedness. The contributions of genetic group effects to any one individual are proportional to the contribution of that genetic group to that individual's genome based on ancestry. Each genetic group's fractional contribution to each individual's genome can be calculated recursively from a pedigree and yields the matrix $\mathbf{Q}$. Estimating genetic group effects in a mixed model proceeds by fitting fixed covariate regressions of the fractional contributions from each group to every individual's genome (i.e., regressions on the columns of $\mathbf{Q}$) along with estimating every individual's breeding value (i.e., fitting a random additive genetic effect of every individual with $\mathbf{A^{-1}}$).

Fitting a fixed regression on columns of $\mathbf{Q}$, however, requires a post-analysis calculation to obtain the predicted total additive genetic effects of each individual. It is the total additive genetic effect that is of practical use in a selective breeding context. Therefore, Quaas & Pollak (1981) developed a method to modify the mixed model equations to directly obtain the total additive genetic effects of individuals as

the solutions to the model equations in a sire model. Westell, Quaas & Van Vleck (1988) showed how a similar transformation to that suggested by Quaas & Pollak (1981) could be used to transform the mixed model equations of an animal model. Such transformation leads to the absorption of genetic group effects into the vector of breeding values which produces predictions of total additive genetic effects directly from solutions to the model equations. The model also provides estimates of the genetic group effects themselves. The particular structure of these transformed equations, however, provides an additional benefit in setting up the model equations. The section of the transformed equations that deal with genetic group effects and total additive genetic effects are simple functions of $\mathbf{Q}$ and $\mathbf{A^{-1}}$. Since both $\mathbf{Q}$ and $\mathbf{A^{-1}}$ trace the flow of genes through a pedigree, this section of the transformed equations is a simple augmented inverse relatedness matrix ($\mathbf{A}^*$). The transformed equations are more efficiently set up by directly constructing and providing the augmented inverse relatedness matrix ($\mathbf{A}^*$) as opposed to constructing $\mathbf{Q}$ and $\mathbf{A^{-1}}$ first and then mathematically deriving the products of their different combinations that make up the transformed mixed model equations (Westell, Quaas & Van Vleck 1988; Quaas 1988). Constructing $\mathbf{A}^*$ follows the same basic rules as constructing $\mathbf{A^{-1}}$ directly from the pedigree. In practice, the average additive genetic differences among groups (i.e., the genetic group effects) are then estimated by providing $\mathbf{A}^*$ in the random effect syntax of animal model software. The solutions to the genetic group effects are still conceptually fixed effects, though they are returned by most animal model software programs in the vector of values associated with the random effect of individual total additive genetic effects (*Appendix S6*). Quaas (1988) formally demonstrated the equivalence between models that included genetic group effects by implementing either the fixed covariate regression or random effects specification of $\mathbf{A}^*$.

These basic genetic group methods have been extended to accommodate more complexities. First, genetic groups have been defined for maternal genetic effects (Van Vleck 1990). It is thus possible to allow the direct and maternal additive genetic effects to have different genetic groups (and hence different mean genetic effects) to reflect selection acting differently on these two components (Cantet et al. 1992). Secondly, Schaeffer (1991, 1994) has expanded the framework to include genetic group effects, conceptually, as random effects. As random effects, genetic groups are assumed to have a normal distribution with expectation of zero and a covariance model. Typically, random genetic group effects are modelled with a covariance structure of $\mathbf{I}\sigma^2_{\mathbf{g}}$, implying zero covariances among groups. More practically, the work by Schaeffer demonstrates how to remove potential dependencies among the group effects by simply making them random effects. This can be achieved by adding 1 to the diagonal values in the group coefficients partition of $\mathbf{A}^*$ (see further discussion in *Appendix S6.3.2*). More recently, grouping methods have progressed such that individuals with missing parents do not have to have their phantom parents assigned to a single group. In fuzzy classification of

genetic groups (Fikse 2009; *Appendix S7*), phantom parents are assigned to genetic groups with a probability of membership in each group. These classification methods are a simple extension to the methods for setting up $\mathbf{Q}$ and $\mathbf{A}^*$, but they have yet to be widely implemented. Finally, Misztal et al. (2013) have shown how to properly account for genetic group effects when fitting a combined genomic and pedigree derived relatedness matrix in the animal model.

References: Appendix S5

Cantet, R.J.C., Fernando, R.L., Gianola, D. & Misztal, I. (1992) Genetic grouping for direct and maternal effects with differential assignment of groups. Genetics, Selection, Evolution, 24, 211–223.

Famula, T.R., Pollak, E.J. & Van Vleck, L.D. (1983) Genetic groups in dairy sire evaluation under a selection model. Journal of Dairy Science, 66, 927–934.

Fikse, F. (2009) Fuzzy classification of phantom parent groups in an animal model. Genetics, Selection, Evolution, 41, 42–49.

Henderson, C.R. (1973) Sire evaluation and genetic trends. Journal of Animal Science, 1973, 10–41.

Lynch, M. & Walsh, B. (1998) Genetics and Analysis of Quantitative Traits. Sinauer Associates, Incorporated, Sunderland, MA.

Misztal, I., Vitezica, Z.G., Legarra, A., Aguilar, I. & Swan, A.A. (2013) Unknown-parent groups in single-step genomic evaluation. Journal of Animal Breeding and Genetics, 130, 252–258.

Mrode, R.A. (2005) Linear Models for the Prediction of Animal Breeding Values, 2nd ed. CABI Publishing, Cambridge, MA.

Pollak, E.J. & Quaas, R.L. (1983) Definition of group effects in sire evaluation models. Journal of Dairy Science, 66, 1503–1509.

Quaas, R.L. (1988) Additive genetic model with groups and relationships. Journal of Dairy Science, 71, 1338–1345.

Quaas, R.L. & Pollak, E.J. (1981) Modified equations for sire models with groups. Journal of Dairy Science, 64, 1868–1872.

Robinson, G.K. (1986) Group effects and computing strategies for models for estimating breeding values. Journal of Dairy Science, 69, 3106–3111.

Schaeffer, L.R. (1991) C. R. Henderson: Contributions to predicting genetic merit. Journal of Dairy Science, 74, 4052–4066.

Schaeffer, L.R. (1994) Multiple-country comparison of dairy sires. Journal of Dairy Science, 77, 2671–2678.

Van Vleck, L.D. (1990) Breeding value prediction with maternal genetic groups. Journal of Animal Science, 68, 3998–4013.

Westell, R.A., Quaas, R.L. & Van Vleck, L.D. (1988) Genetic groups in an animal model. Journal of Dairy Science, 71, 1310–1318.

Appendix S6: Construction and implementation of Q and A*

Fitting animal models with genetic groups first necessitates formulating the appropriate $\mathbf{Q}$ and/or $\mathbf{A}^*$ matrices. First, we generally describe how $\mathbf{Q}$ and $\mathbf{A}^*$ are formed, with an emphasis on the code used to do this in the `R` package `nadiv` (>v2.14.0; Wolak 2012). This is followed by explanations of minor changes that can be made to $\mathbf{A}^*$ to improve animal model convergence and how to deal with warnings from software regarding singularities and non-positive definite matrices. Next are sections with code that illustrate fitting genetic group animal models in the `MCMCglmm` and `asreml` packages in `R`, the **ASReml** standalone program, and finally the **WOMBAT** standalone program. In each of these four sections, code and examples are provided to fit genetic groups using the `nadiv` package in `R` to create the $\mathbf{Q}$ and $\mathbf{A}^*$ matrices that can then be supplied to these animal model fitting programs (in some cases outside of the `R` environment), followed by examples using existing functionality within each software program (see Table S6.1 for a summary of these programs and their capabilities).

The tutorials for these software programs all use the simulated dataset (`ggTutorial` available in `nadiv` or to download in **WOMBAT** and **ASReml** standalone formats) depicted in Figs 2 and 4 of the main text and described below. In *Appendix S4*, the `R` code in the `nadiv` function `simGG()` that is used to simulate the `ggTutorial` dataset is explained. Details of package and software program versions are available at the end of the supporting information.

Table S6.1. Overview of animal model software highlighted in the tutorials. The genetic group capabilities row contains a description of the functionality that is available solely within each software program. The **Form Q** and **Form A*** rows indicate if each program can create the **Q** or **A*** matrices, respectively, from a supplied pedigree. We indicate whether fitting genetic groups explicitly (as fixed effects) or implicitly within the random effects structure (as either fixed or random effects) is recommended (+) or not (-) when using each software program.

	`MCMCglmm`	`ASReml` in `R`	**ASReml**	**WOMBAT**
**Genetic group capabilities**	None in `MCMCglmm`, but can use **A*** or **Q** created with `nadiv`	The `asreml.Ainverse()` function creates **A***	The `!GROUPS` qualifier to the pedigree creates **A*** and see the `!LAST` and `!GOFFSET` qualifiers (details below)	Random genetic group effects (with separate variance component from the additive genetic variance) by including `GENGROUPS` in a `SPECIAL` block with a supplied **Q** (created with `nadiv`)
**Form Q**	No	No	No	No
**Form A***	No	Yes	Yes	No
**Explicit approach**	+	+	+	+
**Implicit approach**				
Fixed	-	+	+	-
Random	+	+	+	+
**Free to use**	Yes	No	No	Yes
**Reference**	Hadfield 2010	Butler et al. 2009	Gilmour et al. 2014	Meyer 2007

6.1 A small example pedigree

Below we demonstrate how the matrices **Q** and $\mathbf{A}^*$ are constructed using an example pedigree from Quaas (1988). A detailed version of the Quaas pedigree is available in the `nadiv` package in `R` as the `Q1988` dataset:

id	dam	sire	damGG	sireGG	phantomDam	phantomSire	group
g1	NA	NA	NA	NA	NA	NA	NA
g2	NA	NA	NA	NA	NA	NA	NA
a	NA	NA	g1	g1	NA	NA	g1
b	NA	NA	g2	g2	NA	NA	g2
c	NA	NA	g2	g2	NA	NA	g2
d	NA	NA	g1	g1	NA	NA	g1
e	NA	NA	g2	g2	NA	NA	g2
A	NA	NA	g1	g2	a	b	NA
B	A	NA	A	g2	A	c	NA
C	NA	NA	g1	g2	d	e	NA
D	B	C	B	C	B	C	NA

The table above is essentially just a pedigree (first three columns indicate an individual's unique identifying code, dam, and sire). However, two rows have been added at the top of the pedigree for the unique genetic groups and extra columns are included for ease of using all possible pedigree formats accepted by the `nadiv` functions that implement genetic group methods.

In the dataset, "g1" and "g2" are two genetic groups, lower case letters are phantom parent identities, and upper case letters are observed individual identities. The genetic groups and phantom parents are assigned as in Quaas (1988). Additional details regarding the `Q1988` dataset in `nadiv` are in the `R` help file that can be viewed by running the command `?Q1988` in an `R` session.

Below, we will only deal with observed individuals in this dataset, such that the subset of `Q1988` we will use is:

```
Q1988sub <- Q1988[-c(3:7), c("id", "damGG", "sireGG")]
```

id	damGG	sireGG
g1	NA	NA
g2	NA	NA
A	g1	g2
B	A	g2
C	g1	g2
D	B	C

Note, this is not the only format for including genetic group information in functions of the `nadiv` package. To see a full description of alternative formats see the function help files, particularly for the `ggcontrib` (to make **Q**) and `makeAinv` (to make $\mathbf{A}^*$) functions.

6.2 Constructing Q

As described in the main text, the matrix **Q** contains the fractional contributions from each of r genetic groups to each individual's genome, calcluated from the pedigree (see also Robinson 1986; Mrode 2005, ch. 2). Therefore, if there are n individuals in the pedigree (e.g., n=4 in `Q1988sub`), **Q** will be an n row by r column matrix. In Henderson's (1976) decomposition of the numerator relationship matrix (**A=TDT'**), the lower triangle matrix **T** reflects the contribution of each individual in the pedigree to every other individual. In other words, **T** traces the flow of genes from one generation to another. Therefore, if we add the r genetic groups to the top of the pedigree (originally of length n) as identities with unknown dam and sire (NA) and fill in genetic group labels as dam and sire identities of every individual with a missing parent (such as how `Q1988sub` is arranged) we can obtain the **T** matrix for a pedigree of length n+r. The first r columns of the **T** matrix contain the fractional contributions from each genetic group. Therefore, **Q** for the n individuals in a pedigree is the r+1 to r+n rows and the first r columns of **T**.

The `nadiv` function `ggcontrib()` constructs **Q** for a given pedigree with r potential genetic group contributions to each of the n individuals in the pedigree. For example, **Q** for `Q1988sub` is obtained:

```
(Q <- ggcontrib(Q1988sub))
```

```
##      g1    g2
## A 0.500 0.500
```

```
## B 0.250 0.750
## C 0.500 0.500
## D 0.375 0.625
```

Each row in **Q** (each entry is a proportional contribution) sums to one and offspring inherit half of each genetic group contribution from each of their parents. Individuals `A` and `C` in `Q1988sub` each have a phantom dam from genetic group "g1" and phantom sire from genetic group "g2". These individuals correspond to the first and third rows of **Q** above. As expected, the two genetic groups (each column in **Q**) contribute equally to the genomes of `A` and `C`.

Individual `B` has observed dam `A` and a phantom sire from genetic group "g2". Row two of **Q** above is a sum of each parents' row (genetic group contributions) divided by two: dam `A [0.500 0.500]/2` plus phantom sire "g2" `[0.000 1.000]/2`. In other words, individual `B` gets half of dam `A`'s "g1" group contribution (`0.500/2`) and its phantom sire from "g2" does not contribute to the proportion of `B`'s genome derived from "g1". Similarly, individual `B` gets half of dam `A`'s "g2" group contribution (`0.500/2`) plus half of the phantom sire "g2" contribution (`1.000/2`).

Finally, individual `D` has observed dam `B` and observed sire `C`. Therefore, row four of **Q** above is the sum of each parents' row (genetic group contributions) divided by two: dam `B [0.250 0.750]/2` plus sire `C` `[0.500 0.500]/2`.

Implementing an animal model with genetic group effects fitted explicitly as separate fixed regressions requires the **Q** matrix. Because every row of **Q** sums to one, it is not possible to solve an animal model to obtain estimates for every genetic group effect and an overall model intercept. Thus, genetic group effects are not estimable themselves, but can only be estimated when expressed as deviations (or functions) from other group effects (Lynch & Walsh 1998, p.839-841). Therefore, a column/genetic group is chosen as the "reference group" and is assigned a genetic group effect of 0 (see practical implementation of this below). All other group effects will represent deviations from this reference group. For example, we might choose genetic group "g1" in the `Q1988sub` pedigree as our reference group. The estimate of the fixed regression slope from the second column of **Q** equals the "g2" genetic group mean expressed as a deviation from the reference group ("g1") mean of zero. If we had used a pedigree with more than two genetic groups, still setting the first group as the reference, then the genetic group estimates for each group are all deviations from the "g1" reference group mean of zero.

Q is also needed to calculate breeding values (**a**) from an animal model that has fitted fixed genetic group effects implicitly within the random effects syntax of the model software. This is because such a model

will yield predictions of total additive genetic effects ($\mathbf{u}$). From equation (6) in the main text, an individual's breeding value (a_i) equals the total additive genetic effect (u_i) minus a weighted sum of genetic group effects contributing to that individual. The weighted sum of genetic group contributions for all individuals can be calculated as the matrix product $\mathbf{Qg}$, where $\mathbf{g}$ is a vector of all genetic group effects and $\mathbf{Q}$ is calculated above.

6.3 Constructing A*

A major advance leading to the widespread use of animal models comes from Henderson (1976) and subsequent researchers' (e.g., Quaas 1976, 1995; Meuwissen and Luo 1992) work to directly construct the matrix inverse of the additive genetic relatedness matrix ($\mathbf{A^{-1}}$). Direct methods for obtaining $\mathbf{A^{-1}}$ are necessary, because $\mathbf{A^{-1}}$ and not $\mathbf{A}$ is used when solving the equations in the animal model. $\mathbf{A^{-1}}$ has a unique structure that can be formed by tracing the flow of alleles from each individual to its offspring. Thus methods to directly construct $\mathbf{A^{-1}}$ rely on the basic premise that the flow of alleles through a pedigree can be traced because each individual inherits, on average, half of its alleles from each parent with some sampling variation that can be modeled based on the Mendelian sampling variance in the population.

To estimate genetic group effects by implicitly fitting the genetic groups within the random effect syntax of the model software, requires an inverse matrix to model the covariance among individual total additive genetic values and group effects based on alleles shared identical by descent. Conveniently, the matrix inverse accounting for individual-individual, group-group, group-individual, and individual-group sharing of alleles can be constructed directly from the pedigree in a very similar manner to $\mathbf{A^{-1}}$ (see also *Appendix S5*; Westell, Quaas & Van Vleck 1988; Quaas 1988). This matrix, $\mathbf{A}^*$ (following notation of Quaas 1988), is a compilation of four sub-matrices that are each a mathematical function of $\mathbf{Q}$, $\mathbf{A^{-1}}$, or both (Fig. 3e in main text).

The $\mathbf{A}^*$ for any pedigree with genetic groups can be obtained using the `makeAinv()` function in the `nadiv` package. For example, $\mathbf{A}^*$ for the `Q1988sub` pedigree is:

```
AstarOut <- makeAinv(Q1988sub, ggroups = 2)
(Astar <- AstarOut$Ainv)
```

```
## 6 x 6 sparse Matrix of class "dgCMatrix"
##
## A   1.3333333 -0.6666667  .     . -0.5 -0.1666667
## B  -0.6666667  1.8333333  0.5 -1  .   -0.6666667
```

```
## C   .           0.5000000  1.5 -1 -0.5 -0.5000000
## D   .          -1.0000000 -1.0  2  .    .
## g1 -0.5000000  .          -0.5  .  0.5  0.5000000
## g2 -0.1666667 -0.6666667 -0.5  .  0.5  0.8333333
```

There are several points to highlight regarding $\mathbf{A}^*$. First, this matrix can be split into four blocks: (1) the upper-left 4-by-4 (nxn) block (individual-individual covariance structure), (2) the lower-left 2-by-4 (rxn) block (group-individual covariance structure), (3) the upper-right 4-by-2 (nxr) block (individual-group covariance structure), and (4) the lower-right 2-by-2 (rxr) block (group-group covariance structure). Note, the upper-left block of $\mathbf{A}^*$ is the same as the $\mathbf{A^{-1}}$ for the `Q1988` pedigree without implicitly including genetic group effects (see also Fig. 3e in main text). For example, $\mathbf{A^{-1}}$ for the Q1988 pedigree without genetic groups can be obtained and compared to $\mathbf{A}^*$ above using the same `nadiv` function without invoking the genetic group algorithm [`makeAinv(..., ggroups = NULL, ...)`, the default value of the `ggroups` argument]:

```r
makeAinv(Q1988[-c(1:7), c("id", "dam", "sire")])$Ainv
```

```
## 4 x 4 sparse Matrix of class "dgCMatrix"
##
## A  1.3333333 -0.6666667  .    .
## B -0.6666667  1.8333333  0.5 -1
## C  .          0.5000000  1.5 -1
## D  .         -1.0000000 -1.0  2
```

$\mathbf{A}^*$ constructed in `nadiv` can be used in various animal model software programs (Table S6.1) to implicitly include genetic group effects within the random effects structure of the model. Although the details vary among software programs (see tutorials in the subsections to *Appendix S6.4* for detailed instructions in each of the highlighted software programs), the general approach is to associate the inverse covariance matrix $\mathbf{A}^*$ with the term in the model representing additive genetic effects; almost exactly the same way as the typical $\mathbf{A^{-1}}$ is associated with additive genetic effects in a basic animal model. However, some potential alterations to $\mathbf{A}^*$ should be considered before and during the model fitting process.

6.3.1 Genetic groups on top or bottom of A*?

The entries in $\mathbf{A}^*$ corresponding to genetic groups can be placed in the last r rows and columns (as above) or the first r rows and columns. When setting up $\mathbf{A}^*$, switching between these placements is trivial. The

consequences for using $\mathbf{A}^*$, however, can be rather large. Solving an animal model requires solving a large set of equations (the Mixed Model Equations or MME). Often, some of these equations may not be unique or in other words two equations may be exactly the same, very similar, or have a linear dependency (such that knowing the solution to one equation means the solution to the other equation is also known). This is a cause of singularities in the MME coefficient matrix of an animal model.

The MME are solved by either moving from the first set of equations to the last set ('top-down') or from the last set to the first set ('bottom-up'). In either case, if two equations are nearly the same then the model will obtain a solution for the first of the pair, but then encounter a problem with the second. Some software packages might automatically deal with this second, non-unique equation. For example, **ASReml** solves the equations from the bottom up and will delete the second equation (equation on top) to continue to solve the MME (Butler et al. 2009, pp. 134-135; Gilmour et al. 2014, pp. 106-107).

Unfortunately, the algorithms to solve the MME are not this simple in practice. Even though **ASReml** states which way the equations are solved, some options allow the program to re-order the equations to simplify solving the model. This re-ordering is based on the contents of each equation without respect to its categorization in the model. In other words, one genetic group equation might be re-ordered so that it is placed next to an equation for a year random effect level while another genetic group equation in the same model may be placed elsewhere with a random effect of individual additive genetic value. Although one cannot be completely certain where the genetic group equations will end up when the software program optimizes the order of equations to most efficiently solve the model, the ability to easily switch where in $\mathbf{A}^*$ the genetic groups are positioned has been a strategy that has sometimes worked well in our own personal experience. Switching the placement of the genetic groups from the top to the bottom (or vice versa) of $\mathbf{A}^*$ has sometimes solved convergence issues.

However, the standalone version of **ASReml** has the `!LAST` job qualifier (see Table S6.1 and the **ASReml** tutorial in *Appendix S6.4.4*) to consistently re-order the MME such that the genetic groups equations are solved last. This option assumes that the genetic groups are the first r rows and columns of $\mathbf{A}^*$, where r is the number of levels following the `!LAST` qualifier, and therefore 'on top'. We are not aware of a similar argument that can be implemented for a model using `asreml` in `R`.

WOMBAT, also, has several MME ordering strategies that can be implemented. However, these re-order the entire set of equations and the order of certain levels of random effects cannot be specified (Meyer 2007, section 5.3.1). `MCMCglmm` re-orders the MME according to algorithms associated with the underlying `CSparse` functions (Davis 2006; Hadfield 2010) and also cannot designate certain levels of random effects to a certain location in the re-ordered equations. Therefore, it is not clear how specifying genetic groups at the

top or bottom of $\mathbf{A}^*$ will affect the convergence of any particular model.

The nadiv function `makeAinv()`, enables the user to place genetic groups at the top or bottom of $\mathbf{A}^*$, in an attempt to solve genetic group equations first or last. Therefore, if any singularities in the MME coefficient matrix occur then the user can attempt to re-order the genetic group equations in the hope that this will lead to model convergence. The `gOnTop` argument to the `makeAinv()` function switches between placing the genetic groups on top or on bottom of $\mathbf{A}^*$, with the default to place genetic groups at the bottom of $\mathbf{A}^*$ [`makeAinv(..., gOnTop = FALSE, ...)`].

6.3.2 Removing singularities and other problems within A*

Fitting genetic group effects implicitly often causes singularities in the coefficient matrix of the MME, that can sometimes be overcome by slight changes to the strategy for grouping (Schaeffer 1991). Software programs may report this issue as a non-positive definite generalized inverse matrix (often abbreviated GIV, GIN, or ginverse), or alternatively stating that a GIV/GIN/ginverse is negative definite, non-positive semi-definite, or ill-conditioned. For example, **ASReml** often deals with these types of singularities according to its own strategies (Butler et al. 2009; Gilmour et al. 2014). If genetic groups are specified in an **ASReml** analysis and a singularity occurs the program will introduce a row to the matrix to overcome this (see *Lagrangian multipliers* in Gilmour et al. 2014, pp. 164-165). Regardless of which software program is used, however, adding values to some of the matrix elements may also remove singularities occurring within the MME in addition to the aforementioned strategy of ordering $\mathbf{A}^*$ with genetic groups either on top or at the bottom.

Adding a small number to some diagonals can remove singularities (Schaeffer 1994, 1999; Oikawa and Yasuda 2009; Gilmour 2010). Schaeffer (1994, 1999) advocates adding an identity matrix to the rxr group-group block of $\mathbf{A}^*$ such that all of the diagonals (d_{ii}) become $1 + d_{ii}$. This addition to $\mathbf{A}^*$ means that the genetic group effects are no longer, conceptually speaking, fixed effects but are random effects with expectations of zero and a variance describing their distribution (see *Appendices S1 & S5*). Further, predicted genetic group effects will be biased toward the expected value of zero when genetic groups are random effects, just like any other random effects, with the amount of bias depending on how much information the data provide to predict genetic group effects (Hadfield et al. 2010). This bias will extend to any functions involving the predicted genetic group effects, specifically the predicted total additive genetic effects ($\mathbf{u}$). Although, in many cases the mixed model equations and subsequent variance component estimates change only slightly, thus basic interpretations of results will remain the same (but see cautionary note below; further discussion in Schaeffer 1994). The strategy of adding an identity matrix is desirable if there are many individuals assigned to each group, because it does not re-rank the predicted breeding values and can improve convergence

(Schaeffer 1994). The main change to the model when the leading diagonal elements of the group-group portion of $\mathbf{A}^*$ are altered is a (co)variance matrix for traits within groups is added to the model equations dealing with the genetic group effects. In the simple case of a single trait, this variance is assumed to be the same across group effects with no covariances between groups (i.e., groups effects are normally distributed with a variance of $\mathbf{I}\sigma^2_{\mathbf{g}}$). In practice this variance is often assumed to equal the additive genetic variance for the individual total additive genetic effects (Sullivan 1999, see also **WOMBAT**'s genetic groups in *Appendix S6.4.5.4*).

A further interpretation of this assumption means that a model's estimate of additive genetic variance will also include the magnitudes of the genetic group effects. This may cause problems if the variance in the genetic group effects themselves is much greater than the true additive genetic variance. In such a case, adding a smaller value than 1 to the diagonals (d_{ii}) of $\mathbf{A}^*$ structures the model to reflect this difference. Using values other than 1 for the addition to the diagonal elements effectively means that the ratio of the variance among genetic group effects to the additive genetic variance is no longer constrained to be 1:1. Thus, when modelling genetic groups as random effects we strongly caution users to carefully consider the sensitivity of the additive genetic variance estimate to the choice of value added to the group-group diagonal elements of $\mathbf{A}^*$. Multiple models fitted with different values added to the diagonal elements are recommended to ensure an unbiased estimate of the additive genetic variance. Schaeffer (1994) provides a detailed discussion and interpretation of different strategies for altering $\mathbf{A}^*$.

Note that **WOMBAT** fits a separate variance component to the genetic group effects (see *Appendix S6.4.5.4*), thus offering a direct way to check that the variance in genetic group effects can be assumed to be the same as the additive genetic variance. **ASReml** provides the `!GOFFSET` pedigree file qualifier to add a value to the group-group diagonal elements when using **ASReml**'s `!GROUPS` pedigree qualifier (see *Appendix S6.4.4.4*). Otherwise, this alteration of $\mathbf{A}^*$ is easily accomplished manually after creating $\mathbf{A}^*$ with the `makeAinv()` function in the `nadiv` package. Altering the $\mathbf{A}^*$ for the `Q1988sub` pedigree above (with genetic groups on the bottom) would proceed as follows and result in the matrix displayed below:

```
n <- 4
r <- 2
(ggIdentity <- bdiag(Diagonal(n = n, x = 0), Diagonal(n = r, x = 1)))
```

```
## 6 x 6 sparse Matrix of class "dtCMatrix"
##
## [1,] 0 . . . . .
```

```
## [2,] . 0 . . . .
## [3,] . . 0 . . .
## [4,] . . . 0 . .
## [5,] . . . . 1 .
## [6,] . . . . . 1
```

```
(AstarI <- Astar + ggIdentity)
```

```
## 6 x 6 sparse Matrix of class "dgCMatrix"
##
## A   1.3333333 -0.6666667  .    . -0.5 -0.1666667
## B  -0.6666667  1.8333333  0.5 -1  .   -0.6666667
## C   .          0.5000000  1.5 -1 -0.5 -0.5000000
## D   .         -1.0000000 -1.0  2  .    .
## g1 -0.5000000  .         -0.5  .  1.5  0.5000000
## g2 -0.1666667 -0.6666667 -0.5  .  0.5  1.8333333
```

Caution is necessary, however, when dealing with singularities in an animal model. Specifically, model comparison may not be possible if singularities occur. **ASReml** alters the order of equations to remove singularities. Consequently, different terms can cause singularities between runs of the model, where the model may have been slightly modified for the purposes of testing the significance of variance components or computing profile likelihoods of variance components. Singularities caused by different terms will affect the log-likelihood of the model and therefore procedures using likelihood ratio test statistics may no longer be valid (Gilmour 2005; Butler et al. 2009; Gilmour et al. 2014)

6.4 How to fit genetic groups in MCMCglmm, ASReml-R, ASReml-standalone, & WOMBAT

6.4.1 Tutorial dataset `ggTutorial`

We provide a step-by-step demonstration of how to fit genetic groups in animal models, by modelling genetic group effects either explicitly as separate fixed regressions or implicitly within the random effects, using the different animal model software programs. All demonstrations use the dataset `ggTutorial`, which is available in the `nadiv` package for working in `R`, as supplementary file `ggTutorial.dat` for working in the

standalone **ASReml** program, and as supplementary file `ggTutorial.d` for working in the **WOMBAT** program. This dataset was simulated using the `nadiv` package (details in *Appendix S4*) and are the same data as those depicted in Figs 2 and 4 from the main text. The dataset contains 6000 individuals across 15 generations. The simulation specifies a carrying capacity of 400 individuals per generation, 200 mating pairs per generation, and 40 immigrants per generation. The expected difference between founder and immigrant mean breeding values equals `3` and both the environmental and additive genetic variances for both the founder and immigrant groups equal `1`. The data are loaded into `R` by loading the `nadiv` package.

```
library(nadiv)
```

The first three columns of `ggTutorial` contain the complete pedigree and all 6000 individuals have a phenotypic record in `p`.

```
head(ggTutorial)
```

```
##   id dam sire parAvgU mendel          u           r        p is gen
## 1  1  NA   NA      NA     NA -0.3928101 -0.08355464 19.52364  0   1
## 2  2  NA   NA      NA     NA -0.6750007  0.14201698 19.46702  0   1
## 3  3  NA   NA      NA     NA -0.4416978  1.89195823 21.45026  0   1
## 4  4  NA   NA      NA     NA -1.0299707 -1.29820650 17.67182  0   1
## 5  5  NA   NA      NA     NA -1.9126222  0.46681834 18.55420  0   1
## 6  6  NA   NA      NA     NA -0.9089290 -0.82577884 18.26529  0   1
```

```
str(ggTutorial)
```

```
## 'data.frame':    6000 obs. of  10 variables:
##  $ id     : int  1 2 3 4 5 6 7 8 9 10 ...
##  $ dam    : int  NA NA NA NA NA NA NA NA NA NA ...
##  $ sire   : int  NA NA NA NA NA NA NA NA NA NA ...
##  $ parAvgU: num  NA NA NA NA NA NA NA NA NA NA ...
##  $ mendel : num  NA NA NA NA NA NA NA NA NA NA ...
##  $ u      : num  -0.393 -0.675 -0.442 -1.03 -1.913 ...
##  $ r      : num  -0.0836 0.142 1.892 -1.2982 0.4668 ...
##  $ p      : num  19.5 19.5 21.5 17.7 18.6 ...
```

```
##  $ is     : int  0 0 0 0 0 0 0 0 0 0 ...
##  $ gen    : int  1 1 1 1 1 1 1 1 1 1 ...
```

The rest of the columns contain:

- `parAvgU` is the average u (total additive genetic effect) of each individual's parents
- `mendel` is the Mendelian sampling deviation from the mid-parent value that is unique to each individual
- `u` is the total additive genetic effect of each individual's genotype
- `r` is the residual deviation of each individual
- `p` is the phenotype for each individual (population expected mean is 20)
- `is` indicates the immigration status, where focal population residents have `is == 0` and immigrants have `is == 1`
- `gen` is the generation in which each individual was born

The supplementary files for use in **ASReml** and **WOMBAT** contain a subset of these variables plus three derived variables. The **ASReml** and **WOMBAT** data files contain continuous variables for the coefficient of inbreeding (f) and two genetic group genomic contributions (from $\mathbf{Q}$). For demonstration purposes, these are added to the basic `ggTutorial` below within each section. Note the **WOMBAT** pedigree and data files have had 10000 added to the individual identity codes to comply with **WOMBAT** data structure requirements.

It is necessary to fit a fixed regression on the coefficient of inbreeding (f) to account for inbreeding depression and minimize bias in estimates of quantitative genetic parameters (Kennedy, Schaeffer & Sorensen 1988; Reid & Keller 2010; Wolak & Keller 2014). This is not directly part of the process to fit genetic groups in the animal model, but it is considered necessary when fitting animal models to population data in which inbreeding occurs.

6.4.2 `MCMCglmm`

Modelling genetic groups either explicitly as separate fixed regressions or implicitly within the random effects syntax of `MCMCglmm` requires either $\mathbf{Q}$ or $\mathbf{A}^*$ to be created using the `nadiv` package. Below we demonstrate how to create these matrices and fit each in a `MCMCglmm` model.

```r
library(MCMCglmm)
library(nadiv)
```

To complete the basic preparations of the data for an animal model, all that is necessary is to calculate the coefficients of inbreeding (f; to minimize bias from inbreeding depression, see the last paragraph of *Appendix 6.4.1* above). This calculation can be done using the `inverseA()` function of `MCMCglmm`. Consequently, the $\mathbf{A^{-1}}$ matrix is also created, which we will assign as its own object in `R`, because it will be needed in the model fitting genetic group effects explicitly as fixed covariate regressions with $\mathbf{Q}$ (see *Appendix 6.4.2.2*).

```r
ainvOut <- inverseA(ggTutorial[, 1:3])
Ainv <- ainvOut$Ainv
ggTutorial$f <- ainvOut$inbreeding
```

`MCMCglmm` facilitates modelling of non-Gaussian response variables, which has greatly extended the application of animal models in evolutionary ecology to a range of non-Gaussian phenotypes. Fitting genetic groups is no different when modelling non-Gaussian response variables. In such models, the genetic group effects (fitted either explicitly as separate fixed regressions or implicitly within the random effects) are estimated on the underlying latent scale.

6.4.2.1 Preparing a pedigree with genetic groups Here, we provide general instructions to prepare pedigrees. Calculating the matrix of genetic group contributions ($\mathbf{Q}$) and the augmented inverse relatedness matrix ($\mathbf{A}^*$) requires a pedigree that indicates groups instead of missing values for parents. In the `ggTutorial` data, we will assign all individuals with unknown parents in the first generation phantom parents from one genetic group (`foc0`). All individuals in subsequent generations that have unknown parents will be assigned phantom parents from a second genetic group (`g1`). Alternatively, this second group could be further divided into genetic groups based on generation. However, the data were simulated such that every immigrant has the same expected mean breeding value, regardless of the generation in which the immigrant was born. Therefore, we will stick to a total of two genetic groups (but, feel free to model more groups!).

First, create an object that will be the pedigree with genetic groups (`ggPed`).

```r
ggPed <- ggTutorial[, c("id", "dam", "sire", "is", "gen")]
naPar <- which(is.na(ggPed[, 2]))
ggPed$GG <- rep("NA", nrow(ggPed))
```

```r
  # 'focal' genetic group = "foc0" and 'immigrant' = "g1"
  # obtained by pasting "foc" & "g" with immigrant status "0" or "1", respectively
  ggPed$GG[naPar] <- as.character(ggPed$is[naPar])
  ggPed$GG[ggPed$GG == "0"] <- paste0("foc", ggPed$GG[ggPed$GG == "0"])
  ggPed$GG[ggPed$GG == "1"] <- paste0("g", ggPed$GG[ggPed$GG == "1"])
ggPed[naPar, 2:3] <- ggPed[naPar, "GG"]
```

Note, the approach and format of the pedigree above is different from the `Q1988sub` pedigree earlier - mostly because the format here makes it easier to specify genetic groups in a pedigree for this particular case, but partly to illustrate the flexibility of `nadiv` functions. To be more specific, the `Q1988sub` pedigree contained two extra rows for the two genetic groups. The `ggroups` argument to `makeAinv()` supplied an integer indicating how many rows at the begining of the pedigree contained genetic groups and not individuals. However, in the `ggPed` above no extra rows are added to the pedigree, but in the next few sections the character vector given to the `ggroups` argument in both `ggcontrib()` and `makeAinv()` specifies the name of the unique genetic groups that have been filled in for individuals instead of missing dam and sire identities.

6.4.2.2 Fixed explicit genetic group effects with Q (from `nadiv`) Fitting genetic group effects explicitly requires the columns of **Q** to be included as separate fixed covariate regressions. The code below creates **Q** for the `ggPed` pedigree and adds the columns (`foc0` and `g1`) as variables in `ggTutorial` so that they can be included in a model.

```r
Q <- ggcontrib(ggPed[, 1:3], ggroups = c("foc0", "g1"))
ggTutorial <- cbind(ggTutorial, Q)
str(ggTutorial)
```

```
## 'data.frame':    6000 obs. of  13 variables:
##  $ id     : int  1 2 3 4 5 6 7 8 9 10 ...
##  $ dam    : int  NA NA NA NA NA NA NA NA NA NA ...
##  $ sire   : int  NA NA NA NA NA NA NA NA NA NA ...
##  $ parAvgU: num  NA NA NA NA NA NA NA NA NA NA ...
##  $ mendel : num  NA NA NA NA NA NA NA NA NA NA ...
##  $ u      : num  -0.393 -0.675 -0.442 -1.03 -1.913 ...
##  $ r      : num  -0.0836 0.142 1.892 -1.2982 0.4668 ...
```

```
## $ p     : num  19.5 19.5 21.5 17.7 18.6 ...
## $ is    : int  0 0 0 0 0 0 0 0 0 0 ...
## $ gen   : int  1 1 1 1 1 1 1 1 1 1 ...
## $ f     : num  0 0 0 0 0 0 0 0 0 0 ...
## $ foc0  : num  1 1 1 1 1 1 1 1 1 1 ...
## $ g1    : num  0 0 0 0 0 0 0 0 0 0 ...
```

An important point to make is that, because the pedigree and data for `ggTutorial` are in the same dataframe, the genetic group coefficients from $\mathbf{Q}$ can be added directly to the data with `ggTutorial <- cbind(ggTutorial, Q)` above. However, whenever a pedigree contains more individuals than the data (or in a different order), an intermediate step is required. Specifically, since the $\mathbf{Q}$ matrix contains a row for every individual in the pedigree, only the subset of rows in $\mathbf{Q}$ that correspond to phenotyped individuals in a dataset should be extracted and ordered according to the identities in the data.

The fixed regressions on `foc0` and `g1` can be combined with the standard $\mathbf{A^{-1}}$ to specify an animal model in `MCMCglmm`. Below, we write out the specification of the `MCMCglmm` default priors for the fixed effects in the model. We recommend a normal prior distribution for each genetic group fixed regression coefficient with a mean of 0 and a very large variance. Our fixed effect prior specification below has an element in `mu` and `V` of `prPE$B` to correspond with the model's: intercept, regression on the coefficient of inbreeding (f), and regressions on the genetic group contributions (`foc0` and `g1`). Because unique solutions to the model do not exist (see discussion of estimability in *Appendix S6.2*) for the intercept, coefficient of inbreeding (f), and genetic group effects (`foc0` and `g1`), we fit the `foc0` genetic group as the last fixed effect. This ensures that the model sets `foc0` as a reference group and estimates the `g0` genetic group effect as a deviation from the mean breeding value in the `foc0` group. The model is written out below, and a saved version is available on Dryad (Wolak & Reid 2016) as supporting information file "./MCMCglmm/ggRegMC.RData" so that it is not necessary to re-run this model to examine the results.

```
# Use diffuse normal priors (MCMCglmm defaults) for all fixed effects
# Parameter expanded prior on additive genetic variance
## Scaled F-dsitribution with numerator and denominator df=1
prPEexp <- list(B = list(mu = rep(0, 3), V = diag(3)*1e10),
         R = list(V = 1, nu = 0.002),
         G = list(G1 = list(V = 1, nu = 1, alpha.mu = 0, alpha.V = 1000)))
```

```r
# NOTE: the saved model could be loaded instead of running it below
## load(file = "./MCMCglmm/ggRegMC.RData")
ggRegMC <- MCMCglmm(fixed = p ~ f + g1 + foc0,
    random = ~ id,
    ginverse = list(id = Ainv),
    data = ggTutorial,
    prior = prPEexp,
    singular.ok = FALSE,
    pr = TRUE,
    nitt = 28000, burn = 3000, thin = 25)
```

The posterior distributions of genetic group effects are included in the `Sol` object along with the posterior distributions of the other fixed effects. Note the software has dropped the `foc0` group estimate in this model, implying this is the reference group and the `g1` genetic group effects is the estimated deviation from the reference. To report the posterior modes and 95% limits of the highest posterior density:

```r
with(ggRegMC, cbind(postMode = posterior.mode(Sol[, 1:3]),
    HPDinterval(Sol[, 1:3])))
```

```
##               postMode     lower     upper
## (Intercept) 19.7952878 19.664293 19.931933
## f            0.3756279 -2.652013  4.529125
## g1           3.1375194  2.941707  3.285130
```

The above posterior mode estimate of 3.138 agrees with the expected difference of `3` between the mean breeding values of the immigrant group (`3`) and the founder population (`0`), specified in the simulation.

The posterior distribution of the breeding values (**a**) can be accessed from `ggRegMC$Sol[, -c(1:3)]`. Because the order of rows in **Q** matches the order of individual identities in `ggTutorial`, the posterior distribution for the total additive genetic effects (**u**) can be calculated as (eqn 5 in main text):

```r
# Add 0 genetic group effect for the reference group 'foc0' to the 'g1' samples
regPost_gHat <- rbind(0, ggRegMC$Sol[, "g1"])
# For the ith mcmc iteration that was saved, calculate 'u'
```

```r
regPost_u <- as.mcmc(t(sapply(seq(nrow(ggRegMC$Sol)),
    FUN = function(i){Q %*% regPost_gHat[, i] + ggRegMC$Sol[i, -c(1:3)]})))
```

6.4.2.3 Random implicit genetic group effects with A* (from `nadiv`) Fitting genetic group effects implicitly within the random effects portion of the `MCMCglmm` model specification requires the augmented inverse **A** matrix ($\mathbf{A}^*$) to be supplied as a sparse matrix in the `ginverse` argument. The code below creates $\mathbf{A}^*$ for the `ggPed` pedigree using the `makeAinv` function in the `nadiv` package.

```r
Astar <- makeAinv(ggPed[, 1:3], ggroups = c("foc0", "g1"), gOnTop = TRUE)$Ainv
```

In the above code, we directed `makeAinv()` to include the genetic groups on top (top-left block) of $\mathbf{A}^*$ using the `gOnTop = TRUE` argument. At this point we could try fitting the model with this version of $\mathbf{A}^*$ (i.e., `Astar`). However, the model returns an error that `G-structure 1 is ill-conditioned`. In other words, $\mathbf{A}^*$ has at least one eigenvalue that is either negative or zero. Re-defining the groupings can eliminate such a singularity. However, removing the singularity in the matrix while maintaining the current groupings is accomplished by adding a small value to the diagonal elements of the group-group portion of the matrix. Note that such an addition changes the interpretation of the effects in the model, as discussed above (*Appendix 6.3.2* as well as *Appendix S5*), now implying that the genetic group effects are random effects in the model. Below, we add 0.1 to the diagonal element of the $\mathbf{A}^*$ which will constrain the variance in genetic group effects to be one tenth the additive genetic variance estimated by the model. A value of 0.1 may bias estimates of additive genetic variance in models of other datasets. Therefore, we advise testing values other than 0.1 (e.g., 1 or 10) and comparing estimates from these alternative models. Note that a value of 1 constrains the variance in genetic group effects to be equal to the additive genetic variance estimated by the model.

```r
AstarAdd <- Astar
(ggrows <- match(c("foc0", "g1"), dimnames(AstarAdd)[[1]]))
```

```
## [1] 1 2
```

```r
diag(AstarAdd)[ggrows] <- diag(AstarAdd)[ggrows] + 0.1
Astar[1:3, 1:3]
```

```
## 3 x 3 sparse Matrix of class "dgCMatrix"
##
```

```
## foc0 400   . -1
## g1      . 520  .
## 1     -1    .  1
```

```
AstarAdd[1:3, 1:3]
```

```
## 3 x 3 sparse Matrix of class "dgCMatrix"
##
## foc0 400.1   .    -1
## g1      .    520.1  .
## 1     -1.0   .     1
```

The `AstarAdd` sparse inverse matrix can now be associated with the individual identities (`id`) by supplying it as an argument to `ginverse` in the `MCMCglmm` model. We specify the same default prior distribution for the fixed effects below (i.e., the same ones as we wrote out in *Appendix 6.4.2.2*). The prior distribution for the `id` term in the model is the prior used for the additive genetic variance and, in this case, also the same prior for the variance of the genetic group effects. It is unclear how the prior specified for the additive genetic variance will affect the estimate of the genetic group effects or even what prior is to be used in this context. As usual with Bayesian analyses, we recommend priors that are framed within the context of the data at hand, the model being fitted, and any *a priori* belief in the model parameters. Further we suggest testing the sensitivity of any particular prior used for a given model and especially when genetic group effects are treated as random effects by the model.

The model is written out below, but a saved version is available on Dryad (Wolak & Reid 2016) as supporting information file "./MCMCglmm/ggAstarRandMC.RData" so that it is not necessary to re-run this model to examine the results.

```
# Parameter expanded prior on additive genetic variance
prPEimp <- list(B = list(mu = rep(0, 2), V = diag(2)*1e10),
         R = list(V = 1, nu = 0.002),
         G = list(G1 = list(V = 1, nu = 1, alpha.mu = 0, alpha.V = 1000)))


# NOTE: the saved model could be loaded instead of running it below
## load(file = "./MCMCglmm/ggAstarRandMC.RData")
ggAstarRandMC <- MCMCglmm(fixed = p ~ f,
```

```r
    random = ~ id,
    ginverse = list(id = AstarAdd),
    data = ggTutorial,
    prior = prPEimp,
    pr = TRUE,
    nitt = 53000, burn = 3000, thin = 50)
```

Alternatively, the non-altered matrix `Astar` could be supplied to the `ginverse` argument of `MCMCglmm` to see the types of warnings that led us to fit the model with the matrix `AstarAdd`.

The posterior distributions of genetic group effects are included in the `Sol` object (as long as `pr = TRUE` in the model statement) along with the posterior distributions of the fixed effects. To report the posterior modes and 95% limits of the highest posterior density:

```r
with(ggAstarRandMC, cbind(postMode = posterior.mode(Sol[, 1:4]),
    HPDinterval(Sol[, 1:4])))
```

```
##              postMode     lower     upper
## (Intercept) 22.023119 16.604035 25.597429
## f            1.348870 -2.842843  4.164732
## id.foc0     -2.164536 -5.801390  3.099577
## id.g1        0.786011 -2.742551  6.267101
```

Note that all fixed effects in this model are estimable and the model returns these estimates along with predicting both of the genetic group effects. The genetic group predictions, themselves, are random effects and thus are deviations from their expected value of 0. The posterior mode of differences between genetic group predictions 3.107 (95% HPD interval:2.945, 3.277) agrees with the expected difference between the mean breeding values of the immigrant group (`3`) and the founder population (`0`) that was specified in the simulation.

The posterior distribution of the total additive genetic effects ($\mathbf{u}$) can be accessed from `ggAstarRandMC$Sol[, -c(1:4)]`. Because the order of rows in $\mathbf{Q}$ matches the order of individual identities in `ggTutorial`, the posterior distribution for the breeding values ($\mathbf{a}$) can be calculated as (eqn 6 in main text):

```r
AstarPost_gHat <- ggAstarRandMC$Sol[, 3:4]
# For the ith mcmc iteration that was saved, calculate 'a'
# Assuming Q has already been calculated as above
AstarPost_a <- as.mcmc(t(sapply(seq(nrow(ggAstarRandMC$Sol)),
    FUN = function(i){ggAstarRandMC$Sol[i, -c(1:4)] -
            Q %*% matrix(AstarPost_gHat[i, ], ncol = 1)})))
```

6.4.3 `ASReml` in `R`

To model genetic group effects explicitly as separate fixed regressions in an animal model using the `R` package `asreml` requires $\mathbf{Q}$ to be created using the `nadiv` package and then included in the `asreml()` model function. Including genetic group effects implicitly within the random effects of an animal model in `asreml()` can be done either using $\mathbf{A}^*$ constructed with `nadiv` or with `asreml::asreml.Ainverse()`. All three of these approaches will be demonstrated below using the simulated dataset `ggTutorial`:

```r
library(asreml)
library(nadiv)
```

To complete the basic preparations of the data for an animal model, all that is necessary is to calculate the coefficients of inbreeding (f; to minimize bias from inbreeding depression, see the last paragraph of *Appendix 6.4.1*). This calculation can be done using the `asreml.Ainverse()` function of `asreml`. Consequently, the $\mathbf{A}^{-1}$ matrix is also created, which we will assign as its own `R` object, because it will be needed in the model fitting genetic group effects explicitly as separate fixed covariate regressions with $\mathbf{Q}$ below (*Appendix 6.4.3.2*).

```r
ainvOut <- asreml.Ainverse(ggTutorial[, 1:3])
listAinv <- ainvOut$ginv
ggTutorial$f <- ainvOut$inbreeding
## Have to also convert the 'id' variable to a factor for models below
ggTutorial$id <- as.factor(ggTutorial$id)
```

6.4.3.1 Preparing a pedigree with genetic groups Calculating the matrix of genetic group contributions ($\mathbf{Q}$) and the augmented inverse relatedness matrix ($\mathbf{A}^*$) requires a pedigree that has genetic groups

indicated instead of missing values for parents. In the `ggTutorial` data, we will assign all individuals with unknown parents in the first generation phantom parents from one genetic group (`foc0`). All individuals in subsequent generations that have unknown parents will be assigned phantom parents from a second genetic group (`g1`). Alternatively, this second group could be further divided into genetic groups based on generation. However, the data were simulated such that every immigrant has the same expected mean breeding value, regardless of the generation in which it was born. Therefore, we will stick to a total of two genetic groups (but, feel free to model more groups!).

First, create an object that will be the pedigree with genetic groups (`ggPed`).

```
ggPed <- ggTutorial[, c("id", "dam", "sire", "is", "gen")]
naPar <- which(is.na(ggPed[, 2]))
ggPed$GG <- rep("NA", nrow(ggPed))
  # 'focal' genetic group = "foc0" and 'immigrant' = "g1"
  # obtained by pasting "foc" & "g" with immigrant status "0" or "1", respectively
  ggPed$GG[naPar] <- as.character(ggPed$is[naPar])
  ggPed$GG[ggPed$GG == "0"] <- paste0("foc", ggPed$GG[ggPed$GG == "0"])
  ggPed$GG[ggPed$GG == "1"] <- paste0("g", ggPed$GG[ggPed$GG == "1"])
ggPed[naPar, 2:3] <- ggPed[naPar, "GG"]
# Two rows need to be added for the genetic groups
## Genetic groups will be given the missing value NA instead of parent identities
ggPed <- data.frame(id = c("foc0", "g1", as.character(ggPed$id)),
        dam = c(NA, NA, as.character(ggPed$dam)),
        sire = c(NA, NA, as.character(ggPed$sire)))
head(ggPed)
```

```
##     id  dam sire
## 1 foc0 <NA> <NA>
## 2   g1 <NA> <NA>
## 3    1 foc0 foc0
## 4    2 foc0 foc0
## 5    3 foc0 foc0
## 6    4 foc0 foc0
```

Note that the `nadiv` function `makeAinv()` used to construct $\mathbf{A}^*$ is more flexible than `asreml`'s

asreml.Ainverse() in the way pedigrees containing genetic groups can be formatted (for more detail on makeAinv() formats, see the help file by running the command ?makeAinv in an R session - particularly the examples at the end of the file - or the genetic group pedigree used above in the MCMCglmm tutorial). To be more specific, the format required by asreml.Ainverse() is similar to the Q1988sub pedigree used earlier in the tutorial (*Appendix 6.1*) which contains two extra rows for the two genetic groups.

6.4.3.2 Fixed explicit genetic group effects with Q (from nadiv) Fitting genetic group effects explicitly in asreml requires the columns of **Q** to be included as separate fixed covariate regressions. The code below creates **Q** for the ggPed pedigree and adds the columns (foc0 and g1) as variables in ggTutorial so that they can be included in a model.

```
Q <- ggcontrib(ggPed)
ggTutorial <- cbind(ggTutorial, Q)
```

```
str(ggTutorial)
```

```
## 'data.frame':    6000 obs. of  13 variables:
##  $ id     : Factor w/ 6000 levels "1","2","3","4",..: 1 2 3 4 5 6 7 8 9 10 ...
##  $ dam    : int  NA NA NA NA NA NA NA NA NA NA ...
##  $ sire   : int  NA NA NA NA NA NA NA NA NA NA ...
##  $ parAvgU: num  NA NA NA NA NA NA NA NA NA NA ...
##  $ mendel : num  NA NA NA NA NA NA NA NA NA NA ...
##  $ u      : num  -0.393 -0.675 -0.442 -1.03 -1.913 ...
##  $ r      : num  -0.0836 0.142 1.892 -1.2982 0.4668 ...
##  $ p      : num  19.5 19.5 21.5 17.7 18.6 ...
##  $ is     : int  0 0 0 0 0 0 0 0 0 0 ...
##  $ gen    : int  1 1 1 1 1 1 1 1 1 1 ...
##  $ f      : num  0 0 0 0 0 0 0 0 0 0 ...
##  $ foc0   : num  1 1 1 1 1 1 1 1 1 1 ...
##  $ g1     : num  0 0 0 0 0 0 0 0 0 0 ...
```

An important point to make is that, because the pedigree and data for ggTutorial have essentially the same sizes and structures (only ggPed now has two extra rows for the genetic groups - but these will be dropped automatically from **Q** by ggcontrib()), the genetic group coefficients from **Q** can be added directly

to the data with `ggTutorial <- cbind(ggTutorial, Q)` above. However, whenever a pedigree contains more individuals than the data (or in a different order), an intermediate step is required. Specifically, since the **Q** matrix contains a row for every individual in the pedigree, only the subset of rows in **Q** that correspond to phenotyped individuals in a dataset should be extracted and ordered according to the identities in the data.

The fixed regressions on `foc0` and `g1` can be combined with the standard $\mathbf{A}^{-1}$ to specify an animal model in `asreml()`. Because unique solutions to the model do not exist (see discussion of estimability in *Appendix S6.2*) for the intercept, coefficient of inbreeding (f), and genetic group effects (`foc0` and `g1`), we fit the `foc0` genetic group as the last fixed effect. This ensures that the model sets `foc0` as a reference group and estimates the `g0` genetic group effect as a deviation from the mean breeding value in the `foc0` group.

```
ggRegASR <- asreml(fixed = p ~ f + g1 + foc0,
    random = ~ ped(id),
    ginverse = list(id = listAinv),
    data = ggTutorial)
```

The estimates of the genetic group effects are included with the fixed effect estimates. Note the software has set the `foc0` group estimate to zero in this model, implying this is the reference group and the `g1` genetic group effect is the estimated deviation. The variance of the estimated genetic group effect (`g1`) is included with the variances of the fixed effect estimates, which are reported on `asreml`'s underlying transformed scale. To re-scale the variances of the fixed effects back to the phenotypic scale and report them as standard errors:

```
with(ggRegASR, cbind(Effect = coefficients$fixed,
    seEffect = sqrt(vcoeff$fixed * sigma2)))
```

```
##                 Effect   seEffect
## foc0         0.0000000 0.00000000
## g1           3.1167796 0.08929429
## f            0.6660162 1.83248941
## (Intercept) 19.7976160 0.06579030
```

The above estimate of 3.117 agrees with the expected difference between mean breeding value in the immigrant group (`3`) and the founder population (`0`) that was specified in the simulation.

The predicted breeding values ($\mathbf{a}$) can be accessed from `ggRegASR$coefficients$random`. Because the order of rows in $\mathbf{Q}$ matches the order of individual identities in `ggTutorial`, the total additive genetic effects ($\mathbf{u}$) can be calculated without any further manipulation as (eqn 5 in main text):

```
reg_gHat <- matrix(ggRegASR$coefficients$fixed[1:2], ncol = 1)
reg_u <- Q %*% reg_gHat + ggRegASR$coefficients$random
```

Note, if more individuals are in the pedigree than the dataset and/or the identities in the pedigree and data are ordered differently, more coding is necessary to ensure that the predicted genetic effect for a particular individual is added to the correct weighted genetic group contribution. Additionally, if more random effects are included in the model, care has to be taken in order to ensure the correct random effect predictions are extracted for the breeding values ($\mathbf{a}$). The R function `match()` is particularly helpful in these cases.

6.4.3.3 Fixed implicit genetic group effects with A* (from `nadiv` or `asreml`) Fitting fixed effects of genetic groups implicity within the random effects of an animal model requires the augmented inverse relatendess matrix ($\mathbf{A}^*$) to be supplied as a list in the `ginverse` argument. First, we create the sparse matrix of $\mathbf{A}^*$ for the `ggPed` pedigree using the `makeAinv` function in the `nadiv` package and then demonstrate how to obtain this same matrix with the `asreml` package.

```
listAstar <- makeAinv(ggPed, ggroups = 2, gOnTop = FALSE)$listAinv
```

In the above code, we directed `makeAinv()` to include the genetic groups on bottom (bottom-right block) of $\mathbf{A}^*$ using `gOnTop = FALSE`. This was done to improve model convergence (see *Appendix 6.3* and its subsections). In this case, the `asreml` model converges. In other pedigrees, models, and/or genetic group classifications, if singularities occur then sometimes this can be overcome by adding one (or a small value) to the diagonal elements of the group-group portion of the matrix (although see discussion of this in *Appendix S5* and cautions/considerations in *Appendix 6.3.2*). The alteration is demonstrated below along with cautions and interpretations (*Appendix 6.4.3.4*). Note, the easiest approach is to adjust the genetic group diagonals of $\mathbf{A}^*$ using the matrix returned by `makeAinv()` (i.e., object `Ainv` in the list), then convert the matrix to the list format required by `asreml`.

Alternatively, we can use `asreml`'s function `asreml.Ainverse()` to obtain a list format of $\mathbf{A}^*$.

```r
listAstar <- asreml.Ainverse(ggPed, groups = 2)$ginv
```

CAUTION: under the current and previous versions of `asreml` (current version at the end of Supporting Information), quite often `asreml.Ainverse()` simply does not work. It will return an object with a `ginv` entry that is a `data.frame`, but with zero rows. Sometimes, re-running the above command will work - otherwise use `nadiv`'s `makeAinv()` (*Appendix 6.4.3*)! Note that in the above code, `asreml.Ainverse()` includes the genetic groups on top (top-left block) of $\mathbf{A}^*$. Often models fitting this structure of $\mathbf{A}^*$ have trouble converging due to this matrix not being positive-definite (see *Appendix 6.3* and its subsections). One strategy to remedy this is to place the genetic groups on the bottom of $\mathbf{A}^*$. Since there is no easy option to specify this in `asreml.Ainverse()` we recommend using `nadiv`'s function `makeAinv()` as demonstrated above.

Regardless of which package was used to create `listAstar`, this sparse inverse matrix in list format has two extra properties ('attributes') that are critical for being able to use it in an `asreml` model. First (and not specific to just genetic group models), all `asreml` ginverse objects must have the "rowNames" attribute to map row/column numbers back to the factor levels in the model variable. Second, specific to models fitting fixed genetic groups implicitly within the random effects the `listAstar` object must have the "geneticGroups" attribute where the first number indicates the number of unique genetic groups. This is essential for `asreml` to correctly calculate the residual degrees of freedom as part of the residual variance and log-likelihood calculations. These attributes are the last two rows of the internal object structure:

```r
str(listAstar)
```

```
## 'data.frame':    19642 obs. of  3 variables:
##  $ row   : int  1 2 3 4 5 6 7 8 9 10 ...
##  $ column: num  1 2 3 4 5 6 7 8 9 10 ...
##  $ Ainv  : num  1 1.5 1 1 1.5 1 1 1 1 2 ...
##  - attr(*, "rowNames")= chr  "1" "2" "3" "4" ...
##  - attr(*, "geneticGroups")= num  2 0
```

Both of these attributes are discussed in the help file to `makeAinv()` and can be accessed by typing `?makeAinv` in `R`. Now the object `listAstar` can be associated with the individual identities (`id`) in the model by supplying the list to the `ginverse` argument in the `asreml()` function.

```r
ggAstarASR <- asreml(fixed = p ~ f,
    random = ~ ped(id),
    ginverse = list(id = listAstar),
    data = ggTutorial)
```

The estimate of the genetic group effects are included with the random effect predictions. Since we directed `makeAinv()` to position the genetic groups on the bottom of `Astar`, the genetic group effects are the last two predicted values. The variance of the estimated genetic group effects are included with the variances of the random effect predictions, which are reported on `asreml`'s underlying transformed scale. To re-scale the variances of the fixed effects and genetic group effects back to the phenotypic scale and report these as standard errors along with the estimates themselves:

```r
# find the location of the genetic groups
(ggrows <- match(c("foc0", "g1"), attr(listAstar, "rowNames")))
```

```
## [1] 6001 6002
```

```r
# genetic group and fixed effects (with re-scaled standard errors)
with(ggAstarASR, cbind(Effect = c(coefficients$fixed, coefficients$random[ggrows]),
    seEffect = sqrt(c(vcoeff$fixed, vcoeff$random[ggrows]) * sigma2)))
```

```
##                   Effect   seEffect
## f              0.6660162 1.83248941
## (Intercept)    0.0000000 0.00000000
## ped(id)_foc0  19.7976160 0.06579030
## ped(id)_g1    22.9143956 0.06008392
```

Note the software has set the intercept estimate to zero in this model (see discussion of estimability in *Appendix S6.2*), implying this is the reference to which the `foc0` and `g1` genetic group effects are the estimated deviations. Therefore, the predicted total additive genetic effects ($\mathbf{u}$) include the overall phenotypic mean. However, the difference between the genetic group effects 3.117 agrees with the expected difference between the mean breeding values of the immigrant group (`3`) and the founder population (`0`) that was specified in the simulation.

The predicted total additive genetic effects ($\mathbf{u}$) can be accessed from `ggAstarASR$coefficients$random[-ggrows]`. Because the order of rows in $\mathbf{Q}$ matches the order of individual identities in `ggTutorial`, the breeding values ($\mathbf{a}$) can be calculated without any further manipulation as (eqn 6 in main text):

```
Astar_gHat <- matrix(ggAstarASR$coefficients$random[ggrows], ncol = 1)
Astar_a <- ggAstarASR$coefficients$random[-ggrows] - Q %*% Astar_gHat
```

Note, if more individuals are in the pedigree than the dataset and/or the identities in the pedigree and data are ordered differently, more coding is necessary to ensure that the predicted genetic effect for a particular individual is added to the correct weighted genetic group contribution. Additionally, if more random effects are included in the model, care has to be taken in order to ensure the correct random effect predictions are extracted for the total additive genetic effects ($\mathbf{u}$) and used to calculate breeding values ($\mathbf{a}$). The `R` function `match()` is particularly helpful in these cases.

6.4.3.4 Random implicit genetic group effects with A* (from `nadiv`) Fitting random effects of genetic groups implicity within the random effects of an animal model requires the augmented inverse relatendess matrix ($\mathbf{A}^*$) to be supplied as a list in the `ginverse` argument. First, we create the sparse matrix of $\mathbf{A}^*$ for the `ggPed` pedigree using the `makeAinv` function in the `nadiv` package, then make the necessary alterations (see discussion of this in *Appendix S5* and cautions/considerations in *Appendix 6.3.2*) so the model interprets the genetic groups as random effects, convert the sparse matrix to a list, and fit the model.

```
Astar <- makeAinv(ggPed, ggroups = 2, gOnTop = FALSE)$Ainv
```

In the above code, we directed `makeAinv()` to include the genetic groups on bottom (bottom-right block) of $\mathbf{A}^*$ using `gOnTop = FALSE`. This was done to improve model convergence (see *Appendix 6.3* and its subsections).

Next we add a small value to the diagonal elements of the group-group portion of the matrix taking the same approach as in the `MCMCglmm` tutorial above (*Appendix 6.4.2.3*). As a result of this alteration, the genetic group effects are conceptually random effects in the model. Below, we add 0.1 to the diagonal element of the $\mathbf{A}^*$ which will constrain the variance in genetic group effects to be one tenth the additive genetic variance estimated by the model. A value of 0.1 may bias estimates of additive genetic variance in models of other datasets. Therefore, we advise testing values other than 0.1 (e.g., 1 or 10) and comparing estimates from these alternative models. Note that a value of 1 constrains the variance in genetic group effects to be equal to the additive genetic variance estimated by the model.

The easiest approach is to adjust the genetic group diagonals of $\mathbf{A}^*$, and this is why we stored the matrix returned by `makeAinv()` above (i.e., object `Ainv` in the list). Once the additions are made, we can then convert the matrix to the list format required by `asreml`.

```
AstarAdd <- Astar
(ggrows <- match(c("foc0", "g1"), dimnames(AstarAdd)[[1]]))
```

```
## [1] 6001 6002
```

```
diag(AstarAdd)[ggrows] <- diag(AstarAdd)[ggrows] + 0.1
Astar[ggrows, ggrows]
```

```
## 2 x 2 sparse Matrix of class "dgCMatrix"
##
## foc0 400   .
## g1     . 520
```

```
AstarAdd[ggrows, ggrows]
```

```
## 2 x 2 sparse Matrix of class "dgCMatrix"
##
## foc0 400.1   .
## g1     .   520.1
```

```
# convert to the list format using `nadiv::sm2list()`
listAstarAdd <- sm2list(AstarAdd, rownames = rownames(AstarAdd))
# Should be NO "geneticGroups" attribute (or element 1 of this attribute must==0)
str(listAstarAdd)
```

```
## 'data.frame':    19642 obs. of  3 variables:
##  $ row   : int  1 2 3 4 5 6 7 8 9 10 ...
##  $ column: num  1 2 3 4 5 6 7 8 9 10 ...
##  $ A     : num  1 1.5 1 1 1.5 1 1 1 1 2 ...
##  - attr(*, "rowNames")= chr  "1" "2" "3" "4" ...
```

The object `listAstarAdd` can be associated with the individual identities (`id`) in the model by supplying the list to the `ginverse` argument in the `asreml()` function.

```
ggAstarRanASR <- asreml(fixed = p ~ f,
    random = ~ ped(id),
    ginverse = list(id = listAstarAdd),
    data = ggTutorial)
```

The predictions of the genetic group effects are included with the random effect predictions. Since we directed `makeAinv()` to position the genetic groups on the bottom of `Astar`, the genetic group effects are the last two predicted values. The variance of the random effect predictions are reported on `asreml`'s underlying transformed scale. To re-scale the variances of the genetic group predictions back to the phenotypic scale and report these as standard errors along with the predictions themselves and the fixed effect estimates (for comparison to other models):

```
# find the location of the genetic groups
(ggrows <- match(c("foc0", "g1"), attr(listAstarAdd, "rowNames")))
```

```
## [1] 6001 6002
```

```
# genetic group and fixed effects (with re-scaled standard errors)
with(ggAstarRanASR, cbind(Effect = c(coefficients$fixed, coefficients$random[ggrows]),
    seEffect = sqrt(c(vcoeff$fixed, vcoeff$random[ggrows]) * sigma2)))
```

```
##                    Effect seEffect
## f               0.6656207 1.832498
## (Intercept)    21.3560577 2.320737
## ped(id)_foc0   -1.5578210 2.320741
## ped(id)_g1      1.5578209 2.320741
```

Note the intercept in this model is estimable and so the model returns this estimate along with predicting both of the genetic group effects. The genetic group predictions, themselves, are random effects and thus are deviations from their expected value of 0. The difference between the genetic groups 3.116 agrees with the expected difference between the mean breeding values of the immigrant group (`3`) and the founder population (`0`) that was specified in the simulation.

The predicted total additive genetic effects ($\mathbf{u}$) can be accessed from `ggAstarRanASR$coefficients$random[-ggrows]`. Here, the prediced total additive genetic effects include the genetic group effects expressed as deviations from 0. Because the order of rows in $\mathbf{Q}$ matches the order of individual identities in `ggTutorial`, the breeding values ($\mathbf{a}$) can be calculated without any further manipulation as (eqn 6 in main text):

```
AstarRan_gHat <- matrix(ggAstarRanASR$coefficients$random[ggrows], ncol = 1)
AstarRan_a <- ggAstarRanASR$coefficients$random[-ggrows] - Q %*% AstarRan_gHat
```

Note, if more individuals are in the pedigree than the dataset and/or the identities in the pedigree and data are ordered differently, more coding is necessary to ensure that the predicted genetic effect for a particular individual is added to the correct weighted genetic group contribution. Additionally, if more random effects are included in the model, care has to be taken in order to ensure the correct random effect predictions are extracted for the total additive genetic effects ($\mathbf{u}$) and used to calculate breeding values ($\mathbf{a}$). The `R` function `match()` is particularly helpful in these cases.

6.4.4 ASReml Standalone

Here we cover four approaches to fit genetic groups in an **ASReml** analysis. The first two use `nadiv` functions in `R` to create $\mathbf{Q}$ and $\mathbf{A}^*$, which are each separately included in **ASReml** models. The third approach uses **ASReml** arguments and qualifiers to rely entirely upon the **ASReml** capabilities for fitting genetic groups. Whereas the first three approaches are used to fit genetic groups as fixed effects, the final approach demonstrates how to fit random effects of genetic groups.

The preparation of the `ggTutorial` data and requisite outputs from `nadiv` in `R` will first be demonstrated before detailing the code used in the **ASReml** model fitting. However, the basic files necessary to fit the **ASReml** models without any preparation in `R` are available in the accompanying supporting files on Dryad (Wolak & Reid 2016). The content of these files is summarized as:

- `ggTutorial.dat` contains the `ggTutorial` data. This file contains space separated columns from a subset of columns in the original `data.frame` in the following order: `id`, `p`, `is`, `gen`, `f`, `foc0`, and `g1`. This is needed for all models fitted.
- `reg.ped` contains the pedigree and is analogous to the first three columns of `ggTutorial`. This is needed to fit a model with genetic group effects explicitly as separate fixed regressions in the model.

- `nadivAstar.giv` and `nadivAstar.txt` are needed to fit genetic group effects implicitly within the random effects of the model using `nadiv`'s `makeAinv()` in `R` to create $\mathbf{A}^*$ (contained in `nadivAstar.giv`). `nadivAstar.txt` provides the row names for `nadivAstar.giv` and is needed to associate levels in `nadivAstar.giv` to identities in the data `ggTutorial.dat`. Further explanation is provided below where these files are created.

- `asremlGG.ped` contains the genetic group pedigree. It is the same as `reg.ped` except two rows are added for each of the two genetic groups and individuals do not have missing values, but genetic groups, specified for missing dams and sires. This is needed to fit genetic group effects implicitly within the random effects using **ASReml**'s own capabilities.

- `ggReg.as`, `ggNadivAstarFxd.as`, `ggAsremlAstarFxd.as`, and `ggAsremlRan.as` are included within the folders with similar names. Each specifies a different model to run.

To prepare the data for an animal model, all that is necessary is to calculate the coefficients of inbreeding (f; to minimize bias from inbreeding depression, see the last paragraph of *Appendix 6.4.1*). This calculation can be done using the `makeAinv()` function in the `nadiv` package.

```
library(nadiv)
```

```
ggTutorial$f <- makeAinv(ggTutorial[, 1:3])$f
```

6.4.4.1 Preparing a pedigree with genetic groups Calculating the matrix of genetic group contributions ($\mathbf{Q}$) and the augmented inverse relatedness matrix ($\mathbf{A}^*$) requires a pedigree that has genetic groups indicated instead of missing values for parents. In the `ggTutorial` data, we will assign all individuals with unknown parents in the first generation phantom parents from one genetic group (`foc0`). All individuals in subsequent generations that have unknown parents will be assigned phantom parents from a second genetic group (`g1`). Alternatively, this second group could be further divided into genetic groups based on generation. However, the data were simulated such that every immigrant has the same expected mean breeding value, regardless of the generation in which it was born. Therefore, we will stick to a total of two genetic groups (but, feel free to model more groups!).

First, create an object that will be the pedigree with genetic groups (`ggPed`).

```r
ggPed <- ggTutorial[, c("id", "dam", "sire", "is", "gen")]
naPar <- which(is.na(ggPed[, 2]))
ggPed$GG <- rep("NA", nrow(ggPed))
  # 'focal' genetic group = "foc0" and 'immigrant' = "g1"
  # obtained by pasting "foc" & "g" with immigrant status "0" or "1", respectively
  ggPed$GG[naPar] <- as.character(ggPed$is[naPar])
  ggPed$GG[ggPed$GG == "0"] <- paste0("foc", ggPed$GG[ggPed$GG == "0"])
  ggPed$GG[ggPed$GG == "1"] <- paste0("g", ggPed$GG[ggPed$GG == "1"])
ggPed[naPar, 2:3] <- ggPed[naPar, "GG"]
# Two rows need to be added for the genetic groups
## Genetic groups will be given the missing value 0 instead of parent identities
ggPed <- data.frame(id = c("foc0", "g1", as.character(ggPed$id)),
        dam = c(0, 0, as.character(ggPed$dam)),
        sire = c(0, 0, as.character(ggPed$sire)))
head(ggPed)
```

```
##     id  dam sire
## 1 foc0    0    0
## 2   g1    0    0
## 3    1 foc0 foc0
## 4    2 foc0 foc0
## 5    3 foc0 foc0
## 6    4 foc0 foc0
```

6.4.4.2 Fixed explicit genetic group effects with Q (from nadiv) Fitting genetic group effects explicitly requires the columns of **Q** to be included as separate fixed covariate regressions. The code below creates **Q** for the ggPed pedigree.

```r
Q <- ggcontrib(ggPed[, 1:3])
```

```
## Warning in numPed(pedigree): Zero in the dam column interpreted as a
## missing parent

## Warning in numPed(pedigree): Zero in the sire column interpreted as a
```

```
## missing parent
```

Note that the two warnings above (regarding zeroes being interpreted as missing dams/sires) are to be expected and should not be cause for any concern in this particular pedigree/example.

Now we add the columns of **Q** (`foc0` and `g1`) as variables in `ggTutorial` so that they can be included in a model.

```r
ggTutorial <- cbind(ggTutorial, Q)
```

```r
str(ggTutorial)
```

```
## 'data.frame':    6000 obs. of  13 variables:
##  $ id     : Factor w/ 6000 levels "1","2","3","4",..: 1 2 3 4 5 6 7 8 9 10 ...
##  $ dam    : int  NA NA NA NA NA NA NA NA NA NA ...
##  $ sire   : int  NA NA NA NA NA NA NA NA NA NA ...
##  $ parAvgU: num  NA NA NA NA NA NA NA NA NA NA ...
##  $ mendel : num  NA NA NA NA NA NA NA NA NA NA ...
##  $ u      : num  -0.393 -0.675 -0.442 -1.03 -1.913 ...
##  $ r      : num  -0.0836 0.142 1.892 -1.2982 0.4668 ...
##  $ p      : num  19.5 19.5 21.5 17.7 18.6 ...
##  $ is     : int  0 0 0 0 0 0 0 0 0 0 ...
##  $ gen    : int  1 1 1 1 1 1 1 1 1 1 ...
##  $ f      : num  0 0 0 0 0 0 0 0 0 0 ...
##  $ foc0   : num  1 1 1 1 1 1 1 1 1 1 ...
##  $ g1     : num  0 0 0 0 0 0 0 0 0 0 ...
```

An important point to make is that, because the pedigree and data for `ggTutorial` have essentially the same sizes and structures (only `ggPed` now has two extra rows for the genetic groups - but these will be dropped automatically from **Q** by `ggcontrib()`), the genetic group coefficients from **Q** can be added directly to the data with `ggTutorial <- cbind(ggTutorial, Q)` above. However, whenever a pedigree contains more individuals than the data (or in a different order), an intermediate step is required. Specifically, since the **Q** matrix contains a row for every individual in the pedigree, only the subset of rows in **Q** that correspond to phenotyped individuals in a dataset should be extracted and ordered according to the identities in the data.

Now we need to write the pedigrees and portion of the data to files in formats that **ASReml** can use. Note, **ASReml** expects pedigree columns ordered ID, Sire, Dam and we will use ‘0’ to denote missing values (instead of NA).

```
regPed <- ggTutorial[, 1:3]
regPed[is.na(regPed[, 2]), 2] <- 0
regPed[is.na(regPed[, 3]), 3] <- 0
```

```
write.table(regPed[, c(1,3,2)], "./asreml/ggReg/reg.ped",
    row.names = FALSE, quote = FALSE)
asData <- ggTutorial[, -c(2:7)]
write.table(asData, "./asreml/ggReg/ggTutorial.dat",
    row.names = FALSE, quote = FALSE)
```

The fixed regressions on `foc0` and `g1` can be combined with the standard $\mathbf{A}^{-1}$ to specify an animal model in **ASReml** (see also the “./asreml/ggReg” folder in the supporting files). Because unique solutions to the model do not exist (see discussion of estimability in *Appendix S6.2*) for the intercept, coefficient of inbreeding (f), and genetic group effects (`foc0` and `g1`), we fit the `foc0` genetic group as the last fixed effect. This ensures that the model sets `foc0` as a reference group and estimates the `g0` genetic group effect as a deviation from the mean breeding value in the `foc0` group.

```
Fixed regression for Explicit genetic groups
 id !P
 p
 is !I 0 1
 gen 15
 f
 foc0
 g1
reg.ped !ALPHA !SKIP 1 !MAKE !GIV !DIAG
ggTutorial.dat !SKIP 1
p ~ mu f g1 foc0 !r id
```

The estimate of the genetic group effects and standard errors are reported as the genetic group fixed effect regression coefficients and standard errors in the “ggReg.sln” file. These can be read back into `R`:

```
regPred <- read.table("./asreml/ggReg/ggReg.sln", header = TRUE)
```

so that we can see the fixed effect estimates and their standard errors:

```
regPred[1:4, ]
```

```
##   Model_Term Level Effect seEffect
## 1       foc0     1  0.000  0.00000
## 2         g1     1  3.117  0.08929
## 3          f     1  0.666  1.83200
## 4         mu     1 19.800  0.06579
```

Note the software has set the `foc0` group estimate to zero in this model, implying this is the reference group and the `g1` genetic group effect is the estimated deviation from this reference. The above estimate of 3.117 agrees with the expected difference between mean breeding value in the immigrant group (`3`) and the founder population (`0`) that was specified in the simulation.

The predicted breeding values (**a**) are also reported in the "ggReg.sln" file (see now `R` object `regPred`) and follow below the fixed effect estimates. Because the order of rows in **Q** matches the order of individual identities in `ggTutorial`, the total additive genetic effects (**u**) can be calculated without any further manipulation as (eqn 5 in main text):

```
reg_gHat <- matrix(regPred[1:2, "Effect"], ncol = 1)
reg_u <- Q %*% reg_gHat + regPred[-c(1:4), "Effect"]
```

Note, if more individuals are in the pedigree than the dataset and/or the identities in the pedigree and data are ordered differently, more coding is necessary to ensure that the predicted genetic effect for a particular individual is added to the correct weighted genetic group contribution. Additionally, if more random effects are included in the model, care has to be taken in order to ensure the correct random effect predictions are extracted for the breeding values (**a**). The `R` function `match()` is particularly helpful in these cases.

6.4.4.3 Fixed implicit genetic group effects with A* (from `nadiv`) Genetic group effects can be fit implicitly within the random effects when the augmented inverse relatedness matrix $\mathbf{A}^*$ has been supplied as

a list to **ASReml**. The code below creates $\mathbf{A}^*$ for the `ggPed` pedigree and saves it as a list in the format **ASReml** requires.

First, we will calculate $\mathbf{Q}$ and include it in the dataset for consistency with other versions of this data.

```
Q <- ggcontrib(ggPed[, 1:3])
```

```
## Warning in numPed(pedigree): Zero in the dam column interpreted as a
## missing parent

## Warning in numPed(pedigree): Zero in the sire column interpreted as a
## missing parent
```

Note that the two warnings above (regarding zeroes being interpreted as missing dams/sires) are to be expected and should not be cause for any concern in this particular pedigree/example.

Add the columns of $\mathbf{Q}$ (`foc0` and `g1`) as variables in `ggTutorial`.

```
ggTutorial <- cbind(ggTutorial, Q)
```

```
str(ggTutorial)
```

```
## 'data.frame':    6000 obs. of  13 variables:
##  $ id     : Factor w/ 6000 levels "1","2","3","4",..: 1 2 3 4 5 6 7 8 9 10 ...
##  $ dam    : int  NA NA NA NA NA NA NA NA NA NA ...
##  $ sire   : int  NA NA NA NA NA NA NA NA NA NA ...
##  $ parAvgU: num  NA NA NA NA NA NA NA NA NA NA ...
##  $ mendel : num  NA NA NA NA NA NA NA NA NA NA ...
##  $ u      : num  -0.393 -0.675 -0.442 -1.03 -1.913 ...
##  $ r      : num  -0.0836 0.142 1.892 -1.2982 0.4668 ...
##  $ p      : num  19.5 19.5 21.5 17.7 18.6 ...
##  $ is     : int  0 0 0 0 0 0 0 0 0 0 ...
##  $ gen    : int  1 1 1 1 1 1 1 1 1 1 ...
##  $ f      : num  0 0 0 0 0 0 0 0 0 0 ...
##  $ foc0   : num  1 1 1 1 1 1 1 1 1 1 ...
##  $ g1     : num  0 0 0 0 0 0 0 0 0 0 ...
```

An important point to make is that, because the pedigree and data for `ggTutorial` have essentially the same sizes and structures (only `ggPed` now has two extra rows for the genetic groups - but these will be dropped automatically from **Q** by `ggcontrib()`), the genetic group coefficients from **Q** can be added directly to the data with `ggTutorial <- cbind(ggTutorial, Q)` above. However, whenever a pedigree contains more individuals than the data (or in a different order), an intermediate step is required. Specifically, since the **Q** matrix contains a row for every individual in the pedigree, only the subset of rows in **Q** that correspond to phenotyped individuals in a dataset should be extracted and ordered according to the identities in the data.

Now we create the sparse matrix of $\mathbf{A}^*$ for the `ggPed` pedigree using the `makeAinv` function in the `nadiv` package.

```
listAstar <- makeAinv(ggPed, ggroups = 2, gOnTop = TRUE)$listAinv
```

In the above code, we directed `makeAinv()` to include the genetic groups on top (top-left block) of $\mathbf{A}^*$ using `gOnTop = TRUE`. This is necessary so that **ASReml** solves the equations in an order that improves model convergence (see *Appendix 6.3* and its subsections). In this case, the **ASReml** model converges. In other pedigrees, models, and/or genetic group classifications, if singularities occur then sometimes this can be overcome by adding one (or a small value) to the diagonal elements of the group-group portion of the matrix (although see discussion of this in *Appendix S5* and cautions/considerations in *Appendix 6.3.2*). The alteration is demonstrated above in the `asreml` tutorial along with cautions and interpretations (*Appendix 6.4.3.4*). The resulting list could then be given to the standalone **ASReml** program much the same as we do here (see also *Appendix 6.4.4.5*). Note, the easiest approach is to adjust the genetic group diagonals of $\mathbf{A}^*$ using the matrix returned by `makeAinv()` (i.e., object `Ainv` in the list), then convert the matrix to the list format required by **ASReml**.

Now we need to write the data (`ggTutorial`) and list of the $\mathbf{A}^*$ (`listAstar`) to a file for **ASReml**. Further, we need to write to a file all of the identity codes in the pedigree that match to the rows and columns in `listAstar`. This enables **ASReml** to match levels in the generalized inverse (row and column numbers of `listAstar`) to observations in `ggTutorial`. Critically, we need to include the "geneticGroups" attribute of `listAstar` as the first line in the file containing this list.

```
# Write the nadiv Astar ginv list
write.table(listAstar,
    file = "./asreml/ggNadivAstarFxd/nadivAstar.giv",
```

```r
    col.names = FALSE, row.names = FALSE)
  fConn <- file("./asreml/ggNadivAstarFxd/nadivAstar.giv", "r+")
  Lines <- readLines(fConn)
  # Add the !GROUPSDF *n* qualifier as the first line of the file
  ngrps <- attr(listAstar, "geneticGroups")[1]
  writeLines(c(paste0("!GROUPSDF ", ngrps), Lines), con = fConn)
  close(fConn)

# Create mapping between order of identities in listAstar and their unique codes
write.table(ggPed[, 1], file = "./asreml/ggNadivAstarFxd/nadivAstar.txt",
    col.names = FALSE, row.names = FALSE, quote = FALSE)

# Write the data
asData <- ggTutorial[, -c(2:7)]
write.table(asData, "./asreml/ggNadivAstarFxd/ggTutorial.dat",
    row.names = FALSE, quote = FALSE)
```

Now we can run the animal model in **ASReml** using the supplied general inverse matrix `listAstar` in the file "nadivAstar.giv" (see also the "./asreml/ggNadivAstarFxd" folder in the supporting files):

```
nadiv's Astar for fixed genetic group effects implicitly within the random effects
 id !A !L nadivAstar.txt
 p
 is !I 0 1
 gen 15
 f
 foc0
 g1
nadivAstar.giv
ggTutorial.dat !SKIP 1
!LAST id 2
p ~ mu f !r giv1(id)
```

Here, genetic group effects are fitted in the model using `nadiv`'s generalized inverse by including

giv1(id) in the random effects syntax statement. The line below the data file, but before the model statement, (!LAST id 2) tells **ASReml** to fit the first two equations associated with the id variable (the genetic groups) last. This is done to help the model to converge (see *Appendix 6.3.1*).

The genetic group predictions and standard errors are reported in the "ggNadivAstarFxd.sln" file. These can be read back into R:

```
nadivAstarFxdPred <- read.table("./asreml/ggNadivAstarFxd/ggNadivAstarFxd.sln", header = TRUE)
```

so that we can see the fixed effect estimates and their standard errors along with the genetic group predictions:

```
nadivAstarFxdPred[1:4, ]
```

```
##   Model_Term Level Effect seEffect
## 1          f     1  0.666  1.83200
## 2         mu     1  0.000  0.00000
## 3   giv1(id)  foc0 19.800  0.06579
## 4   giv1(id)    g1 22.910  0.06008
```

Note the software has set the intercept (mu) estimate to zero in this model (see discussion of estimability in *Appendix S6.2*), implying this is the reference to which the foc0 and g1 genetic group effects are the estimated deviations. Therefore, the predicted total additive genetic effects ($\mathbf{u}$) include the overall phenotypic mean. However, the difference between the genetic group effects 3.11 agrees with the expected difference between the mean breeding values of the immigrant group (3) and the founder population (0) that was specified in the simulation.

The predicted total additive genetic effects ($\mathbf{u}$) are also reported in the "ggNadivAstarFxd.sln" file (see now R object nadivAstarFxdPred) and follow below the genetic group predictions. Because the order of rows in $\mathbf{Q}$ matches the order of individual identities in ggTutorial, the predicted breeding values ($\mathbf{a}$) can be calculated without any further manipulation as (eqn 6 in main text):

```
AstarFxd_gHat <- matrix(nadivAstarFxdPred[3:4, "Effect"], ncol = 1)
AstarFxd_a <- nadivAstarFxdPred[-c(1:4), "Effect"] - Q %*% AstarFxd_gHat
```

Note, if more individuals are in the pedigree than the dataset and/or the identities in the pedigree and data are ordered differently, more coding is necessary to ensure that the predicted genetic effect for

a particular individual is added to the correct weighted genetic group contribution. Additionally, if more random effects are included in the model, care has to be taken in order to ensure the correct random effect predictions are extracted for the total additive genetic effects ($\mathbf{u}$) and used to calculate breeding values ($\mathbf{a}$). The `R` function `match()` is particularly helpful in these cases.

6.4.4.4 Fixed implicit genetic group effects with A* (from ASReml) **ASReml** can fit genetic group effects implicitly within the random effects using its own function to construct $\mathbf{A}^*$. First, we construct the data and pedigree files in `R`.

Calculate $\mathbf{Q}$ and include it in the dataset for consistency with other versions of this data.

```
Q <- ggcontrib(ggPed[, 1:3])
```

```
## Warning in numPed(pedigree): Zero in the dam column interpreted as a
## missing parent
```

```
## Warning in numPed(pedigree): Zero in the sire column interpreted as a
## missing parent
```

Note that the two warnings above (regarding zeroes being interpreted as missing dams/sires) are to be expected and should not be cause for any concern in this particular pedigree/example.

Add the columns of $\mathbf{Q}$ (`foc0` and `g1`) as variables in `ggTutorial`.

```
ggTutorial <- cbind(ggTutorial, Q)
```

```
str(ggTutorial)
```

```
## 'data.frame':    6000 obs. of  13 variables:
##  $ id     : Factor w/ 6000 levels "1","2","3","4",..: 1 2 3 4 5 6 7 8 9 10 ...
##  $ dam    : int  NA NA NA NA NA NA NA NA NA NA ...
##  $ sire   : int  NA NA NA NA NA NA NA NA NA NA ...
##  $ parAvgU: num  NA NA NA NA NA NA NA NA NA NA ...
##  $ mendel : num  NA NA NA NA NA NA NA NA NA NA ...
##  $ u      : num  -0.393 -0.675 -0.442 -1.03 -1.913 ...
##  $ r      : num  -0.0836 0.142 1.892 -1.2982 0.4668 ...
```

```
## $ p     : num  19.5 19.5 21.5 17.7 18.6 ...
## $ is    : int  0 0 0 0 0 0 0 0 0 0 ...
## $ gen   : int  1 1 1 1 1 1 1 1 1 1 ...
## $ f     : num  0 0 0 0 0 0 0 0 0 0 ...
## $ foc0  : num  1 1 1 1 1 1 1 1 1 1 ...
## $ g1    : num  0 0 0 0 0 0 0 0 0 0 ...
```

An important point to make is that, because the pedigree and data for `ggTutorial` have essentially the same sizes and structures (only `ggPed` now has two extra rows for the genetic groups - but these will be dropped automatically from **Q** by `ggcontrib()`), the genetic group coefficients from **Q** can be added directly to the data with `ggTutorial <- cbind(ggTutorial, Q)` above. However, whenever a pedigree contains more individuals than the data (or in a different order), an intermediate step is required. Specifically, since the **Q** matrix contains a row for every individual in the pedigree, only the subset of rows in **Q** that correspond to phenotyped individuals in a dataset should be extracted and ordered according to the identities in the data.

Write the pedigree containing genetic groups and the relevant portion of the data to files in formats that **ASReml** can use. Note, **ASReml** expects pedigree columns ordered ID, Sire, Dam.

```r
# Write the pedigree
write.table(ggPed[, c(1,3,2)], "./asreml/ggAsremlAstarFxd/asremlGG.ped",
    row.names = FALSE, quote = FALSE)

# Write the data
asData <- ggTutorial[, -c(2:7)]
write.table(asData, "./asreml/ggAsremlAstarFxd/ggTutorial.dat",
    row.names = FALSE, quote = FALSE)
```

The model is specified in **ASReml** (see also the "./asreml/ggAsremlAstarFxd" folder in the supporting files):

```
ASReml's Astar for fixed genetic groups implicitly within the random effects
 id !P
 p
 is !I 0 1
```

```
 gen 15
 f
 foc0
 g1
asremlGG.ped !ALPHA !SKIP 1 !MAKE !GIV !DIAG !GROUPS 2
ggTutorial.dat !SKIP 1
!LAST id 2
p ~ mu f !r id
```

Here, genetic groups are specified as the top two lines of the pedigree file `asremlGG.ped` using `!GROUPS 2` as an additional qualifier to the pedigree file line. **ASReml** then creates $\mathbf{A}^*$ from the pedigree and saves this as a list in the file "ggAsremlAstarFxd_A.giv" (when the `!GIV` qualifier is included in the pedigree line). The line below the data file, but before the model statement, (`!LAST id 2`) tells **ASReml** to fit the first two equations associated with the `id` variable (the genetic groups) last. This is done to help the model to converge (see *Appendix 6.3.1*).

The genetic group predictions and standard errors are reported in the "ggAsremlAstarFxd.sln" file. These can be read back into `R`:

```
asremlAstarFxdPred <- read.table("./asreml/ggAsremlAstarFxd/ggAsremlAstarFxd.sln", header = TRUE)
```

so that we can see the fixed effect estimates and their standard errors along with the genetic group predictions:

```
asremlAstarFxdPred[1:4, ]
```

```
##   Model_Term Level Effect seEffect
## 1          f     1  0.666  1.83200
## 2         mu     1  0.000  0.00000
## 3         id  foc0 19.800  0.06579
## 4         id    g1 22.910  0.06008
```

Note the software has set the intercept (`mu`) estimate to zero in this model (see discussion of estimability in *Appendix S6.2*), implying this is the reference to which the `foc0` and `g1` genetic group effects are the estimated deviations. Therefore, the predicted total additive genetic effects ($\mathbf{u}$) include the overall phenotypic

mean. However, the difference between the genetic group effects 3.11 agrees with the expected difference between the mean breeding values of the immigrant group (3) and the founder population (0) that was specified in the simulation.

The predicted total additive genetic effects (**u**) are also reported in the "ggAsremlAstarFxd.sln" file (see now `R` object `asremlAstarFxdPred`) and follow below the genetic group predictions. Because the order of rows in **Q** matches the order of individual identities in `ggTutorial`, the predicted breeding values (**a**) can be calculated without any further manipulation as (eqn 6 in main text):

```
AstarFxd_gHat <- matrix(asremlAstarFxdPred[3:4, "Effect"], ncol = 1)
AstarFxd_a <- asremlAstarFxdPred[-c(1:4), "Effect"] - Q %*% AstarFxd_gHat
```

Note, if more individuals are in the pedigree than the dataset and/or the identities in the pedigree and data are ordered differently, more coding is necessary to ensure that the predicted genetic effect for a particular individual is added to the correct weighted genetic group contribution. Additionally, if more random effects are included in the model, care has to be taken in order to ensure the correct random effect predictions are extracted for the total additive genetic effects (**u**) and used to calculate breeding values (**a**). The `R` function `match()` is particularly helpful in these cases.

6.4.4.5 Random implicit genetic group effects with A* (from ASReml) Fitting random effects of genetic groups implicity within the random effects of an animal model requires a modified augmented inverse relatendess matrix ($\mathbf{A}^*$) to be created. This can be accomplished in `R` with `nadiv` such that the object is then passed to **ASReml** or the built-in functions of **ASReml** can do this while fitting the model. Creating the modified matrix in `R` using `nadiv` has been demonstrated in *Appendix 6.4.3.4* and including such a matrix in an **ASReml** analysis is demonstrated in *Appendix 6.4.4.3*. Here, we demonstrate how to use **ASReml** to create a modified $\mathbf{A}^*$ for the `ggPed` pedigree that has the necessary alterations (see discussion of this in *Appendix S5* and cautions/considerations in *Appendix 6.3.2*) so the model interprets the genetic groups as random effects and fit the model all in a single execution of **ASReml**.

First, we construct the data and pedigree files in `R`. Calculate **Q** and include it in the dataset for consistency with other versions of this data.

```
Q <- ggcontrib(ggPed[, 1:3])
```

```
## Warning in numPed(pedigree): Zero in the dam column interpreted as a
## missing parent
```

```
## Warning in numPed(pedigree): Zero in the sire column interpreted as a
## missing parent
```

Note that the two warnings above (regarding zeroes being interpreted as missing dams/sires) are to be expected and should not be cause for any concern in this particular pedigree/example.

Add the columns of **Q** (`foc0` and `g1`) as variables in `ggTutorial`.

```
ggTutorial <- cbind(ggTutorial, Q)
```

```
str(ggTutorial)
```

```
## 'data.frame':    6000 obs. of  13 variables:
##  $ id     : Factor w/ 6000 levels "1","2","3","4",..: 1 2 3 4 5 6 7 8 9 10 ...
##  $ dam    : int  NA NA NA NA NA NA NA NA NA NA ...
##  $ sire   : int  NA NA NA NA NA NA NA NA NA NA ...
##  $ parAvgU: num  NA NA NA NA NA NA NA NA NA NA ...
##  $ mendel : num  NA NA NA NA NA NA NA NA NA NA ...
##  $ u      : num  -0.393 -0.675 -0.442 -1.03 -1.913 ...
##  $ r      : num  -0.0836 0.142 1.892 -1.2982 0.4668 ...
##  $ p      : num  19.5 19.5 21.5 17.7 18.6 ...
##  $ is     : int  0 0 0 0 0 0 0 0 0 0 ...
##  $ gen    : int  1 1 1 1 1 1 1 1 1 1 ...
##  $ f      : num  0 0 0 0 0 0 0 0 0 0 ...
##  $ foc0   : num  1 1 1 1 1 1 1 1 1 1 ...
##  $ g1     : num  0 0 0 0 0 0 0 0 0 0 ...
```

An important point to make is that, because the pedigree and data for `ggTutorial` have essentially the same sizes and structures (only `ggPed` now has two extra rows for the genetic groups - but these will be dropped automatically from **Q** by `ggcontrib()`), the genetic group coefficients from **Q** can be added directly to the data with `ggTutorial <- cbind(ggTutorial, Q)` above. However, whenever a pedigree contains more individuals than the data (or in a different order), an intermediate step is required. Specifically, since the **Q** matrix contains a row for every individual in the pedigree, only the subset of rows in **Q** that correspond to phenotyped individuals in a dataset should be extracted and ordered according to the identities in the data.

Write the pedigree containing genetic groups and the relevant portion of the data to files in formats that **ASReml** can use. Note, **ASReml** expects pedigree columns ordered ID, Sire, Dam.

```
# Write the pedigree
write.table(ggPed[, c(1,3,2)], "./asreml/ggAsremlRan/asremlGG.ped",
    row.names = FALSE, quote = FALSE)

# Write the data
asData <- ggTutorial[, -c(2:7)]
write.table(asData, "./asreml/ggAsremlRan/ggTutorial.dat",
    row.names = FALSE, quote = FALSE)
```

The model is specified in **ASReml** (see also the "./asreml/ggAsremlRan" folder in the supporting files):

```
ASReml's Astar for random genetic groups implicitly within the random effects
 id !P
 p
 is !I 0 1
 gen 15
 f
 foc0
 g1
asremlGG.ped !ALPHA !SKIP 1 !MAKE !GIV !DIAG !GROUPS 2 !GOFFSET 0.1
ggTutorial.dat !SKIP 1
!LAST id 2
p ~ mu f !r id
```

Here, genetic groups are specified as the top two lines of the pedigree file `asremlGG.ped` using `!GROUPS 2` as an additional qualifier to the pedigree file line. Further, the `!GOFFSET 0.1` qualifier to the pedigree line instructs **ASReml** to add `0.1` to the diagonal elements of the group-group equations in $\mathbf{A}^*$ (see discussion of this in *Appendix S5*, cautions/considerations in *Appendix 6.3.2*, and other examples in *Appendix 6.4.2.3* and *Appendix 6.4.3.4*). As a result of this alteration, the genetic group effects are conceptually random effects in the model. The addition of 0.1 to the diagonal element of the $\mathbf{A}^*$ constrains the variance in genetic group effects to be one tenth the additive genetic variance estimated by the model. A value of 0.1 may bias estimates

of additive genetic variance in models of other datasets. Therefore, we advise testing values other than 0.1 (e.g., 1 or 10) and comparing estimates from these alternative models. Note that a value of 1 constrains the variance in genetic group effects to be equal to the additive genetic variance estimated by the model.

ASReml then creates $\mathbf{A}^*$ from the pedigree and saves this as a list in the file "ggAsremlRan_A.giv" (when the `!GIV` qualifier is included in the pedigree line). The line below the data file, but before the model statement, (`!LAST id 2`) tells **ASReml** to fit the first two equations associated with the `id` variable (the genetic groups) last. This is done to help the model to converge (see *Appendix 6.3.1*).

The genetic group predictions and standard errors are reported in the "ggAsremlRan.sln" file. These can be read back into `R`:

```
asremlRanPred <- read.table("./asreml/ggAsremlRan/ggAsremlRan.sln", header = TRUE)
```

so that we can see the fixed effect estimates and their standard errors along with the genetic group predictions:

```
asremlRanPred[1:4, ]
```

```
##   Model_Term Level  Effect seEffect
## 1          f     1  0.6656    1.832
## 2         mu     1 21.3600    2.321
## 3         id  foc0 -1.5580    2.321
## 4         id    g1  1.5580    2.321
```

Note the intercept in this model (`mu`) is estimable and so the model returns this estimate along with predicting both of the genetic group effects. The genetic group predictions, themselves, are random effects and thus are deviations from their expected value of 0. The difference between the genetic groups 3.116 agrees with the expected difference between the mean breeding values of the immigrant group (3) and the founder population (0) that was specified in the simulation.

The predicted total additive genetic effects ($\mathbf{u}$) are also reported in the "ggAsremlRan.sln" file (see now `R` object `asremlRanPred`) and follow below the genetic group predictions. Here, the prediced total additive genetic effects include the genetic group effects expressed as deviations from 0. Because the order of rows in $\mathbf{Q}$ matches the order of individual identities in `ggTutorial`, the predicted breeding values ($\mathbf{a}$) can be calculated without any further manipulation as (eqn 6 in main text):

```
AstarRan_gHat <- matrix(asremlRanPred[3:4, "Effect"], ncol = 1)
AstarRan_a <- asremlRanPred[-c(1:4), "Effect"] - Q %*% AstarRan_gHat
```

Note, if more individuals are in the pedigree than the dataset and/or the identities in the pedigree and data are ordered differently, more coding is necessary to ensure that the predicted genetic effect for a particular individual is added to the correct weighted genetic group contribution. Additionally, if more random effects are included in the model, care has to be taken in order to ensure the correct random effect predictions are extracted for the total additive genetic effects ($\mathbf{u}$) and used to calculate breeding values ($\mathbf{a}$). The `R` function `match()` is particularly helpful in these cases.

6.4.5 WOMBAT

Here are three approaches to fit genetic groups in a **WOMBAT** analysis. All three require some preparation using the `nadiv` functions in `R`. However, only the explicit regression on the genetic group covariate includes genetic groups as fixed effects. The second and third examples below include genetic groups as random effects in the model. It should be noted that **WOMBAT** sets up the mixed model slightly differently than the previous software programs. Notably, **WOMBAT** fits response variables and covariates as deviations from the mean response and mean of each covariate, respectively. Thus, regression coefficients are equivalent, but have slightly different estimates/interpretations from the estimates in the above tutorials. The preparation of the `ggTutorial` data and requisite outputs from `nadiv` in `R` will first be demonstrated before detailing the code used in the **WOMBAT** model fitting. However, the basic files necessary to fit the **WOMBAT** models without any preparation in `R` are available in the accompanying supporting files on Dryad (Wolak & Reid 2016). The content of these files is:

- `ggTutorial.d` contains the re-coded `ggTutorial` data. A value of 10000 has been added to the `id` to allow unique integer codes for the genetic groups (**WOMBAT** requires integer identities). This file contains space separated columns from a subset of the columns in the original `data.frame` in the following order: `id`, `p`, `is`, `gen`, `f`, `foc0`, and `g1`. This is needed for all three types of models fitted.
- `wombatPed.d` contains the pedigree and is analogous to the first three columns of `ggTutorial`
- `ggReg.par`, `ggNadivAstarRan.par`, and `ggWombatRan.par` are included within each folder and each specifies a separate model to run.

- `id.gin` contains an inverse covariance matrix, in list format. This is either the `nadiv` created $\mathbf{A}^*$ or $\mathbf{A}^{-1}$, depending on the analysis.

- `id.codes` contains the mapping of running integers to the particular name of individuals in `ggTutorial.d`. For **WOMBAT**'s implementation of a genetic group analysis, this file must contain an extra column (dummy column in these analyses) that contains an integer code specifying if a genotype is available, as well as extra columns with the $\mathbf{Q}$ matrix (which must be constructed within `nadiv` as there is no function to do this in **WOMBAT**).

To prepare the data for an animal model, all that is necessary is to calculate the coefficients of inbreeding (f; to minimize bias from inbreeding depression, see the last paragraph of *Appendix 6.4.1*). This calculation can be done using the `makeAinv()` function in the `nadiv` package.

```
library(nadiv)
```

```
ggTutorial$f <- makeAinv(ggTutorial[, 1:3])$f
```

6.4.5.1 Preparing a pedigree with genetic groups Calculating the matrix of genetic group contributions ($\mathbf{Q}$) and the augmented inverse relatedness matrix ($\mathbf{A}^*$) requires a pedigree that has genetic groups indicated instead of missing values for parents. In the `ggTutorial` data, we will assign all individuals with unknown parents in the first generation phantom parents from one genetic group (`foc0`). All individuals in subsequent generations that have unknown parents will be assigned phantom parents from a second genetic group (`g1`). Alternatively, this second group could be further divided into genetic groups based on generation. However, the data were simulated such that every immigrant has the same expected mean breeding value, regardless of the generation in which it was born. Therefore, we will stick to a total of two genetic groups (but, feel free to model more than 2 groups!).

Create an object that will be the pedigree with genetic groups (`ggPed`).

```
ggPed <- ggTutorial[, c("id", "dam", "sire", "is", "gen")]
naPar <- which(is.na(ggPed[, 2]))
ggPed$GG <- rep("NA", nrow(ggPed))
  # 'focal' genetic group = "foc0" and 'immigrant' = "g1"
  # obtained by pasting "foc" & "g" with immigrant status "0" or "1", respectively
  ggPed$GG[naPar] <- as.character(ggPed$is[naPar])
```

```
  ggPed$GG[ggPed$GG == "0"] <- paste0("foc", ggPed$GG[ggPed$GG == "0"])
  ggPed$GG[ggPed$GG == "1"] <- paste0("g", ggPed$GG[ggPed$GG == "1"])
ggPed[naPar, 2:3] <- ggPed[naPar, "GG"]
```

Note, the approach and format of the pedigree below is different from the `Q1988sub` pedigree (*Appendix 6.1*) - mostly because the format below makes it easier to specify genetic groups in a pedigree for this particular case, but partly to illustrate the flexibility of `nadiv` functions. To be more specific, the `Q1988sub` pedigree contained two extra rows for the two genetic groups. The `ggroups` argument to `makeAinv()` supplied an integer indicating how many rows at the begining of the pedigree contained genetic groups and not individuals. However, below no extra rows are added to the pedigree and the character vector given to the `ggroups` argument in both `ggcontrib()` and `makeAinv()` specifies the unique genetic groups.

6.4.5.2 Fixed explicit genetic group effects with Q (from `nadiv`) Fitting genetic group effects explicitly requires the columns of **Q** to be included as separate fixed covariate regressions. The code below creates **Q** for the `ggPed` pedigree and adds the columns (`foc0` and `g1`) as variables in `ggTutorial` so that they can be included in a model.

```
Q <- ggcontrib(ggPed[, 1:3], ggroups = c("foc0", "g1"))
```

```
ggTutorial <- cbind(ggTutorial, Q)
```

```
str(ggTutorial)
```

```
## 'data.frame':    6000 obs. of  13 variables:
##  $ id     : Factor w/ 6000 levels "1","2","3","4",..: 1 2 3 4 5 6 7 8 9 10 ...
##  $ dam    : int  NA NA NA NA NA NA NA NA NA NA ...
##  $ sire   : int  NA NA NA NA NA NA NA NA NA NA ...
##  $ parAvgU: num  NA NA NA NA NA NA NA NA NA NA ...
##  $ mendel : num  NA NA NA NA NA NA NA NA NA NA ...
##  $ u      : num  -0.393 -0.675 -0.442 -1.03 -1.913 ...
##  $ r      : num  -0.0836 0.142 1.892 -1.2982 0.4668 ...
##  $ p      : num  19.5 19.5 21.5 17.7 18.6 ...
##  $ is     : int  0 0 0 0 0 0 0 0 0 0 ...
##  $ gen    : int  1 1 1 1 1 1 1 1 1 1 ...
```

```
## $ f      : num  0 0 0 0 0 0 0 0 0 0 ...
## $ foc0   : num  1 1 1 1 1 1 1 1 1 1 ...
## $ g1     : num  0 0 0 0 0 0 0 0 0 0 ...
```

An important point to make is that, because the pedigree and data for `ggTutorial` have essentially the same sizes and structures, the genetic group coefficients from **Q** can be added directly to the data with `ggTutorial <- cbind(ggTutorial, Q)` above. However, whenever a pedigree contains more individuals than the data (or in a different order), an intermediate step is required. Specifically, since the **Q** matrix contains a row for every individual in the pedigree, only the subset of rows in **Q** that correspond to phenotyped individuals in a dataset should be extracted and ordered according to the identities in the data.

Now we need to write the pedigree and portion of the data to files in formats that **WOMBAT** can use. Also, for consistency with analyses below, we will add a constant value of 10000 to the integer `ids`.

```
IDaddDF <- ggTutorial[, -c(4:7)]
IDaddDF[, 1] <- as.integer(as.character(IDaddDF[, 1])) + 10000
IDaddDF[, 2] <- as.integer(as.character(IDaddDF[, 2])) + 10000
IDaddDF[, 3] <- as.integer(as.character(IDaddDF[, 3])) + 10000

IDaddPed <- IDaddDF[, 1:3]
IDaddPed[is.na(IDaddPed[, 2]), 2] <- 0
IDaddPed[is.na(IDaddPed[, 3]), 3] <- 0
```

```
write.table(IDaddDF[, -c(2:3)], "./wombat/ggTutorial.d", col.names = FALSE,
    row.names = FALSE)
write.table(IDaddPed, "./wombat/wombatPed.d", col.names = FALSE,
    row.names = FALSE)
```

The fixed regressions on `foc0` and `g1` can be combined with the standard $\mathbf{A}^{-1}$ to specify an animal model in **WOMBAT** (see also the "./wombat/ggReg" folder in the supporting files). Because unique solutions to the model do not exist (see discussion of estimability in *Appendix S6.2*) for the coefficient of inbreeding (f) and genetic group effects (`foc0` and `g1`), we fit the `foc0` genetic group as the last fixed effect. This ensures that the model sets `foc0` as a reference group and estimates the `g0` genetic group effect as a deviation from the mean breeding value in the `foc0` group.

```
COM Explicit genetic groups fitted as Fixed regressions
PED ../wombatPed.d
DAT ../ggTutorial.d
  id 6000
  p
  is 2
  gen 15
  f
  foc0
  g1
end
ANAL UNI
MODEL
 COV f(1)
 COV g1(1)
 COV foc0(1)
 RAN id NRM
 TR p
END
VAR id 1
 0.5
VAR error 1
 0.5
SPECIAL
  COVZER f(1) FIT
  COVZER foc0(1) FIT
  COVZER g1(1) FIT
END
```

Note the last `SPECIAL` section, which is necessary because both the coefficient of inbreeding (f) and the genetic group contributions (`foc0` and `g1`) have values of zero that are meaningful (i.e., these zeroes do not indicate missing values).

Estimates of the genetic group effects are reported in the "./wombat/ggReg/FixSolutions.out" file. Note the software has set the `foc0` group estimate to zero in this model, implying this is the reference group and the `g1` genetic group effect is the estimated deviation. The estimate of 3.098 agrees with the expected difference between mean breeding value in the immigrant group (`3`) and the founder population (`0`) that was specified in the simulation.

The predicted breeding values (**a**) are also reported in the "RnSoln_id.dat" file.

6.4.5.3 Random implicit genetic group effects with A* (from `nadiv`) Fitting genetic group effects implicitly within the random effects requires the list format of a modified $\mathbf{A}^*$ to be supplied to **WOMBAT**. Currently, there is no option to incorporate the number of genetic groups fitted implicitly when the software calculates the residual degrees of freedom. Residual degrees of freedom that incorporate the number of genetic groups are needed to calculate the residual variance and the log-likelihood for a model in which genetic groups are considered fixed effects (e.g., see how the residual degrees of freedom are adjusted in an `asreml` analysis in *Appendix 6.4.3.4*). However, the number of genetic groups are not considered in these same calculations when genetic groups are considered random effects. Therefore, we demonstrate fitting genetic groups implicitly when groups are treated as random effects.

Fitting random effects of genetic groups implicity within the random effects of an animal model requires the augmented inverse relatendess matrix ($\mathbf{A}^*$) to be supplied as a list. First, we create the sparse matrix of $\mathbf{A}^*$ for the `ggPed` pedigree using the `makeAinv` function in the `nadiv` package in `R`, then make the necessary alterations (see discussion of this in *Appendix S5* and cautions/considerations in *Appendix 6.3.2*) so the model interprets the genetic groups as random effects, convert the sparse matrix to a list, and fit the model.

The code below creates $\mathbf{A}^*$ for the `ggPed` pedigree, the data file, and `id.codes` so that the genetic group and identities in $\mathbf{A}^*$ can be associated with identities in the data file. We will also calculate $\mathbf{Q}$ and include it in the dataset for consistency with other versions of this data.

```
Q <- ggcontrib(ggPed[, 1:3], ggroups = c("foc0", "g1"))
ggTutorial <- cbind(ggTutorial, Q)
```

An important point to make is that, because the pedigree and data for `ggTutorial` have essentially the same sizes and structures (only `ggPed` now has two extra rows for the genetic groups - but these will be dropped automatically from $\mathbf{Q}$ by `ggcontrib()`), the genetic group coefficients from $\mathbf{Q}$ can be added directly to the data with `ggTutorial <- cbind(ggTutorial, Q)` above. However, whenever a pedigree contains

more individuals than the data (or in a different order), an intermediate step is required. Specifically, since the **Q** matrix contains a row for every individual in the pedigree, only the subset of rows in **Q** that correspond to phenotyped individuals in a dataset should be extracted and ordered according to the identities in the data.

Next create $\mathbf{A}^*$:

```
Astar <- makeAinv(ggPed[, 1:3], ggroups = c("foc0", "g1"), gOnTop = TRUE)$Ainv
```

Next we add a small value to the diagonal elements of the group-group portion of the matrix. As a result of this alteration, the genetic group effects are conceptually random effects in the model. Below, we add 0.1 to the diagonal element of the $\mathbf{A}^*$ which will constrain the variance in genetic group effects to be one tenth the additive genetic variance estimated by the model. A value of 0.1 may bias estimates of additive genetic variance in models of other datasets. Therefore, we advise testing values other than 0.1 (e.g., 1 or 10) and comparing estimates from these alternative models. Note that a value of 1 constrains the variance in genetic group effects to be equal to the additive genetic variance estimated by the model.

The easiest approach is to adjust the genetic group diagonals of $\mathbf{A}^*$, and this is why we stored the matrix returned by `makeAinv()` above (i.e., object `Ainv` in the list). Once the additions are made, we can then convert the matrix to the list format required by `asreml`.

```
AstarAdd <- Astar
(ggrows <- match(c("foc0", "g1"), dimnames(AstarAdd)[[1]]))
```

```
## [1] 1 2
```

```
diag(AstarAdd)[ggrows] <- diag(AstarAdd)[ggrows] + 0.1
Astar[ggrows, ggrows]
```

```
## 2 x 2 sparse Matrix of class "dgCMatrix"
##
## foc0 400   .
## g1     . 520
```

```r
AstarAdd[ggrows, ggrows]
```

```
## 2 x 2 sparse Matrix of class "dgCMatrix"
##
## foc0 400.1   .
## g1     .   520.1
```

```r
# convert to the list format using `nadiv::sm2list()`
listAstarAdd <- sm2list(AstarAdd, rownames = rownames(AstarAdd))
```

Now we need to write the data, modified $\mathbf{A}^*$ list, and identity codes to files in formats that **WOMBAT** can use. Also, for consistency with analyses below, we will add a constant value of 10000 to the integer `id`s.

```r
IDaddDF <- ggTutorial[, -c(2:7)]
IDaddDF[, 1] <- as.integer(as.character(IDaddDF[, 1])) + 10000
write.table(IDaddDF[, -c(2:3)], "./wombat/ggTutorial.d", col.names = FALSE,
    row.names = FALSE)

# Write the general inverse of A* to file, note the re-ordering of first two columns
## First, calculate the log determinant (see note below)
AstarAddLogdet <- -1 * determinant(AstarAdd, logarithm = TRUE)$modulus[1]
write.table(listAstarAdd[, c(2,1,3)], "./wombat/ggNadivAstarRan/id.gin",
    col.names = FALSE, row.names = FALSE)
  fConn <- file("./wombat/ggNadivAstarRan/id.gin", "r+")
  Lines <- readLines(fConn)
# Add the log-determinant as the first line of the file
  writeLines(c(AstarAddLogdet, Lines), con = fConn)
  close(fConn)

# Create mapping between order of identities and their unique codes
## including 2 genetic groups
id.codes <- cbind(seq(1, max(listAstarAdd[, 1:2]), 1), c(1, 2, 10000 + seq(nrow(ggTutorial))))
write.table(id.codes, "./wombat/ggNadivAstarRan/id.codes", col.names = FALSE,
    row.names = FALSE)
```

Note above that we added a step to calculate the log-determinant of the modified $\mathbf{A}^*$. **WOMBAT** requires this as the first line of a `.gin` (generalized inverse) file (note this is the log-determinant of the non-inverse matrix, which can be calculated from the inverse).

The animal model in **WOMBAT** using the supplied general inverse matrix $\mathbf{A}^*$ (see also the "./wombat/ggNadivAstarRan" folder in the supporting files):

```
COM Random genetic groups fitted implicitly using Astar from nadiv
DAT ../ggTutorial.d
  id 6002
  p
  is 2
  gen 15
  f
  foc0
  g1
end
ANAL UNI
MODEL
 COV  f(1)
 RAN  id    GIN
 TR   p
END
VAR id 1
 0.5
VAR error 1
 0.5
SPECIAL
  COVZER f(1) FIT
END
```

Note the last `SPECIAL` section, which is necessary because the coefficient of inbreeding (f) has values of zero that are meaningful (i.e., these zeroes do not indicate missing values).

The genetic group effect predictions are reported as the first two values in the "./wom-

bat/ggNadivAstarRan/RnSoln_id.dat" file. The genetic group predictions are random effects and thus are deviations from their expected value of zero. The difference between the genetic groups 3.117 agrees with the expected difference between the mean breeding values of the immigrant group (3) and the founder population (0) that was specified in the simulation.

The predicted total additive genetic effects ($\mathbf{u}$) are also reported in the "./wombat/ggNadivAstarRan/RnSoln_id.dat" file. Here, the prediced total additive genetic effects include the genetic group effects expressed as deviations from 0.

6.4.5.4 WOMBAT's genetic groups (Random group effects with Q from `nadiv`) Fitting genetic group effects using **WOMBAT**'s own functionality still requires calculating and supplying $\mathbf{Q}$. Although $\mathbf{Q}$ is supplied, genetic groups are included implicitly within the random effects. Further, the genetic group effects themselves are treated as random effects with a variance among group effects that is estimated by the model.

WOMBAT does not provide a function to calculate $\mathbf{Q}$, so we calculate it using `nadiv`'s `ggcontrib()` function and provide $\mathbf{A^{-1}}$ as a generalized inverse. **WOMBAT** can be used to create $\mathbf{A^{-1}}$, but here we will use the `makeAinv()` function in `nadiv`. For more details, see example 18 supplied with **WOMBAT**, particularly the pdf file entitled "RNote_WOMBATS2Step.pdf" that is included within the Example 18 folder and the "wombat.par" file in the folder "~/Example18/C".

The code below creates $\mathbf{A^{-1}}$ for the `ggPed` pedigree and creates the data file and `id.codes` that contains $\mathbf{Q}$ (we also include the columns of $\mathbf{Q}$ in the data file for consistency with other versions of this data set).

```
Q <- ggcontrib(ggPed[, 1:3], ggroups = c("foc0", "g1"))
```

```
ggTutorial <- cbind(ggTutorial, Q)
```

```
AinvOut <- makeAinv(ggTutorial[, 1:3], det = TRUE)
listAstar <- sm2list(AinvOut$Ainv)
AinvLogdet <- AinvOut$logDet
```

Note above we told `makeAinv()` to calculate the log-determinant. **WOMBAT** requires this as the first line of a `.gin` (generalized inverse) file (note this is the log-determinant of the non-inverse matrix $\mathbf{A}$, which `nadiv` calculates when forming the inverse - here $\mathbf{A^{-1}}$). Now we need to write the data, $\mathbf{A^{-1}}$ list, and identity codes to files in formats that **WOMBAT** can use. Also, for consistency with other analyses, we will add a constant value of 10000 to the integer `ids`.

```r
IDaddDF <- ggTutorial[, -c(2:7)]
IDaddDF[, 1] <- as.integer(as.character(IDaddDF[, 1])) + 10000
write.table(IDaddDF[, -c(2:3)], "./wombat/ggTutorial.d", col.names = FALSE,
    row.names = FALSE)

# Write the general inverse of A^-1 to file, note the re-ordering of first two columns
write.table(listAinv[, c(2,1,3)], "./wombat/ggWombatRan/id.gin",
    col.names = FALSE, row.names = FALSE)
  fConn <- file("./wombat/ggWombatRan/id.gin", "r+")
  Lines <- readLines(fConn)
# Add the log-determinant as the first line of the file
  writeLines(c(AinvLogdet, Lines), con = fConn)
  close(fConn)

# Create mapping between order of identities and their unique codes
## including 2 genetic groups
## also add "dummy" column to indicate no genotypes available (1)
## Add Q matrix as final columns
id.codes <- cbind(seq(1, 6000), 10000 + seq(nrow(ggTutorial)), rep(1, 6000), round(10000*Q, 0))
write.table(id.codes, "./wombat/ggWombatRan/id.codes", col.names = FALSE,
    row.names = FALSE)
```

Note that **WOMBAT** wants the genetic group contributions in the `id.codes` file as an integer. Therefore, we have multiplied **Q** by a large number (10000 used above) and then rounded these to the nearest whole number. This scaling factor of 10000 will need to be indicated in the parameter file ("ggWombatRan.par"). The animal model in **WOMBAT** is then (see also the "./wombat/ggWombatRan" folder in the supporting files):

```
COM WOMBATs genetic groups (random genetic group effects with Q from `nadiv`)
DAT ../ggTutorial.d
  id 6000
  p
  is 2
```

```
  gen 15
  f
  foc0
  g1
end
ANAL UNI
MODEL
  COV   f(1)
  SUBJ  id
  RAN   id     GIN
  RAN   ggrps  IDE
  TR    p
END
VAR id 1
 0.5
VAR ggrps 1
 1.0
VAR error 1
 0.5
SPECIAL
  COVZER f(1) FIT
END
SPECIAL
  GENGROUPS ggrps 2 10000
END
```

Here, genetic groups are specified as their own random effect in the model as `ggrps` with a diagonal covariance matrix specified (`IDE`). Thus, genetic group effects are conceptually treated as random effects (*Appendix S1*) in the model. Note the `SPECIAL` sections, which are necessary because the coefficient of inbreeding (f) has values of zero that are meaningful (i.e., these zeroes do not indicate missing values) and for fitting genetic groups. The genetic group `SPECIAL` section indicates the scaling factor by which we multiplied elements in $\mathbf{Q}$ that were supplied in the file "./wombat/ggWombatRan/id.codes".

The variance estimates in "./wombat/ggWombatRan/SumEstimates.out" match the simulated values

for the `id` and residual terms. The estimated variance for the genetic group term (`ggrps`) is approximately `2`. All other simulated values are recovered in this analysis (model estimates match simulated values).

The genetic group effect predictions are reported in the "./wombat/ggWombatRan/RnSoln_ggrps.dat" file. The genetic group predictions are random effects and thus are deviations from their expected value of zero. The difference between the genetic groups 3.111 agrees with the expected difference between the mean breeding values of the immigrant group (`3`) and the founder population (`0`) that was specified in the simulation.

The predicted total additive genetic effects ($\mathbf{u}$) are reported in the "./wombat/ggWombatRan/RnSoln_id.dat" file. Here, the prediced total additive genetic effects include the genetic group effects expressed as deviations from 0.

References: Appendix S6

Butler, D.G., Cullis, B.R., Gilmour, A.R. & Gogel, B.J. (2009) asreml: asreml() fits the linear mixed model. R package version 3.0. www.vsni.co.uk.

Davis, T.A. (2006) Direct Methods for Sparse Linear Systems. SIAM, Philadelphia.

Gilmour, A.R. (2005) Developments in utilizing pedigrees in genetic analysis within ASReml. Proceedings of the Association for the Advancement of Animal Breeding and Genetics, pp. 290–293.

Gilmour, A.R. (2010) Handling non positive definite relationship matrices in mixed models. 9th World Congress on Genetics Applied to Livestock Production, pp. 1–4. Leipzig, Germany.

Gilmour, A.R., Gogel, B.J., Cullis, B.R., Welham, S.J. & Thompson, R. (2014) ASReml 4.1 user guide.

Hadfield, J.D. (2010) MCMC methods for multi-response generalized linear mixed models: the MCMCglmm R package. Journal of Statistical Software, 33, 1–22.

Hadfield, J.D., Wilson, A.J., Garant, D., Sheldon, B.C. & Kruuk, L.E.B. (2010) The misuse of BLUP in ecology and evolution. American Naturalist, 175, 116–125.

Henderson, C.R. (1976) A simple method for computing the inverse of a numerator relationship matrix used in prediction of breeding values. Biometrics, 32, 69–83.

Kennedy, B.W., Schaeffer, L.R. & Sorensen, D.A. (1988) Genetic properties of animal models. Journal of Dairy Science, 71, 17–26.

Lynch, M. & Walsh, B. (1998) Genetics and Analysis of Quantitative Traits. Sinauer Associates, Incorporated, Sunderland, MA.

Meuwissen, T.H.E. & Luo, Z. (1992) Computing inbreeding coefficients in large populations. Genetics, Selection, Evolution, 24, 305–313.

Meyer, K. (2007) WOMBAT - a tool for mixed model analyses in quantitative genetics by restricted maximum likelihood (REML). Journal of Zhejiang University. Science. B, 8, 815–821.

Mrode, R.A. (2005) Linear Models for the Prediction of Animal Breeding Values, 2nd ed. CABI Publishing, Cambridge, MA.

Oikawa, T. & Yasuda, K. (2009) Inclusion of genetically identical animals to a numerator relationship matrix and modification of its inverse. Genetics, Selection, Evolution, 41, 25.

Quaas, R.L. (1976) Computing the diagonal elements and inverse of a large numerator relationship matrix. Biometrics, 32, 949–953.

Quaas, R.L. (1988) Additive genetic model with groups and relationships. Journal of Dairy Science, 71, 1338–1345.

Quaas, R.L. (1995) Fx algorithms. An unpublished note.

Reid, J.M. & Keller, L.F. (2010) Correlated inbreeding among relatives: occurrence, magnitude, and implications. Evolution, 64, 973–985.

Robinson, G.K. (1986) Group effects and computing strategies for models for estimating breeding values. Journal of Dairy Science, 69, 3106–3111.

Schaeffer, L.R. (1991) C. R. Henderson: Contributions to predicting genetic merit. Journal of Dairy Science, 74, 4052–4066.

Schaeffer, L.R. (1994) Multiple-country comparison of dairy sires. Journal of Dairy Science, 77, 2671–2678.

Schaeffer, L.R. (1999) Phantom parents. An unpublished note.

Sullivan, P.G. (1999) Estimating Genetic Variances and Covariances for Models with Genetic Groups. http://cgil.uoguelph.ca/dcbgc/Agenda9909/agenda9909.htm.

Westell, R.A., Quaas, R.L. & Van Vleck, L.D. (1988) Genetic groups in an animal model. Journal of Dairy Science, 71, 1310–1318.

Wolak, M.E. (2012) nadiv: an R package to create relatedness matrices for estimating non-additive genetic variances in animal models. Methods in Ecology and Evolution, 3, 792–796.

Wolak, M.E. & Keller, L.F. (2014) Dominance genetic variance and inbreeding in natural populations. Quantitative Genetics in the Wild (eds A. Charmantier, D. Garant & L.E.B. Kruuk), pp. 104–127. Oxford University Press, Oxford.

Wolak, M.E. & Reid, J.M. (2016) Data from: Accounting for genetic differences among unknown parents in microevolutionary studies: How to include genetic groups in quantitative genetic animal models. Journal of Animal Ecology, Dryad Digital Repository, http://dx.doi.org/10.5061/dryad.jf7cr.

Appendix S7: Fuzzy classification of genetic groups

Instead of assigning each phantom parent to a single genetic group, phantom parents can have a probability of belonging to each group (Fikse 2009). Strategies for assigning phantom parents probabilities of group membership are briefly discussed by Fikse (2009). In general, fuzzy classification strategies should reflect the underlying variables causing uncertainty in assigning phantom parents to genetic groups or the uncertainty in defining the genetic groups themselves. For example, unknown age or birth year of a phantom parent might preclude easy genetic group assignment in a longitudinal study where genetic groups are structured through time. Instead, phantom parents with unknown birth years can be assigned a probability of membership in multiple genetic groups spanning the time in which the phantom parent was likely born. Fuzzy classification can also be useful in such a context when the group classification itself is uncertain. An example of this would be when constructing genetic groups that are composed of all phantom parents occurring within five year intervals. The phantom parents born in the fifth year encompassed by group one may differ more genetically from phantom parents born in the first year of group one than they do from phantom parents born in the first year of group two. Additionally, such a structure might be even harder to define when generations are overlapping. Fuzzy classification then allows for these types of uncertainy to be incorporated into the analysis.

Below is an example of how to implement such fuzzy classification of phantom parents using `nadiv` in `R` to construct the $\mathbf{Q}$ and $\mathbf{A}^*$ matrices. We use the example pedigree from Quaas (1988) introduced above (*Appendix 6.1*), using the portion of this data that includes phantom parents and observed individuals. The subset of `Q1988` we will use is:

```
Q1988fuzz <- Q1988[-c(1:2), c("id", "phantomDam", "phantomSire")]
```

id	phantomDam	phantomSire
a	NA	NA
b	NA	NA
c	NA	NA
d	NA	NA
e	NA	NA
A	a	b
B	A	c
C	d	e
D	B	C

In the `id` column, lower case letters are phantom parent identities and upper case letters are observed individual identities. The second and third columns indicate an individual's dam and sire, respectively. Every observed individual (upper case letter) with an unknown parent is assigned a unique phantom parent instead of a missing value (e.g., `a` instead of NA). The phantom parents are assigned to individuals as in Quaas (1988). Phantom parents, however, have missing values (NA) assigned as their dams and sires. Additional details regarding the `Q1988` dataset in `nadiv` are in the `R` help file that can be viewed by running the command `?Q1988` in an `R` session. The next step is to assign phantom parents to genetic groups, specifically in such a way as to allow group membership to be probabilistic.

7.1 Constructing the fuzzy classification matrix F

When fuzzy classification of genetic group membership is used, unknown parents are no longer replaced with genetic groups in the pedigree. Instead, each unknown parent is assigned a unique phantom parent identity, these phantom parent identities are given their own entry/row in the pedigree. Also, a fuzzy classification (**F**) matrix that has p rows, one for each phantom parent, and r columns, one for each genetic group, needs to be constructed. The **F** matrix contains individual elements f_{ij} that take values between 0 and 1 and quantify the probability of phantom parent i belonging to genetic group j. The rows of **F** (i.e., a phantom parent's probabilities of group membership across all genetic groups) must sum to one.

In the example pedigree `Q1988fuzz`, **F** is a 5 row by 2 column matrix. Here, we arbitrarily assign fuzzy classification probabilities (f_{ij}). We exclusively assign phantom parent `a` to genetic group "g1" and `b` to genetic group "g2". We assign phantom parents `c`, `d`, `e` each a 0.5 probability of group membership in each of the two genetic groups.

```r
# Find the phantom parents in the pedigree Q1988fuzz
pp <- which(is.na(Q1988fuzz[, 2]))
# First, an empty F matrix
F <- matrix(0, nrow = length(pp), ncol = 2,
    dimnames = list(as.character(Q1988fuzz[pp, 1]), c("g1", "g2")))
# Assign "a" to "g1" and "b" to "g2"
F[cbind(match(c("a", "b"), Q1988fuzz[pp, 1]), c(1,2))] <- 1
# Assign rest of the phantom parents to both "g1" & "g2" with equal probability
F[match(c("c", "d", "e"), Q1988fuzz[pp, 1]), ] <- 0.5
F
```

```
##    g1  g2
## a 1.0 0.0
## b 0.0 1.0
## c 0.5 0.5
## d 0.5 0.5
## e 0.5 0.5
```

Now that the phantom parents have been assigned probabilities of genetic group membership, we construct the $\mathbf{Q}$ and $\mathbf{A}^*$ matrices. To help distinguish the approaches, we will call the resulting matrices created with fuzzy classification $\mathbf{Q_f}$ and $\mathbf{A_f^*}$. These will have the same properties as the $\mathbf{Q}$ and $\mathbf{A}^*$ matrices constructed above without fuzzy classification, however, the individual matrix elements will differ. The difference reflects the uncertainty in phantom parent group membership. If all phantom parents are assigned with complete probability to one genetic group each (i.e., each row of $\mathbf{F}$, corresponding to each of the phantom parents, contains a single value of one and the rest zeroes), then the resulting $\mathbf{Q_f}$ and $\mathbf{A_f^*}$ will be exactly the same as if they were constructed without using fuzzy classification. Further details of the method are in Fikse (2009), whereas below the focus is on how to implement fuzzy classification using the `nadiv` functions `ggcontrib()` to obtain $\mathbf{Q_f}$ and `makeAinv()` to obtain $\mathbf{A_f^*}$.

7.2 Constructing $\mathbf{Q_f}$

As described in the main text (and above), the matrix $\mathbf{Q}$ contains the fractional contributions from each of r genetic groups to each individual's genome, calcluated from the pedigree. With n observed individuals (i.e., not phantom parents) in the pedigree (e.g., n=4 in `Q1988fuzz`), the fuzzy classification matrix $\mathbf{Q_f}$ (the subscript "f" is used to distinguish this $\mathbf{Q}$ matrix as including fuzzy classification of phantom parents) is an n row by r column matrix. As detailed above (and in the main text), offspring inherit half of every genetic group contribution from each of their parents. Similarly, offspring of phantom parents inherit half of each phantom parents genetic group contributions. In the normal construction of $\mathbf{Q}$, phantom parents only belong to a single genetic group. With fuzzy classification, however, each phantom parent has a probability of group membership that can be non-zero for more than one group. These probabilities can be thought of as fractional contributions of each genetic group to each phantom parent's genome. Consequently, offspring of phantom parents inherit half of the phantom parents genetic group contributions described in $\mathbf{F}$ in exactly the same manner as offspring inherit all of the genetic group contributions from an observed parent quantified in a row of $\mathbf{Q}$.

The nadiv function ggcontrib() can construct $\mathbf{Q_f}$ for a given pedigree with r potential genetic group contributions to each of the n individuals in the pedigree when the fuzzy classification of phantom parents ($\mathbf{F}$) is supplied to the fuzz argument of the function. For example, $\mathbf{Q_f}$ for Q1988fuzz is obtained:

```
(Q_f <- ggcontrib(Q1988fuzz, fuzz = F))
```

```
##    g1  g2
## A 0.5 0.5
## B 0.5 0.5
## C 0.5 0.5
## D 0.5 0.5
```

Each row in $\mathbf{Q_f}$ (each entry is a proportional contribution) sums to one and offspring inherit half of each genetic group contribution from each of their parents. The result of including $\mathbf{F}$ is that both genetic groups contribute equally to every observed individual in the Q1988fuzz pedigree.

For example, individual A has phantom dam a and phantom sire b. Row one of the $\mathbf{Q_f}$ above is a sum of each phantom parents' row in $\mathbf{F}$ (probability of group membership or fractional contribution of each genetic group to the phantom parent) divided by two: phantom dam a contributes the following row in $\mathbf{F}$ [1.0 0.0]/2 plus phantom sire b contributes the following row in $\mathbf{F}$ [0.0 1.0]/2. In other words, individual A gets half of phantom dam a's "g1" group contribution (1.0/2) and its phantom sire b does not contribute to the proportion of A's genome derived from "g1". Similarly, phantom dam a does not contribute to the proportion of A's genome derived from "g2" and A gets half of phantom sire b's contribution from "g2" (1.0/2).

In a second example, individual B has observed dam A and phantom sire c. Therefore, row two of $\mathbf{Q_f}$ above is the sum of dam A's row in $\mathbf{Q_f}$ (genetic group contributions) divided by two and phantom sire c's row in $\mathbf{F}$ (fuzzy classification) divided by two: dam A's row in $\mathbf{Q_f}$ (just calculated above) [0.50 0.50]/2 plus phantom sire c's row in $\mathbf{F}$ [0.50 0.50]/2.

To implement an animal model with genetic group effects estimated explicitly as separate fixed regressions, where the phantom parents have fuzzy classification of genetic group membership, requires the $\mathbf{Q_f}$ matrix as just calculated. The implementation in the animal model is no different with fuzzy classification of genetic groups than without fuzzy classification, just the details of constructing $\mathbf{Q_f}$ differ slightly. Similarly, there is no difference when using $\mathbf{Q_f}$ with fuzzy classification of genetic groups to calculate breeding values ($\mathbf{a}$) from

an animal model that has genetic group effects implicitly within the random effects (see above and equation 6 in the main text).

7.3 Constructing $\mathbf{A}^*_\mathbf{f}$

Methods to directly construct $\mathbf{A}^{-1}$ and $\mathbf{A}^*$ rely on the basic premise that the flow of alleles through a pedigree can be traced because each individual inherits, on average, half of its alleles from each parent with some sampling variation that can be modeled based on the Mendelian sampling variance in the population. The same principles apply when phantom parents have fuzzy classification of genetic group membership. Therefore, the same methods to directly construct $\mathbf{A}^*$ without fuzzy classification can now be used to directly construct an augmented inverse relatedness matrix from a pedigree when fuzzy classification of genetic groups has been implemented (we will call this matrix $\mathbf{A}^*_\mathbf{f}$, where the subscript "f" is used to distinguish this matrix from $\mathbf{A}^*$ constructed without fuzzy classification of genetic groups) (Fikse 2009).

$\mathbf{A}^*_\mathbf{f}$ is also a compilation of four sub-matrices that are each a mathematical function of $\mathbf{Q_f}$ (as calculated directly above incorporating fuzzy classification), $\mathbf{A}^{-1}$, or both (see Fig. 3e in main text, replacing $\mathbf{Q}$ with the $\mathbf{Q_f}$ constructed with fuzzy classification).

The $\mathbf{A}^*_\mathbf{f}$ for any pedigree with phantom parents assigned can be obtained using the `makeAinv()` function in the `nadiv` package and supplying the fuzzy classification defined by $\mathbf{F}$ to the `fuzz` argument. For example, $\mathbf{A}^*_\mathbf{f}$ for the `Q1988fuzz` pedigree is:

```
Astar_fOut <- makeAinv(Q1988fuzz, fuzz = F)
```

```
(Astar_f <- Astar_fOut$Ainv)
```

```
## 6 x 6 sparse Matrix of class "dgCMatrix"
##                                          g1         g2
## A    1.3333333 -0.6666667  .    . -0.3333333 -0.3333333
## B   -0.6666667  1.8333333  0.5 -1 -0.3333333 -0.3333333
## C    .          0.5000000  1.5 -1 -0.5000000 -0.5000000
## D    .         -1.0000000 -1.0  2  .          .
## g1 -0.3333333 -0.3333333 -0.5  .  0.5833333  0.5833333
## g2 -0.3333333 -0.3333333 -0.5  .  0.5833333  0.5833333
```

To model genetic group effects, where the phantom parents have fuzzy classification of genetic group membership, implicitly within the random effects of an animal model, $\mathbf{A_f^*}$ is supplied to the model the same way as the typical $\mathbf{A^{-1}}$ or $\mathbf{A}^*$ (see tutorials above). It remains to be seen if the same alterations to $\mathbf{A_f^*}$ are also necessary as when fitting $\mathbf{A}^*$ in an animal model (see *Appendices 6.3.1 and 6.3.2*).

References: Appendix S7

Fikse, F. (2009) Fuzzy classification of phantom parent groups in an animal model. Genetics, Selection, Evolution, 41, 42–49.

Software version information

The following software versions were used to run all of the code above:

```
R.version.string
```

```
## [1] "R version 3.3.1 (2016-06-21)"
```

```
packageVersion("nadiv")
```

```
## [1] '2.14.3.1'
```

```
packageVersion("MCMCglmm")
```

```
## [1] '2.22.1'
```

```
packageVersion("asreml")
```

```
## [1] '3.0'
```

WOMBAT: Linux 64-bit version 12 June 2015, compiled using the `ifort` compiler with MKL library (threaded)

ASReml: ASReml 4.10 [28 Dec 2014] lr [18 Mar 2015], from asreml-4.1.0.1051-lr.64.tgz
